# Carbonic anhydrase inhibition ameliorates tau toxicity via enhanced tau secretion

**DOI:** 10.1038/s41589-024-01762-7

**Published:** 2024-10-31

**Authors:** Ana Lopez, Farah H. Siddiqi, Julien Villeneuve, Rodrigo Portes Ureshino, Hee-Yeon Jeon, Philippos Koulousakis, Sophie Keeling, William A. McEwan, Angeleen Fleming, David C. Rubinsztein

**Affiliations:** 1 Department of Medical Genetics, University of Cambridge, Cambridge Institute for Medical Research, Cambridge, UK; 2 Department of Physiology, Development and Neuroscience, University of Cambridge, Cambridge, UK; 3 UK Dementia Research Institute, University of Cambridge, Cambridge Institute for Medical Research, Cambridge, UK

**Keywords:** Neurodegenerative diseases, Phenotypic screening, Protein aggregation, Screening

## Abstract

Tauopathies are neurodegenerative diseases that manifest with intracellular accumulation and aggregation of tau protein. These include Pick’s disease, progressive supranuclear palsy, corticobasal degeneration and argyrophilic grain disease, where tau is believed to be the primary disease driver, as well as secondary tauopathies, such as Alzheimer’s disease. There is a need to develop effective pharmacological therapies. Here we tested >1,400 clinically approved compounds using transgenic zebrafish tauopathy models. This revealed that carbonic anhydrase (CA) inhibitors protected against tau toxicity. CRISPR experiments confirmed that CA depletion mimicked the effects of these drugs. CA inhibition promoted faster clearance of human tau by promoting lysosomal exocytosis. Importantly, methazolamide, a CA inhibitor used in the clinic, also reduced total and phosphorylated tau levels, increased neuronal survival and ameliorated neurodegeneration in mouse tauopathy models at concentrations similar to those seen in people. These data underscore the feasibility of in vivo drug screens using zebrafish models and suggest serious consideration of CA inhibitors for treating tauopathies.

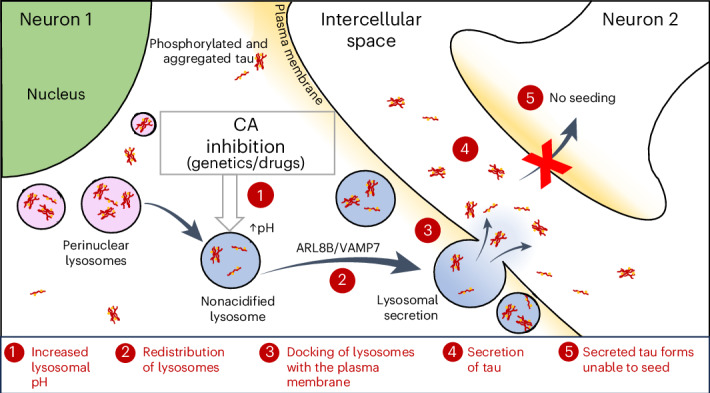

## Abstract (web summary)

A compound screen using zebrafish identified carbonic anhydrase (CA) as a modulator of tau toxicity. CA inhibition abrogates lysosomal acidification and causes tau secretion by lysosomal exocytosis without having a prion effect. This mechanism lowers intracellular tau and is neuroprotective in zebrafish and mouse models of tauopathy.

## Main

Tauopathies are neurodegenerative diseases characterized by the intracellular accumulation and aggregation of tau protein. These include Pick’s disease, progressive supranuclear palsy, corticobasal degeneration and argyrophilic grain disease, where tau is believed to be the primary disease driver. Although many tauopathies are not caused by primary tau gene mutations, such genetic lesions cause the autosomal-dominant disorder frontotemporal dementia with parkinsonism 17 and, along with mouse studies^1–3^, provide strong support that tau variants themselves can cause disease via gain-of-toxic-function mechanisms. Thus, primary tauopathies may have pathological similarities with other monogenic neurodegenerative conditions caused by mutations that increase the accumulation and aggregation of their causative proteins, like mutant α-synuclein in familial Parkinson’s disease and mutant huntingtin in Huntington’s disease. Secondary tauopathies include Alzheimer’s disease and chronic traumatic encephalopathy, where the tau accumulation appears to be secondary to a distinct upstream cause but nevertheless is believed to be an important driver of neurodegeneration.

Due to the high incidence of these diseases, there is a need to develop effective pharmacological therapies. To accelerate drug discovery, multiple compound libraries that include clinically approved drugs have been assembled, as such drugs have the potential to be repurposed for novel indications. Because they have been previously well studied in preclinical and human contexts, they can traverse the drug discovery pipeline more rapidly than new composition of matter^4–7^.

Most large compound screens aiming to identify tauopathy modulators have been performed in cell culture and usually used cancer cell lines^8–12^. However, such models do not faithfully recapitulate the in vivo environment with its diversity of cell types. To assess compound efficacy, we wanted to use a vertebrate model where there was overt toxicity but that was also amenable to moderate-throughput screening. Thus, we used a transgenic zebrafish model of tauopathy expressing human wild-type tau in the fish retina. As a consequence of tau-derived toxicity, retinal cells expressing the transgene degenerate over the course of a few days, making it a suitable model to investigate modulators of tau pathology in vivo. This model was previously validated for assaying drug effects in modulating tau protein clearance and reducing tau toxicity^4,13,14^. This model was screened using the Johns Hopkins Clinical Compound Library (JHCCL) comprising Food and Drug Administration (FDA)-approved drugs and compounds used in clinical trials, which was initially compiled to identify new inhibitors of the malaria parasite *Plasmodium falciparum*^15^.

## Results

**Extended Data Fig. 1:**
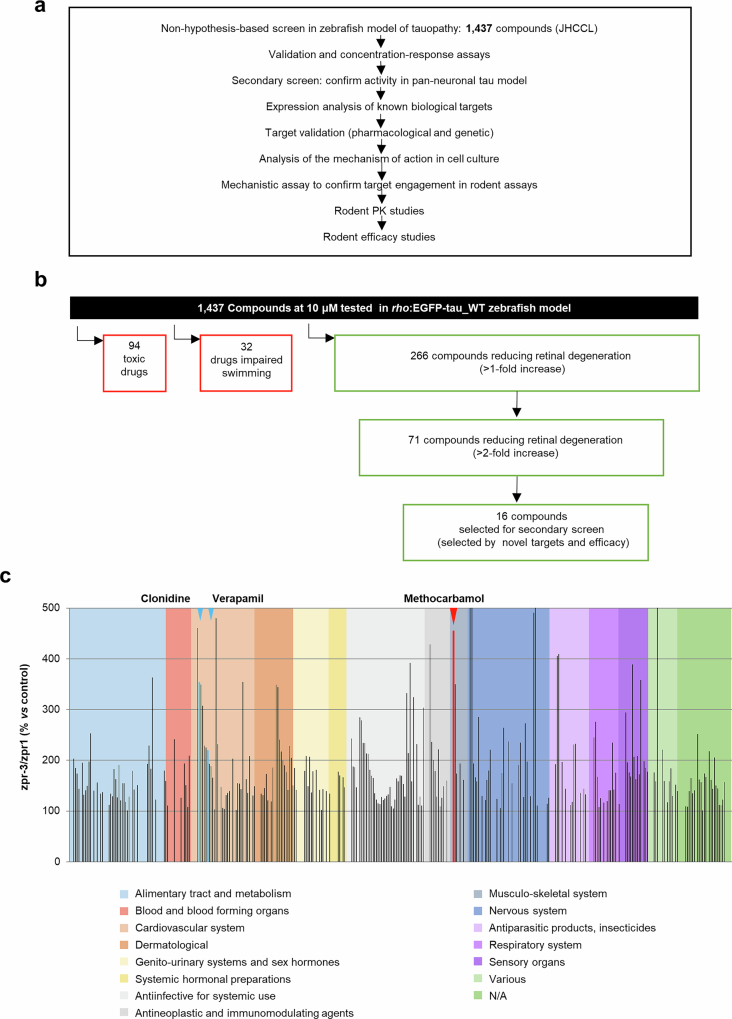
Summary compound of screen. **a**) General workflow from the primary screen study to mouse experiments used to identify carbonic anhydrases as new tau-toxicity modulators using the Johns Hopkins Clinical Compound Library (JHCCL). **b**) Chart representing the numbers of compounds evaluated during the primary screen using the zebrafish retinal-tau model rho:EGFP-tau_WT and subsequent selection and for secondary screen in models with pan-neuronal expression of Dendra-tau. **c**) Graph representing the quantification of the rescue of photoreceptor degeneration in the *rho*:eGFP–tau-WT model for all 266 compounds from the primary screen that increased the zpr3/zpr1 ratio over a 100% compared to control fish treated either with water or DMSO. Drugs are color-coded according to the organ system that they are known to target in their current clinical indication. Red arrowhead on the top of the graph points to the hit further characterized in this paper (methocarbamol). Blue arrowheads highlight clonidine and verapamil, known autophagy modulators which have previously been shown to reduce tau toxicity. These data validate the efficacy of the primary screen. Source data

The experimental flow for this study is represented in Extended Data Fig. 1.

### Compound screen to identify new tauopathy modulators

In total, 1,437 drugs from the JHCCL were tested in a hypothesis-free screen using a zebrafish model that expresses enhanced green fluorescent protein (eGFP)-labeled human wild-type tau in rod photoreceptors (*rho*:eGFP–tau-WT), which is characterized by a progressive rod photoreceptor degeneration^14^. Transgenic larvae were treated with compounds at a concentration of 10 μM from 6 to 10 days postfertilization (d.p.f.). Ninety-four compounds caused fish lethality and were discarded from further analysis (Extended Data Fig. 1b and Supplementary Table 1). Thirty-two compounds caused swimming impairments but were not excluded from the analysis. After drug treatments, the levels of rod degeneration were analyzed by western blotting measuring the rhodopsin:arrestin (zpr-3:zpr-1) ratio as a readout of rod survival. The results of the primary screen were expressed as a percent increase in the zpr-3:zpr-1 ratio relative to control larvae treated with DMSO. An increase of >100% in the zpr-3:zpr-1 ratio indicated an amelioration of rod photoreceptor degeneration and a potential decrease in tau toxicity (Extended Data Fig. 1c and Supplementary Table 1). This subset of compounds comprises FDA-approved drugs targeting diverse systems according to the therapeutic indication listed in the Merck Index (Extended Data Fig. 1c). Seventy-one compounds increased the survival of tau-expressing rods by twofold and included clonidine and verapamil, autophagy modulators known to reduce tau toxicity^4,16,17^, providing an internal control for the primary screen (Extended Data Fig. 1c). Using publicly available drug databases, we identified the number of predicted biological targets for each of the 71 compounds and prioritized those that have five or fewer known biological targets. We then used literature analysis to further limit the list to targets that had not previously been reported to be effective in neurodegenerative disease models. This provided 16 compounds for further validation and concentration–response assays. The rescue effect of the 16 hits in the retinal model of tauopathy was confirmed by assessing the morphological changes in a second transgenic zebrafish model in which human mutant tau-A152T is fused to the photoconvertible protein Dendra2 and is expressed throughout the central nervous system (CNS)^17^.

### Identification of methocarbamol from the compound screen

**Extended Data Fig. 2:**
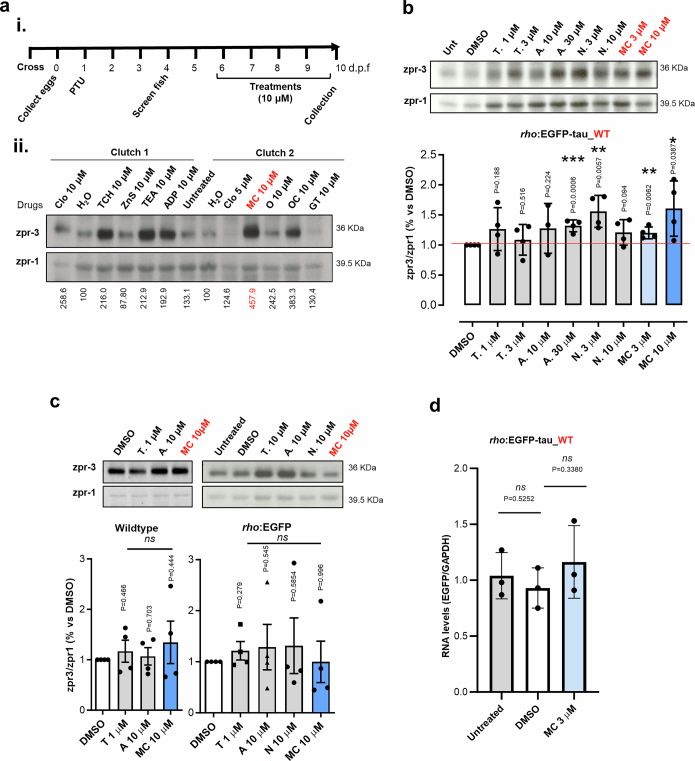
Methocarbamol identified as a primary hit from the compound screen. **a**. i. Schematic representation of the drug treatment protocol used for the primary screen. ii. Images of the western blots with samples from 2 clutches from the primary screen identifying methocarbamol (MC 10 μM) as showing strong rescue of tau-induced retinal degeneration in the *rho*:eGFP–tau-WT model. Levels of zpr-3 are increased after methocarbamol treatment compared to the water control with similar levels zpr-1. (n = 1; all drugs were tested once during the primary screen. Ratio zpr-3/zpr-1 given below the blot). Clonidine (Clo), Tetracycline hydrochloride (TCH), Zinc sulfate (ZnS), Tetraethylammonium chloride hydrate (TEA), Ammonium phosphate dibasic (APD), Orphenadrine (O), Orphenadrine citrate (OC) and Gallamiene triethidide (GT). **b**.Western blots and quantification for zpr-3/zpr-1 in homozygous *rho*:eGFP–tau-WT fish treated with 3 and 10 μM methocarbamol (MC) or DMSO among other drugs during validation experiments. Methocarbamol treatment results in higher zpr-3/zpr-1 ratios, hence rescues tau-induced loss of photoreceptors (n = 4 experiments; graph represents mean ± SEM; *p ≤ 0.05, **p≤ 0.01 and ***p≤ 0.001 vs. DMSO by two-tailed one-sample t-test). (T) temozolamide; (A) aminoglutethimide; (N) nitrofurantoin. **c**.Western blots and quantification for zpr-3/zpr-1 in wild-type and *rho*:eGFP fish treated with 10 μM methocarbamol (MC) or DMSO among other drugs during validation experiments. Methocarbamol does not induce significant changes in the relative rod/cone content in non-tauopathy models (n = 4 experiments; graph represents mean ± SEM; *ns p≥0.05 vs. DMSO* analysed by two-tailed unpaired t-test). (T) temozolamide; (A) aminoglutethimide; (N) nitrofurantoin. **d**.Graph representing the mRNA levels of EGFP normalized to GAPDH by qPCR in *rho*:eGFP–tau-WT fish treated with 3 μM methocarbamol (MC) compared control conditions. Expression levels were normalized to DMSO-treated values. No significant changes in transgene expression were found after methocarbamol treatment indicating that results from the primary screen correspond to the rescue of photoreceptor degeneration rather than changes in transgene expression (n = 3 pools of 10 fish each; graph represents mean ± SD; *ns p≥0.05 vs. DMSO* analysed by two-tailed unpaired t-test). Source data

One of the most promising drugs identified from the primary screen was methocarbamol, resulting in an increase in the zpr-3:zpr-1 ratio of 457.9% in *rho*:eGFP–tau-WT heterozygous fish, compared with vehicle control-treated fish in the primary screen (Extended Data Fig. 2a). Concentration–response assays were then performed on *rho*:eGFP–tau-WT homozygous fish, which displayed a greater loss of photoreceptors than the heterozygotes used in the primary screen due to the increased expression of tau, and concentrations of 3 and 10 μM methocarbamol were able to rescue degeneration in this model (Extended Data Fig. 2b). To confirm that this effect was due to rescue of degeneration rather than the drug influencing transgene expression or rod development, we confirmed that treatment with 10 μM methocarbamol did not affect rhodopsin protein levels (zpr-3:zpr-1 ratio) in wild-type or *rho*:eGFP fish lines (Extended Data Fig. 2c). Moreover, the mRNA levels of the eGFP–tau transgene were unchanged following treatment with 3 μM methocarbamol from 6 to 10 d.p.f. (Extended Data Fig. 2d), suggesting that methocarbamol does indeed rescue tau-induced photoreceptor degeneration. Because 3 µM methocarbamol showed a significant rescue in our initial concentration–response assay, 3 µM methocarbamol was used in subsequent experiments on retinal tau models.

### Methocarbamol ameliorates tau-induced toxicity in zebrafish

**Fig. 1:**
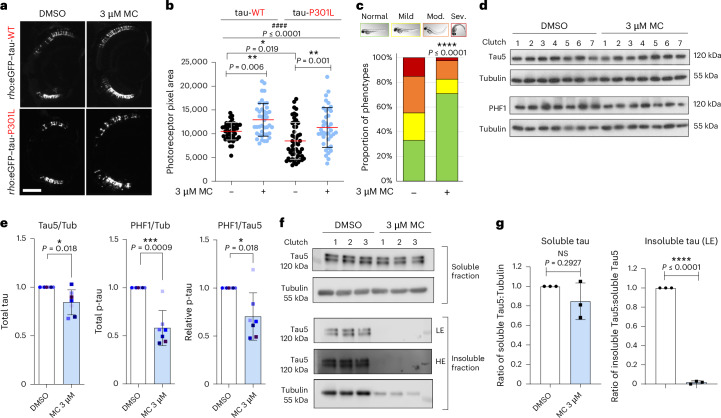
Methocarbamol ameliorates tau-induced toxicity and reduces hyperphosphorylated and insoluble tau levels in zebrafish models. **a**, Representative images of retinal tissue from *rho*:eGFP–tau-WT and *rho*:eGFP–tau-P301L fish showing increased rod survival after treatment with 3 μM methocarbamol (MC). Scale bar, 50 μm. **b**, Quantification of fluorescent photoreceptor area (as shown in **a**) following treatment with 3 μM methocarbamol. Drug treatment increased the pixel area of eGFP–tau^+^ photoreceptors (*n* = minimum of 38 eyes per condition; data are shown as mean ± s.d.). Data were analyzed by one-way ANOVA, ^####^*P* ≤ 0.0001, followed by Tukey’s multiple comparisons test with the following significance values: **P ≤ 0.01 versus DMSO (–), *P ≤ 0.01 for tau-WT (–) versus tau-P301L (–). **c**, Proportion of morphological abnormalities in fish with pan-neuronal expression of Dendra–tau-A152T after treatment with 3 μM methocarbamol. Phenotypes were ranked as normal (green), mild (yellow), moderate (Mod.; orange) and severe (Sev.; red) according to the severity of deformities (representative images are shown above the graph). Methocarbamol reduced the proportion of fish in the severe category while increasing the proportion of normal fish compared with DMSO-treated siblings (–); *n* = 13 clutches with a minimum of 30 fish per group; ****P ≤ 0.0001 versus DMSO (–). Data were analyzed by *χ*^2^ test. **d**,**e**, Images (**d**) and quantification (**e**) of western blots showing levels of total (Tau5) and hyperphosphorylated tau (PHF1) in fish with pan-neuronal expression of Dendra–tau-A152T after treatment with 3 μM methocarbamol. Methocarbamol reduces the levels of total tau (Tau5/tubulin), total phospho-tau (p-tau; PHF1/tubulin) and relative amounts of phosphorylated tau (that is, the normalized ratio of (PHF1/tubulin)/(Tau5/tubulin) represented as PHF1/tau); *n* = 7 independent clutches with 10 fish each ± s.d.; *P ≤ 0.05 versus DMSO; ***P ≤ 0.001 versus DMSO. Data were analyzed by two-tailed one-sample *t*-test; Tub, tubulin. **f**,**g**, Images (**f**) and quantification (**g**) of western blots for the sarkosyl-soluble and sarkosyl-insoluble fractions of tau in fish 6 d.p.f. with pan-neuronal expression of Dendra–tau-A152T treated with either DMSO or 3 μM methocarbamol. Graphs show mean ratios (±s.d.) of soluble tau versus tubulin and insoluble tau versus soluble tau normalized to the mean DMSO value. Three independent clutches per condition were used; *n* = 50 fish per group; ****P ≤ 0.0001 versus DMSO; NS, not significant. Data were analyzed by two-tailed one-sample *t*-test. Source data

**Extended Data Fig. 3:**
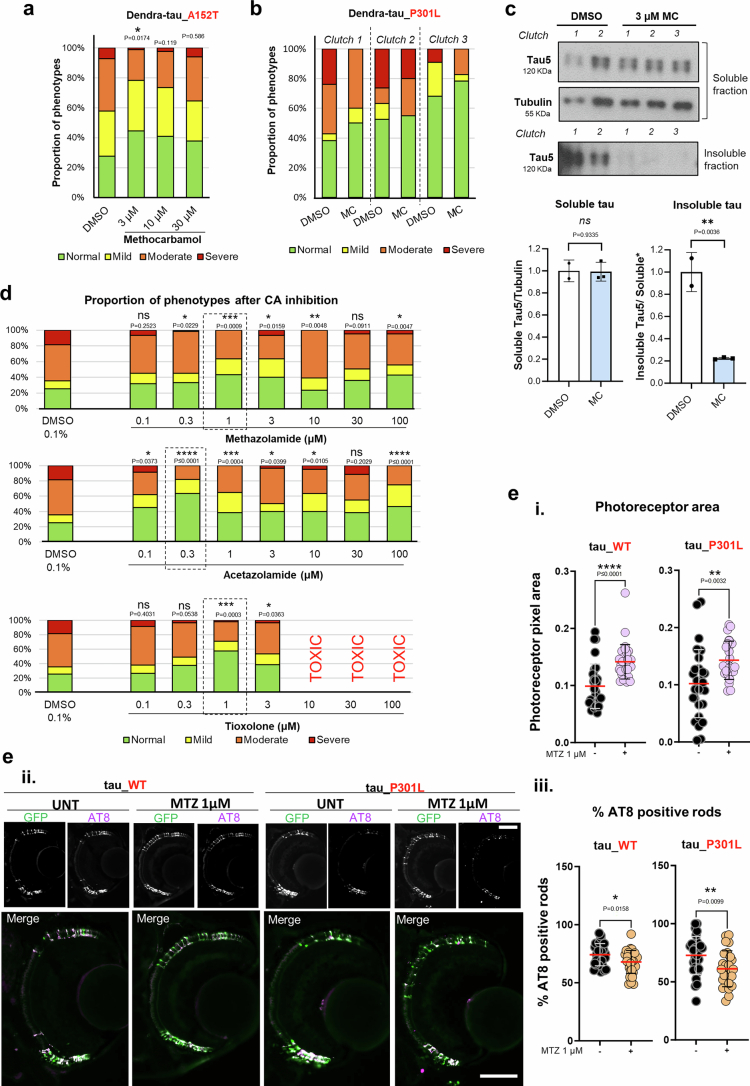
Phenotypic rescue and reduced insoluble tau by CA inhibitors in Dendra-tau models. **a**. Concentration-dependence of methocarbamol (MC) on morphological abnormalities in fish with pan-neuronal expression of Dendra-tau-A152T at 3 d.p.f., 3 μM is most effective concentration. (n = 3 independent clutches 20 fish/group; *p≤ 0.05 vs. DMSO; Chi-square test). **b**. Quantification of morphological abnormalities in 3 clutches with pan-neuronal expression of Dendra-tau-P301L after 3 μM MC treatment at 3 d.p.f. MC reduced proportions of abnormal phenotypes in all clutches. (n = 3 with fish/clutch). **c**. Images and quantification of western blots for sarkosyl-soluble and -insoluble tau fractions in 6 d.p.f. fish with pan-neuronal Dendra-tau-A152T expression treated with DMSO or 3 μM MC. Graphs show mean ratios (±SD) of soluble tau *vs*. tubulin and insoluble tau *vs* soluble tau normalised to mean DMSO value (soluble*).(n = 2 independent clutches for DMSO and 3 for MC with 50 fish/group; **p≤ 0.01 vs. DMSO; two-tailed unpaired t-test). **d**. Dose-dependent effect of carbonic anhydrase inhibitors methazolamide, acetazolamide and tioxolone on phenotypic abnormalities in fish with pan-neuronal expression of Dendra-tau-A152T at 3 d.p.f. All tested carbonic anhydrase inhibitors rescued phenotypic defects. Bars within dashed rectangles were the selected concentrations for experiments represented in main Fig. 2a. with extra replicates (n = 3 clutches with 20 fish/treatment; *p≤ 0.05 vs. DMSO; **p≤ 0.01 vs. DMSO and ***p≤ 0.001 vs. DMSO; Chi-square test). **e**) 1 µM methazolamide (MTZ) reduced proportion of AT8-positive photoreceptors in 10 d.p.f. *rho*:eGFP–tau-WT and -P301L fish: i. Quantification of photoreceptor area in images used for the AT8 immunohistochemistry showing rescue effect of MTZ. ii. Representative images from retinal sections used to quantify the proportion of AT8-positive rods. Top images represent GFP and AT8 fluorescence images separately. Larger images at the bottom correspond to merged images of the two signals. Green photoreceptors have none or low levels of tau phosphorylation whereas white or magenta rods represent the AT8-positive photoreceptor subpopulation (scale bar = 50 μm); iii. Quantification of proportion of green photoreceptors with AT8-positive staining upon 1 μM MTZ; For i, ii and iii (n = 17 eyes/condition; graphs represent mean ± SD; ****p≤ 0.0001, **p≤ 0.01 and *p≤ 0.05 vs. DMSO (-); two-tailed unpaired t-test). Source data

To further investigate the effect of methocarbamol in different models of tauopathy, quantification of photoreceptor cell loss was analyzed in transgenic fish expressing either wild-type (*rho*:eGFP–tau-WT) or mutant tau-P301L (*rho*:eGFP–tau-P301L), a disease-causing variant that results in greater degeneration in our retinal models. Treatment with 3 μM methocarbamol led to a significant rescue of photoreceptor degeneration (Fig. 1a,b). We next investigated the effects of methocarbamol in a transgenic model in which human tau-A152T is expressed throughout the CNS and results in morphological defects along the spine and body axis^17^. All concentrations of methocarbamol tested decreased the proportion of larvae with morphological abnormalities, with 3 μM methocarbamol being the most effective concentration (Extended Data Fig. 3a and Fig. 1c). Similar rescue was observed in larvae expressing tau-P301L (Extended Data Fig. 3b). Treatment with 3 μM methocarbamol also reduced total tau (Tau5/tubulin) and total hyperphosphorylated tau (PHF1/tubulin) levels (Fig. 1d,e). Moreover, treatment of these zebrafish with 3 μM methocarbamol significantly reduced the levels of sarkosyl-insoluble tau (Fig. 1f,g and Extended Data Fig. 3c).

### Carbonic anhydrases mediate methocarbamol-induced rescue

**Fig. 2:**
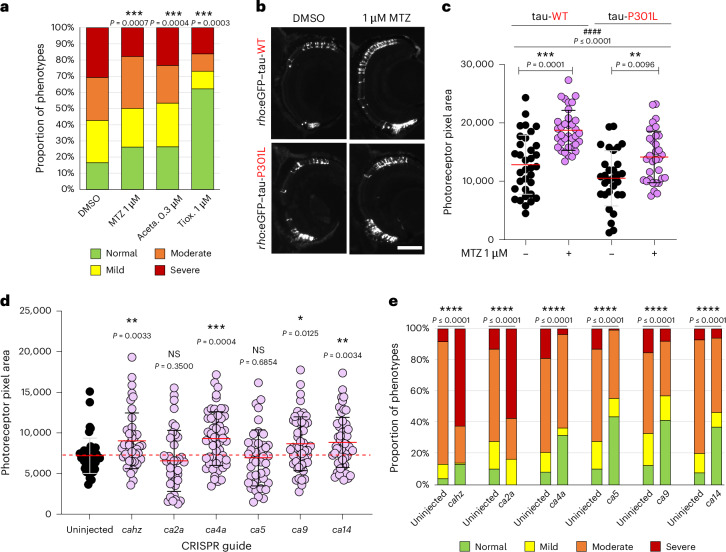
The rescuing effect of methocarbamol relies on its primary pharmacological target, the CA family. **a**, Proportion of morphological abnormalities in fish with pan-neuronal expression of Dendra–tau-A152T after treatment with 1 μM methazolamide (MTZ), 0.3 μM acetazolamide (Aceta.) or 1 μM tioxolone (Tiox.). Treatment with all CA inhibitors resulted in phenotypic rescue compared with DMSO-treated siblings (*n* = 6 clutches with ≥20 fish in each group; ***P ≤ 0.001 versus DMSO. Data were analyzed by *χ*^2^ test. **b**,**c**, Representative images of the retina of transgenic *rho*:eGFP–tau-WT and *rho*:eGFP–tau-P301L fish (**b**) and quantification of the pixel area of eGFP–tau^+^ photoreceptors following treatment with 1 μM methazolamide (**c**), showing an increase in eGFP–tau^+^ photoreceptors after treatment with methazolamide relative to DMSO-treated siblings (*n* ≥ 32 eyes per condition; the graph shows mean ± s.d.). Data were analyzed by one-way ANOVA, ^####^*P*≤ 0.0001, followed by Tukey’s multiple comparisons test with the following significance values: ***P ≤ 0.001 versus DMSO (–) for wild-type tau and **P ≤ 0.01 versus DMSO (–) for tau-P301L. Scale bar, 50 μm. **d**, Quantification of the pixel area of eGFP–tau^+^ rod photoreceptors following CRISPR injection. CRISPR targeting of *cahz*, *ca4a*, *ca9* and *ca14* increased the area of rod photoreceptors relative to uninjected siblings (for representative images, see Extended Data Fig. 4e; *n* ≥ 39 eyes per condition; data are shown as mean ± s.d.); *P ≤ 0.05, **P ≤ 0.01 and ***P ≤ 0.001 versus uninjected. Data were analyzed by two-tailed unpaired *t*-test. **e**, Proportion of morphological abnormalities in fish with pan-neuronal expression of Dendra–tau-A152T following CRISPR-based knockdown of CA isoforms. Genetic inhibition of *ca4a*, *ca5*, *ca9* and *ca14* increased the proportion of normal phenotypes, whereas CRISPR injection targeting *cahz* and *ca2* worsened the morphological defects compared with uninjected siblings (*n* = 3 clutches for toxic conditions (*cahz* and *ca2*) and *n* ≥ 4 clutches with a minimum of 30 fish per clutch for *ca4a*, *ca5*, *ca9* and *ca14*); ****P ≤ 0.0001 versus uninjected. Data were analyzed by *χ*^2^ test. Source data

Methocarbamol is described as a carbonic anhydrase (CA) I inhibitor and has also been predicted to interact with other CA isoforms according to drug databases (DrugBank and ChEMBL). We therefore investigated whether CA modulation was the mechanism by which methocarbamol ameliorated tau toxicity in our zebrafish models. We performed concentration–response experiments with a range of CA inhibitors (methazolamide, acetazolamide or tioxolone) in our pan-neuronal Dendra-tau-A152T tauopathy model and observed significant rescue of morphological abnormalities with all the drugs tested (Fig. 2a and Extended Data Fig. 3d). In addition, treatment with 1 μM methazolamide rescued the retinal degeneration observed in the *rho*:eGFP–tau-WT and *rho*:eGFP–tau-P301L models (Fig. 2b,c) and reduced the number of AT8^+^ photoreceptors (Extended Data Fig. 3e), suggesting that methazolamide also reduced the levels of hyperphosphorylated tau in these models.

**Extended Data Fig. 4:**
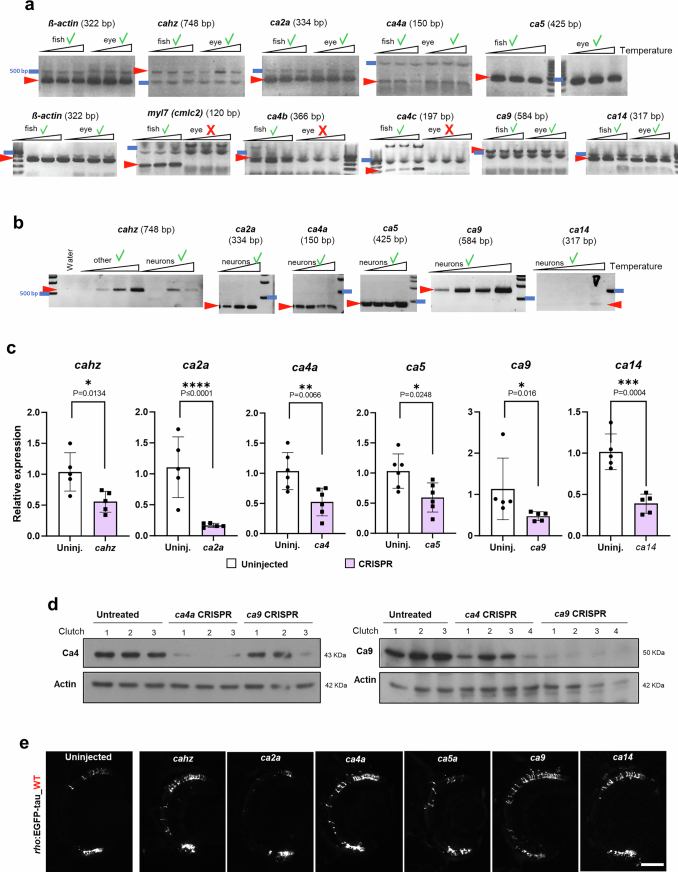
Carbonic anhydrase orthologue expression in zebrafish. **a**. Images of agarose gels with PCR products amplified from cDNA generated using RNA from 9 d.p.f. *rho*:eGFP–tau-WT fish. The endogenous expression of carbonic anhydrases ca2a, cahz, ca4a, ca4b, ca4b, ca5, ca9 and ca14 was assessed in whole fish versus dissected eyes (100 bp DNA ladder used). All isoforms except ca4b and ca4c could be detected in eyes and hence suitable to target in tauopathy retinal models (see green mark for isoforms band detected and a red cross for absence of PCR product at the right size indicated by a red arrowhead). **b**. Images of agarose gels with PCR products amplified from cDNA using RNA from FAC-sorted neurons from fish with pan-neuronal expression of Dendra-tau-A152T. The endogenous expression of carbonic anhydrases in ca2a, cahz, ca4a, ca5, ca9 and ca14 was assessed by rt-PCR (1 Kb DNA ladder used). All isoforms were detected in neurons and hence suitable to be targeted in the pan-neuronal Dendra-tau model. **c**. qPCR results showing the decreased expression of cahz, ca2a, ca4a, ca5, ca9 and ca14 in whole fish after injection of CRISPR guide RNAs targeting those genes (RNA collected from pools of 10 embryos from 5 independent clutches run in triplicate; *p≤ 0.05, **p≤ 0.01; ***p≤ 0.001 and ****p≤ 0.0001 vs. uninjected siblings analysed by two-tailed unpaired t-test). **d**. Images of western blots showing the decrease in ca4 and ca9 protein after CRISPR/Cas9 injection in wild-type fish. A minimum of 3 independent clutches were run for each group in a single gel. **e**. Representative images of cryosections across the central retina of transgenic *rho*:eGFP–tau-WT injected with CRISPRs targeting carbonic anhydrases *cahz, ca2a, ca4a, ca5, ca9* and *ca14* quantified in Fig. 2d. The knockdown of *cahz, ca4a, ca9* and *ca14* resulted in an increased number of fluorescent photoreceptors that is, reduced degeneration. Scale bar represents 50 μm. Source data

There are 15 CAs in humans and 20 in zebrafish, with 11 of the human genes having at least one zebrafish ortholog. To investigate whether CAs were expressed in the relevant zebrafish tissues for our studies, reverse transcription polymerase chain reaction (RT–PCR) was performed using RNA extracted from (1) dissected eyes from *rho*:eGFP–tau-WT fish at 9 d.p.f. compared with their whole bodies and (2) neurons sorted by fluorescence-activated cell sorting (FACS) from fish at 3 d.p.f. with pan-neuronal expression of Dendra–tau-A152T. Primers were designed for the most abundant cytosolic isoforms *ca2a* and *cahz*; membrane-bound isoforms *ca4a*, *ca4b*, *ca4c*, *ca9* and *ca14* and the mitochondrial ortholog *ca5*. Expression of zebrafish orthologs *ca2a*, *cahz*, *ca4a*, *ca5*, *ca9* and *ca14* was detected in the appropriate tissue locations for both models (that is, in the eyes and in neurons, respectively; Extended Data Fig. 4a,b). We next aimed to validate the role of these targets in ameliorating tau toxicity using genetic inhibition. CRISPR–Cas9 injections using guide RNAs targeting *ca2a*, *cahz*, *ca4a*, *ca5*, *ca9* or *ca14* resulted in diminished endogenous expression levels for all studied genes after 3 days (Extended Data Fig. 4c). In addition, reduced Ca4 and Ca9 protein levels could also be detected by western blotting (Extended Data Fig. 4d). CRISPR–Cas9 injection into *rho*:eGFP–tau-WT embryos ameliorated photoreceptor loss after 10 days under *cahz*-, *ca4a*-, *ca9*- and *ca14*-knockdown conditions (Fig. 2d and Extended Data Fig. 4e). In addition, CRISPR knockdown of *ca4a*, *ca5*, *ca9* and *ca14* expression rescued the phenotypic defects seen in fish with pan-neuronal expression of Dendra–tau-A152T (Fig. 2e). These results suggest that pharmacological and genetic manipulation of CAs protects against tau-induced toxicity in zebrafish models.

### CA inhibition increases the clearance rate of tau

**Fig. 3:**
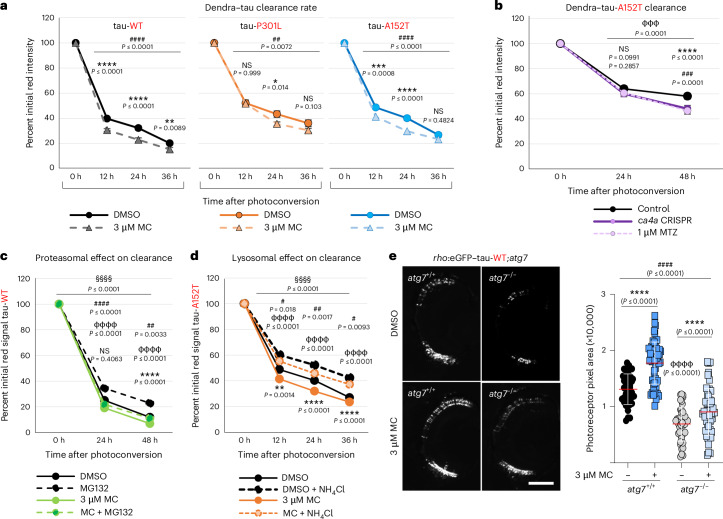
CA inhibition increases the clearance rate of tau without affecting proteasomal or autophagic degradation in vivo. **a**, Clearance kinetics of Dendra–tau in fish expressing tau-WT, tau-P301L or tau-A152T treated with DMSO (continuous lines) or 3 μM methocarbamol (dashed lines). All Dendra–tau forms cleared at a significantly faster rate following methocarbamol treatment than following treatment with DMSO (*n* ≥49 neurons per group; data are shown as mean ± s.d.). Data were analyzed by two-way ANOVA (^##^*P* ≤ 0.01 and ^####^*P* ≤ 0.0001) followed by Sidak’s multiple comparisons, *P ≤ 0.05, **P ≤ 0.01, ***P ≤ 0.001 and ****P ≤ 0.0001 versus DMSO. **b**, Clearance kinetics of Dendra–tau-A152T in fish treated with 1 μM methazolamide or injected with CRISPR guide RNA targeting *ca4a* compared with uninjected fish (treated with DMSO). Treatment with methazolamide or *ca4a* genetic inhibition increased the clearance of Dendra–tau-A152T (*n* ≥ 57 neurons per group; data are shown as mean ± s.d.). Data were analyzed by one-way ANOVA, ^ɸɸɸ^*P* ≤ 0.001, followed by Sidak’s multiple comparisons test with the following significance values: ****P ≤ 0.0001 versus control for *ca4a* CRISPR and ^*###*^*P* ≤ 0.001 versus control for methazolamide. **c**, Clearance kinetics of Dendra–tau-WT in fish treated with 3 μM methocarbamol with or without the proteasome inhibitor MG132 (10 μM). MG132 delayed the clearance of tau-WT, whereas methocarbamol increased tau clearance. However, tau-WT clearance in the presence of methocarbamol + MG132 was significantly higher than that observed in the presence of MG132 alone, suggesting that methocarbamol can accelerate tau clearance when proteasomal degradation is inhibited (*n* ≥ 69 neurons per group; data are shown as mean ± s.d.). Data were analyzed by one-way ANOVA, ^§§§§^*P* ≤ 0.0001, followed by Sidak’s multiple comparisons test with the following significance values: ^*##*^*P* < 0.01 and ^*####*^*P* < 0.0001 for DMSO versus methocarbamol; ^ɸɸɸɸ^*P* ≤ 0.0001 for DMSO versus MG132; ****P ≤ 0.0001 for methocarbamol versus methocarbamol + MG132. **d**, Effect of methocarbamol on lysosomal tau clearance kinetics of Dendra–tau-A152T in the presence or absence of 10 μM NH_4_Cl. NH_4_Cl blocks lysosomal acidification and delays the clearance of tau-A152T, whereas 3 μM methocarbamol accelerates it. Tau is cleared more rapidly when methocarbamol is combined with NH_4_Cl than with NH_4_Cl alone, suggesting that methocarbamol-improved tau clearance kinetics do not entirely depend on lysosomal degradation (*n* ≥ 60 neurons per group; data are shown as mean ± s.d.). Data were analyzed by one-way ANOVA, ^§§§§^*P* ≤ 0.0001, followed by Sidak’s multiple comparisons test with the following significance values: **P ≤ 0.01 and ****P ≤ 0.0001 for DMSO versus 3 μM methocarbamol; ^*#*^*P* < 0.05 and ^*##*^*P* < 0.01 for DMSO + NH_4_Cl versus methocarbamol + NH_4_Cl; ^ɸɸɸɸ^*P* ≤ 0.0001 for DMSO versus DMSO + NH_4_Cl. **e**, Representative images and quantification of photoreceptors in autophagy-competent or autophagy-null *rho*:eGFP–tau-WT fish. Autophagy abrogation accelerated the loss of rod photoreceptors, whereas 3 μM methocarbamol rescued retinal degeneration in both *atg7*^+/+^ and Atg7-deficient (*atg7*^−/−^) fish to the same extent; scale bar, 50 μm (*n* ≥ 39 eyes per condition; data are shown as mean ± s.d.). Data were analyzed by one-way ANOVA, ^####^*P* ≤ 0.0001, followed by Tukey’s multiple comparisons test with the following significance values: ****P ≤ 0.0001 versus DMSO (–) and ^ɸɸɸɸ^*P* ≤ 0.0001 versus *atg7*^+/+^. Source data

Based on our previous results showing that CA inhibition reduced the levels of total, hyperphosphorylated and aggregated tau in Dendra–tau-A152T fish, we assessed if CA inhibition enhanced tau protein clearance. Tau clearance rate was evaluated by imaging photoconverted red Dendra signal in living neurons in the spinal cord of fish with mosaic expression of Dendra–tau, as described previously^17,18^. Methocarbamol (3 μM) increased the clearance rate of wild-type (tau-WT) and mutant tau forms (tau-A152T and tau-P301L; Fig. 3a). Similar results were found when using 1 μM methazolamide or following the knockdown of *ca4a* using CRISPR–Cas9 (Fig. 3b). These results demonstrate that chemical and genetic inhibition of CAs accelerate tau clearance.

**Extended Data Fig. 5:**
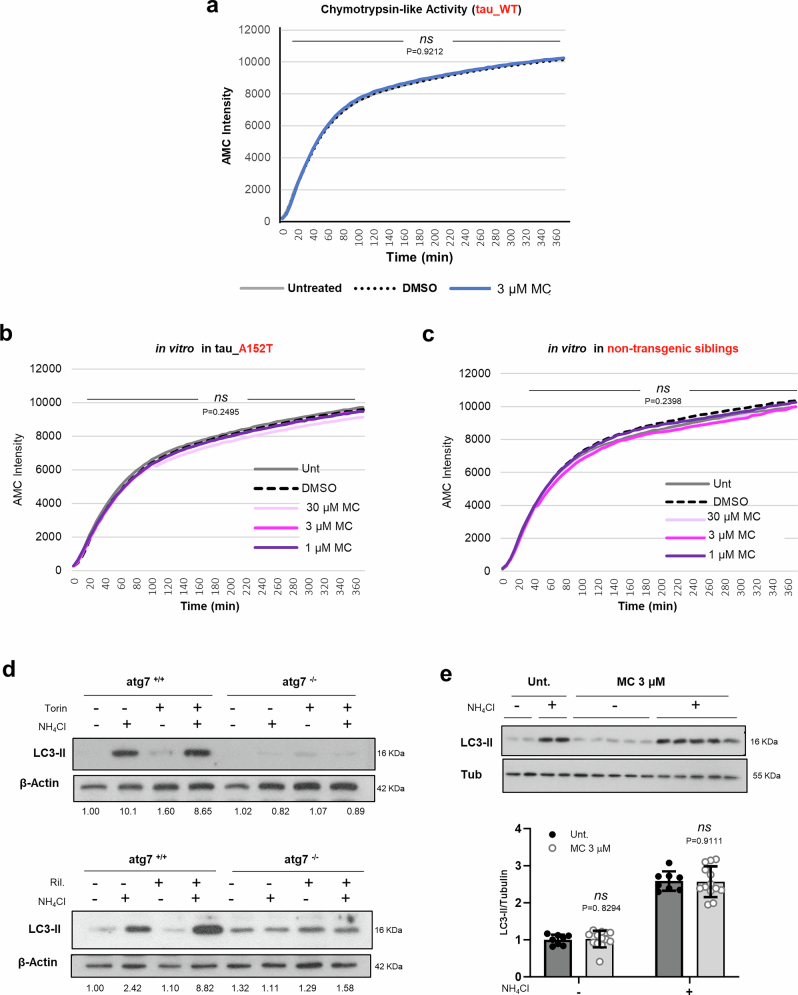
Carbonic anhydrase modulation does not affect proteasomal or lysosomal degradation. **a**. Proteasomal chymotrypsin-like activity using the synthetic Suc-LLVY-AMC substrate in lysates from fish with pan-neuronal expression of Dendra-tau-WT treated with 3 μM methocarbamol (MC) for 24 hours. Methocarbamol treatment did not affect the chymotrypsin-like activity compared to untreated or DMSO-treated siblings (n = 10 fish from 3 independent clutches per group in triplicate; graph represents mean ± SEM; *ns p*≥ 0.05 vs. DMSO analysed by two-way ANOVA). **b** & **c**) Concentration-response effect of methocarbamol (MC) in the proteasomal chymotrypsin-like activity when added to homogenates from larvae with pan-neuronal expression of Dendra-tau-A152T (**b**) or non-transgenic siblings (**c**). Methocarbamol treatment did not affect the chymotrypsin-like activity at any concentration compared to untreated or DMSO-treated homogenates (n = 20 fish from 3 independent clutches per group in triplicate; graph represents mean ± SEM; *ns p*≥ 0.05 vs. DMSO analysed by two-way ANOVA*)*. **d**) Western blot validation of the atg7^−/−^ autophagy-null line. Treatment with the autophagy upregulators rilmenidine 50 μM (Ril.) and torin 1 μM, or the lysosomal inhibitor NH_4_Cl (4 hour-treatment) increased LC3-II levels in *rho*:eGFP–tau-WT on a wild-type (atg7^+/+^) background but did not increase LC3-II in autophagy null, *rho*:eGFP–tau-WT;atg7^−/−^ larvae. ß-actin used as loading control. Relative change of LC3-II normalized to atg7^+/+^ untreated is annotated below ß-actin. **e**. Images and quantification of LC3-II levels by western blot showing no differences upon 3 μM methocarbamol (MC) treatment in wild-type fish in the presence or absence of lysosomal inhibitor NH_4_Cl. Tubulin was used as loading control. (n = 8 DMSO and n = 13 methocarbamol groups; graph represents mean ± SEM; *ns p≥0.05 vs. DMSO* analysed by two-tailed unpaired t-test). Source data

To determine whether CA inhibition affects tau proteasomal degradation, fish with mosaic Dendra–tau-WT expression were treated with 3 μM methocarbamol alone or in combination with the proteasome inhibitor MG132. Treatment with methocarbamol increased the clearance rate of tau-WT, whereas 10 μM MG132 slowed clearance. However, when methocarbamol was used in combination with MG132, the increased clearance rate was sustained compared with treatment with MG132 alone (Fig. 3c), indicating that methocarbamol increases tau-WT clearance independent of its proteasomal degradation. Moreover, methocarbamol still induces tau-A152T clearance in the Dendra–tau-A152T model, which has impaired proteasome activity^17^ (Fig. 3a). Additionally, the proteasomal chymotrypsin-like enzymatic activity in fish with pan-neuronal expression of Dendra–tau-WT showed no changes after 24 h of treatment with 3 μM methocarbamol (Extended Data Fig. 5a). No changes were found in chymotrypsin-like activity when adding 1 to 30 μM methocarbamol in vitro to homogenates from fish expressing Dendra–tau-A152T or negative siblings (Extended Data Fig. 5b,c), suggesting that the mechanism by which CA inhibition increases tau clearance is not mediated by effects on proteasomal protein degradation.

Next, we investigated lysosomal-dependent tau clearance by adding ammonium chloride (NH_4_Cl), which abrogates lysosomal acidification and degradative capacity (Fig. 3d). Blockade of lysosomal degradation with 10 mM NH_4_Cl slowed tau clearance in both DMSO- and methocarbamol-treated fish. However, treatment with the CA inhibitor still facilitated tau clearance even in the presence of NH_4_Cl.

To determine whether CA inhibitor-induced tau clearance effects were mediated by autophagy, the *rho*:eGFP–tau-WT transgenic line was crossed with an autophagy-null mutant line. First, the *atg7*^−/−^ (autophagy-null) line was validated to confirm that there was no autophagy induction in response to known autophagy upregulators (Extended Data Fig. 5d). Next, photoreceptor degeneration was quantified in the offspring of *rho*:eGFP–tau-WT; *atg7*^+/−^ × *atg7*^+/−^ fish treated with either DMSO or 3 μM methocarbamol. Methocarbamol treatment rescued tau-induced photoreceptor degeneration in both autophagy-competent (*atg7*^+/+^) and autophagy-null (*atg7*^−/−^*)* fish (Fig. 3e). Moreover, this rescue in *atg7*^−/−^ fish occurred to a similar extent as autophagy-competent siblings (23.7% versus 26.2%, respectively; Fig. 3e). In addition, 3 μM methocarbamol did not induce any change in levels of LC3-II (an autophagosome marker whose abundance correlates with autophagosome abundance) in wild-type fish in the presence or absence of the lysosomal inhibitor, NH_4_Cl, suggesting that there were no changes in autophagic flux (Extended Data Fig. 5e). These results suggest that methocarbamol effects are autophagy independent and that the decrease of toxic tau species following CA inhibition is not caused to any significant extent by increased proteasomal or lysosomal degradation.

### CA inhibition induces tau secretion by lysosomal exocytosis

Distinct from degradative processes, secretion can ameliorate excessive accumulation of aggregate-prone proteins under proteotoxic stress or of undigested substrates in the case of lysosomal storage disorders. More specifically, it has been shown that clearance of toxic material can be promoted by lysosomal exocytosis^19–22^. Due to the complexity of in vivo models for investigating protein release to the extracellular matrix, we studied this potential mechanism using cell-based experiments.

**Extended Data Fig. 6:**
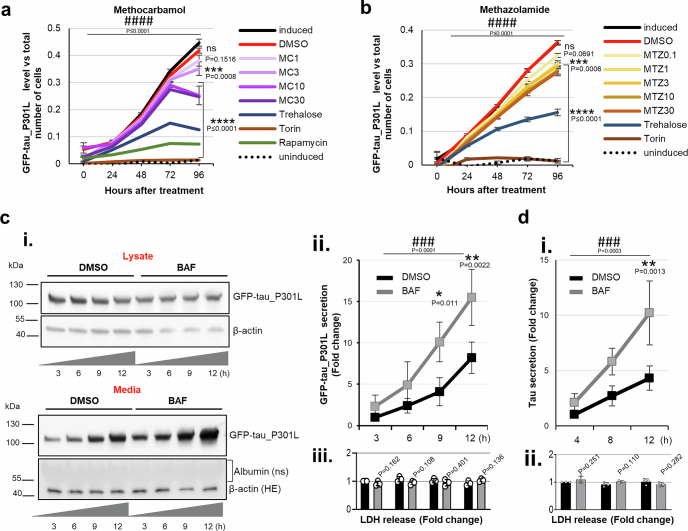
Carbonic anhydrase inhibition increases tau clearance by promoting tau secretion. **a** & **b**) Concentration-dependent decrease of GFP-tau_P301L accumulation when inducible SH-SY5Y cells expressing tau_P301L were treated with methocarbamol (MC) (1, 3, 10, 30 μM) (**a**), or methazolamide (MTZ) (0.1, 1, 3, 10, 30 μM) (**b**); positive controls (0.4 μM rapamycin, 1 μM torin, 100 mM trehalose). No tau detected in uninduced condition. Data are mean± SEM GFP values/cell numbers (Cell Tracker Red). Values were normalised to mean of DMSO-treatment in each independent experiment at each time point (n = 3 independent experiments for MC and n = 4 independent experiments for MTZ; ***p≤ 0.001 and ****p≤ 0.001 vs. DMSO; two-way ANOVA followed by Dunnett’s multiple comparisons test, ^####^p≤ 0.0001). To simplify graph, significance against DMSO were only annotated for final time point. **c**) GFP-trap pull-down experiment showing increased extracellular tau in cell media after treatment with 100 μM Bafilomycin A1 (BAF) versus DMSO-treated SH-SY5Y cells expressing GFP-tau_P301L after 24-hour induction with 0.2 µg/ml doxycycline. (i) Immunoblots showing tau levels in cell lysates and immunoprecipitated tau from media collected over 12 hours at 3-hour intervals. ß-actin was loading control for lysates and control for potential differential cell death/lysis in cell medium (ii) Quantification of tau secretion rate. Values normalized to DMSO at t = 3 h (mean of n = 3 experiments ± SD; *p≤0.05 and **p≤0.01 vs. DMSO; by two-way ANOVA followed by Dunnett’s multiple comparisons test, ^###^p≤ 0.001). (iii) Quantification of LDH release in media at each time point (mean of n = 3 experiments ± SD*; ns for p>0.05* vs. DMSO; two-tailed Student’s t test). **d**) Split luciferase assay in stable SH-SY5Y cells expressing HiBit-tagged tau incubated in complete medium with DMSO or 100 μM Bafilomycin A1 (BAF) over 12 hours and collected at 4-hourly intervals. BAF treatment (i) without affecting LDH release (ii). Values normalized to DMSO at t = 4 h (mean of n = 3 experiments ± SD; *p≤0.05 and **p≤0.01 vs. DMSO; two-way ANOVA followed by Dunnett’s multiple comparisons test, ^###^p≤ 0.001). Bars indicate LDH release (mean of n = 3 experiments ± SD; *ns for p>0.05 vs*. DMSO; two-tailed Student’s t test). Source data

Using SH-SY5Y neuroblastoma cells with doxycycline-inducible expression of GFP–tau-P301L (SH-Tau cells), we measured the levels of tau under control conditions or with varying concentrations of methocarbamol or methazolamide. We observed a concentration-dependent effect of methocarbamol and methazolamide in slowing the expected increase in tau levels (Extended Data Fig. 6a,b). The slower accumulation of intracellular GFP–tau-P301L after drug administration (seen following treatment with methocarbamol, methazolamide or autophagy upregulators such as 1 μM torin, 0.4 μM rapamycin or 100 mM trehalose) reflects changes in intracellular GFP–tau-P301L protein clearance (Extended Data Fig. 6a,b). These results suggest that CA inhibition stimulates tau clearance in cell culture, similar to the effects observed in zebrafish models.

**Fig. 4:**
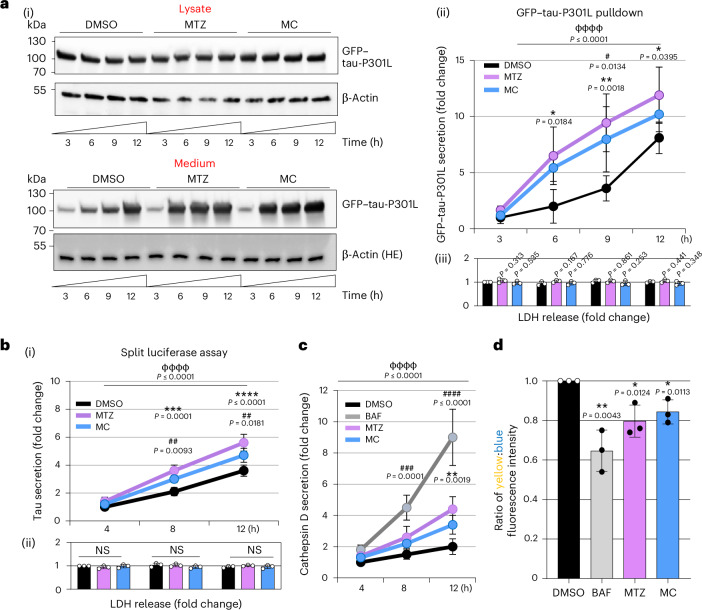
CA inhibition induces the secretion of tau via lysosomal exocytosis. **a**, GFP-Trap pulldown of extracellular tau from the medium of SH-SY5Y cells expressing GFP–tau-P301L after 24-h induction with 0.2 µg ml^–1^ doxycycline. Treatment with 30 μM methazolamide or 30 μM methocarbamol increased extracellular tau compared with medium from DMSO-treated cells. Immunoblots (i) show tau levels in cell lysates and immunoprecipitated tau from medium over 12 h (β-actin was used as the loading control for lysates). The relative quantification of the tau secretion rate (ii) is shown on the right. Values were normalized to DMSO at *t* = 3 h (data are shown as the mean of *n* = 3 biological replicates ± s.d.); *P ≤ 0.05 for methazolamide versus DMSO; *P ≤ 0.05 and **P ≤ 0.01 for methazolamide versus DMSO; ^*#*^*P* ≤ 0.05 for methocarbamol versus DMSO. Data were analyzed by two-way ANOVA followed by Tukey’s multiple comparisons test (^ɸɸɸɸ^*P* ≤ 0.0001). Quantification of LDH release was monitored from each medium fraction (iii; data are shown as the mean of *n* = 3 biological replicates ± s.d.). Data were analyzed by two-tailed Student’s *t*-test. **b**, Split luciferase complementation assay in stable SH-SY5Y cells expressing HiBit-tagged tau incubated in complete medium in the presence of DMSO, 30 μM methazolamide or 30 μM methocarbamol over 12 h. Drug treatment increased extracellular tau compared with treatment with DMSO (i) without affecting LDH release (ii). Values were normalized to the control sample (DMSO) at *t* = 4 h (i; data are shown as the mean of *n* = 3 biological replicates ± s.d.). Data were analyzed by two-way ANOVA (^ɸɸɸɸ^*P* ≤ 0.0001), followed by Tukey’s multiple comparisons test, ***P ≤ 0.001 and ****P ≤ 0.0001 for methazolamide versus DMSO, ^##^*P* ≤ 0.01 for methocarbamol versus DMSO. For ii, the bars indicate LDH release (data are shown as the mean of *n* = 3 biological replicates ± s.d.). Data were analyzed by two-tailed Student’s *t*-test. **c**, Levels of cathepsin D in cell medium determined by ELISA after incubation of SH-SY5Y cells in complete medium with DMSO, 100 μM bafilomycin A1 (BAF), 30 μM methazolamide or 30 μM methocarbamol over 12 h. Values were normalized to DMSO at *t* = 4 h (data are shown as the mean of *n* = 3 biological replicates ± s.d.); **P ≤ 0.01 for methazolamide versus DMSO; ^###^*P* ≤ 0.001 and ^####^*P* ≤ 0.0001 for bafilomycin A1 versus DMSO. Data were analyzed by two-way ANOVA followed by Tukey’s multiple comparisons test (^ɸɸɸɸ^*P* ≤ 0.0001). **d**, Lysosomal pH was analyzed in SH-SY5Y cells following incubation in complete medium with DMSO, 100 μM bafilomycin A1, 30 μM methazolamide or 30 μM methocarbamol for 6 h using LysoSensor DND-160. A reduced yellow-to-blue ratio is indicative of increased pH. Values were normalized to DMSO (data are shown as the mean of *n* = 3 biological replicates ± s.d.); *P ≤ 0.05 and **P ≤ 0.01 versus DMSO. Data were analyzed by two-tailed Student’s *t*-test; Ctl, control. Source data

A time-course series of GFP-Trap tau pulldown experiments in these cells revealed that 30 μM methocarbamol and 30 μM methazolamide caused a greater time-dependent increase in GFP–tau-P301L levels in the culture medium than treatment with DMSO (Fig. 4a(i),(ii)). The increase in extracellular tau is not caused by the escape of intracellular tau as a result of increased cell death, as lactate dehydrogenase (LDH) release remains unchanged compared with DMSO-treated control cells (Fig. 4a(iii)). Similar results were found when using a split luciferase complementation assay in a monoclonal stable SH-SY5Y cell line expressing HiBit-tagged tau (Fig. 4b(i),(ii)). To test whether tau release can be mediated by the secretion of lysosomal contents, we first assessed the effect of methazolamide and methocarbamol on cathepsin D secretion, the most abundant lysosomal protease^23^. SH-SY5Y cells incubated in complete medium with 30 μM methazolamide or 30 μM methocarbamol showed a time-dependent increase in cathepsin D levels in the cell medium, as determined by enzyme-linked immunosorbent assay (ELISA; Fig. 4c). Treatment with 100 μM bafilomycin A1, a well-known lysosomal acidification blocker, also showed this effect but to a greater extent. The concomitant increase of extracellular tau with the lysosome intraluminal protease cathepsin D in the growth medium suggests that lysosomal secretion may contribute to its presence in cell medium.

Alkalinization of the lysosomal lumen has been previously associated with increased lysosomal exocytosis^24,25^. Because CAs are known to be involved in cellular pH regulation, we hypothesized that the effects of CA inhibition on tau secretion may be mediated by changes in lysosomal pH. Treatment with 100 μM bafilomycin A1 promoted an increase in the secretion rate of tau in cells expressing GFP–tau-P301L (Extended Data Fig. 6c) and HiBit-tagged tau (Extended Data Fig. 6d). Treatment of SH-SY5Y cells with LysoSensor DND-160 in combination with 100 μM bafilomycin A1 resulted in a decreased yellow-to-blue ratio, indicative of less acidic organelles (Fig. 4d). Treatment with 30 μM methocarbamol or 30 μM methazolamide had similar effects (although to a lesser extent; Fig. 4d), suggesting that CA modulation can affect the pH of acidic compartments, mostly lysosomes.

**Extended Data Fig. 7:**
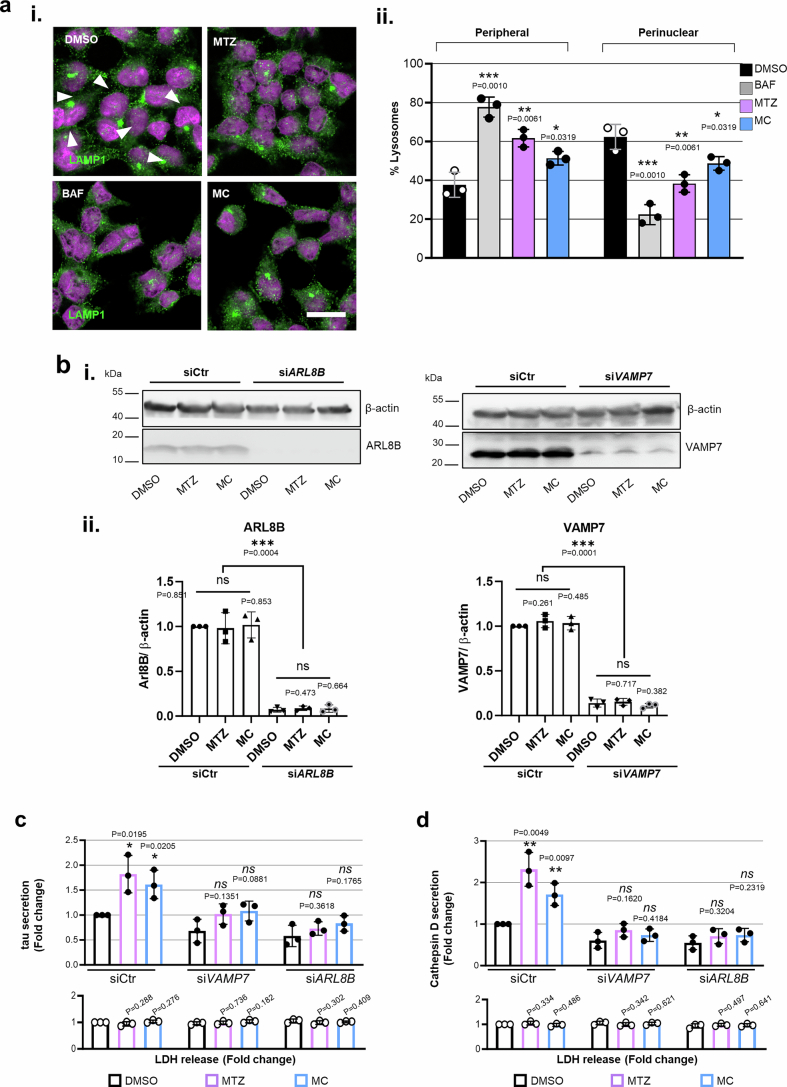
Carbonic anhydrase inhibition causes redistribution of lysosomes and tau secretion by lysosomal exocytosis. **a**) Images and quantification of lysosomal re-distribution from the perinuclear region to the periphery in SH-SY5H cells upon treatment with DMSO, 100 μM Bafilomycin A1 (BAF), 30 μM methazolamide (MTZ) or 30 μM methocarbamol (MC) for 6 h. (i) Representative confocal images showing immunofluorescence staining of lysosomes (LAMP1 in green) and nuclei stained with DAPI (magenta). Arrowheads point to the perinuclear localization of lysosomes in the DMSO group. Scale bar: 50 µm). (ii) Quantification of LAMP1 distribution using CellProfiler Analyst. (n = 3 independent experiments with a minimum of 40 cells/condition for each; graph represents mean ± SD; *p≤0.05, **p≤ 0.01, ***p≤ 0.001 vs. DMSO analysed by two-tailed Student’s t test). **b**) Knockdown efficiency of siRNA-targeted genes VAMP7 and Arl8B in SH-SY5Y cells expressing HiBit-tagged tau incubated in complete medium in presence of DMSO, 30 μM MTZ or 30 μM MC for 12 hours. (i) Western blots and their relative quantification (ii) showing reduced VAMP7 and Arl8B levels after transfection with indicated siRNAs. ß-actin was used as loading control for lysates. Values were normalized to control sample (siRNA ctr + DMSO) (mean of n = 3 experiments ± SD; ***p≤0.001 analysed by two-tailed Student’s t test). **c**) Quantification of tau secretion by the split luciferase complementation assay in SH-SY5Y cell line expressing HiBit-tagged tau transfected with siRNAs targeting *VAMP7* and *ARL8B*. Abrogation of lysosomal exocytosis by siRNAs reduces drug-dependent tau secretion to the media without affecting cell integrity (LDH release). **d**) Quantification of cathepsin D release by ELISA in SH-SY5Y cells transfected with siRNAs targeting *VAMP7* and *ARL8B*. Drug effect on cathepsin D secretion is lost after preventing lysosomal exocytosis. For c and d, cells were incubated in complete medium with DMSO, 30 μM MTZ or 30 μM MC for 12 hours. Values were normalized to DMSO (mean of n = 3 experiments ± SD; *p≤0.05, **p≤0.01 vs. DMSO analysed by two-tailed Student’s t test). Bars in the bottom graph indicate LDH release (mean of n = 3 biological replicates ± SD; *ns for p>0.05 vs. DMSO* analysed by two-tailed Student’s t test). Source data

**Extended Data Fig. 8:**
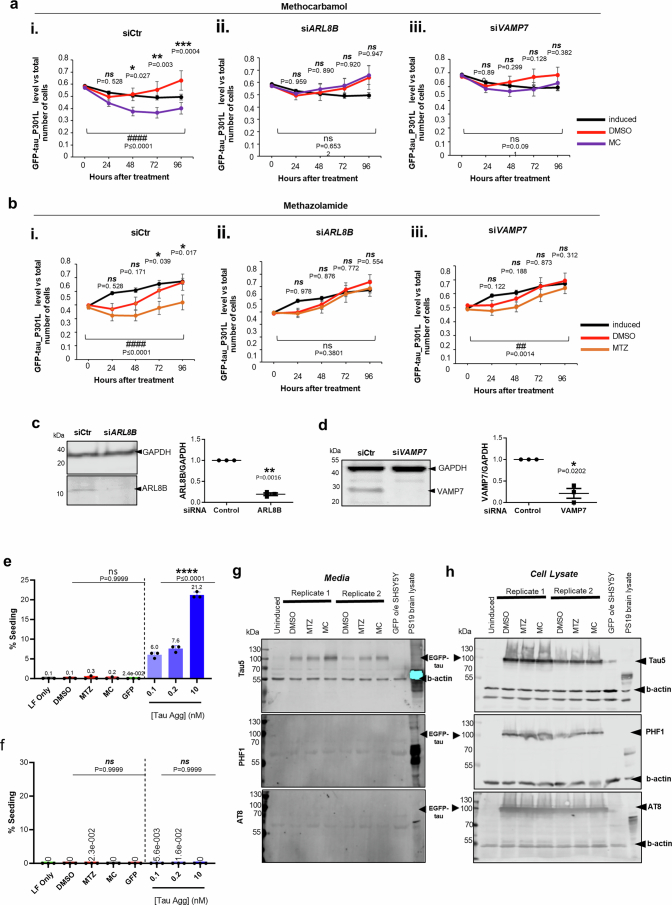
The increase in tau secretion caused by CA inhibition is abrogated by ARL8BB and VAMP7 depletion and secreted tau is unable to seed. **a** & **b**) Graphs showing the intracellular levels of GFP-tau_P301L upon transfection with siRNA-targeted genes Arl8B, VAMP7 or control scramble after carbonic anhydrase inhibition with methocarbamol (**a**) or methazolamide (**b**). Levels of intracellular tau were only significantly reduced in control cells, whereas abrogation of lysosomal secretion by silencing of Arl8B or VAMP7 blocked this effect. Data shown as mean corrected values of GFP *vs* total number of cells ± SEM. Values were normalised to mean of DMSO-treated of each independent experiment at each time point (n = 3 independent experiments; *p≤0.05, **p≤ 0.01, ***p≤ 0.001 vs DSMO analysed by Dunnet’s multiple comparisons two-way ANOVA, ^##^p≤ 0.01, ^####^p≤ 0.0001). **c** & **d**) Images and quantification of western blots for the knockdown efficiency of siRNA-targeted genes Arl8B (**c**) and VAMP7 (**d**) in GFP-tau_P301L cells, showing reduced Arl8B and VAMP7 levels after transfection. GAPDH was used as loading control for lysates. Values were normalized to control sample (siRNA ctr + DMSO) (mean of n = 3 ± SEM; *p≤0.05 and **p≤0.01 analysed by two-tailed one-sample t-test). **e** & **f**) Graphs showing the seeding ability of secreted tau in the media from inducible SH- Tau P301L cells treated with methocarbamol (MC) and methazolamide (MTZ) in comparison with recombinant tau seeding capacity in HEK293 cells expressing tau P301S Venus. Lipofectamine only (LF only) and SH-SY5Y cells transfected with empty GFP vector (GFP) were used as negative control. No seeding of secreted tau was observed, despite 0.1–0.2 nM recombinant tau being able to seed (**e**). As a negative control of the seeding, the entire experiment each time was run in parallel in the parental cell line, HEK293 cells not expressing tau P301S Venus, resulting in no seeding (**f**). Data shown as mean ± SEM of 3 independent experiments. ****p≤ 0.0001 vs DMSO, analysed by one-way ANOVA followed by Dunnet’s comparison test. **g** & **h**) Representative images of the secreted tau (media) and cellular tau (cell lysates), immune blotted with Tau5, PHF1 and AT8 antibodies, from 3 above seeding experiments in e and f, to show detection by Tau5, but not phospho-tau AT8 and PHF1 in secreted tau. This experiment was repeated 3 times. Only one representative blot for each antibody is shown. Source data

Lysosomes mainly localize to the perinuclear region, but it has recently been demonstrated that peripheral lysosomes are less acidic than juxtanuclear lysosomes^26^. Treatment with 30 μM methocarbamol, 30 μM methazolamide or 100 μM bafilomycin reduced the proportion of perinuclear lysosomes and increased lysosomes in the peripheral region compared with DMSO-treated cells (Extended Data Fig. 7a(i),(ii)). Furthermore, short interfering RNA (siRNA) silencing of *VAMP7* or *ARL8B*, essential genes for lysosomal exocytosis^27,28^, abolished the increase in tau secretion induced by 30 μM methocarbamol or 30 μM methazolamide (Extended Data Fig. 7b,c). In addition, the drug-induced increase in extracellular cathepsin D was abrogated by *VAMP7* or *ARL8B* inhibition (Extended Data Fig. 7d) as well as the decrease in intracellular tau levels (Extended Data Fig. 8a–d), confirming inhibition of lysosomal exocytosis and suggesting that the release of tau to the cell medium indeed relies on this process.

To test the seeding competency of secreted tau, we performed a well-established cell-based assay^29^ using HEK293 cells expressing tau-P301S–Venus. We used concentrated medium with 0.1–0.2 nM secreted tau measured by ELISA for these experiments and used 0.1 and 0.2 nM recombinant tau-P301S seed as positive controls for comparison. No seeding was observed in HEK293 cells (both parental cells and cells expressing tau-P301S–Venus) when using eGFP–tau secreted in the medium by SH-Tau cells, whereas 0.1–0.2 nM recombinant tau was able to seed in HEK293 cells expressing tau-P301S–Venus (Extended Data Fig. 8e,f). Western blotting using antibodies to AT8 and PHF1 (detecting phosphorylated tau) detected no signals for secreted tau in the medium, whereas total tau could be seen, as assessed by western blotting using antibody to Tau5 (Extended Data Fig. 8g,h). These results suggest that secreted tau is not phosphorylated and is not able to seed.

Together these results suggest that CA inhibition promotes the alkalinization of acidic compartments, including lysosomes, which in turn, relocalize to the periphery of the cell. Here, they fuse with the plasma membrane, promoting lysosomal exocytosis and secretion of nonseeding competent tau and other intralysosomal components, such as cathepsin D, into the extracellular medium.

### Methazolamide pharmacokinetics and CA activity in wild-type mice

To investigate the potential therapeutic use of CA inhibitors in the treatment of tauopathies, we next examined the effects of CA inhibition in mice using methazolamide because it has higher blood–brain barrier (BBB) penetrance than methocarbamol (0.81 versus 0.57 BBB penetration probability).

**Extended Data Fig. 9:**
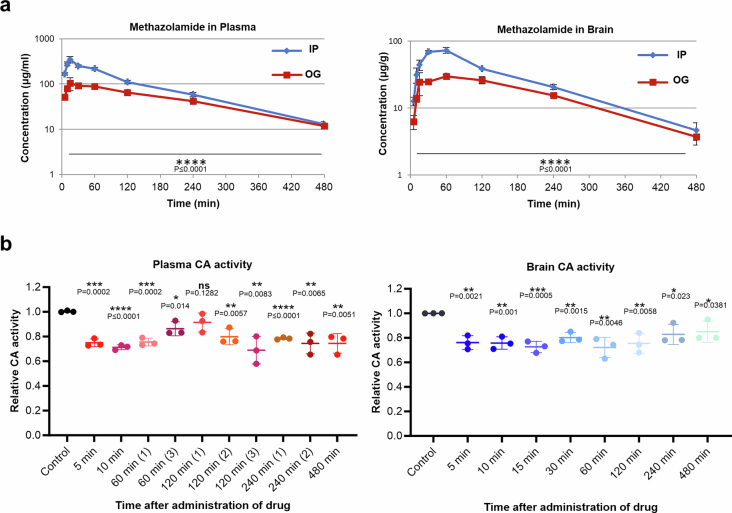
Pharmacokinetics of methazolamide and carbonic anhydrase activity modulation following a single dose of 200 mg/Kg in C57Bl/6 mice. **a**) Chart presentation of the time-course of blood and brain concentrations of methazolamide (MTZ) following a single dose of 200 mg/Kg in C57/Bl6 3-4-month-old wild-type mice after intraperitoneal (IP) or oral gavage administration (OG). Methazolamide concentration rapidly increases in blood and brain, reaching maximum concentrations 15 minutes after administration, and progressively decreases over the duration of the analysis (final time point 480 minutes). Intraperitoneal injection leads to higher methazolamide concentrations in both tissues compared to when the drug is administered by oral gavage. (n = 3 mice/group shown as mean ± SEM). **b**) Time-dependent effect of methazolamide (MTZ) on carbonic anhydrase (CA) activity in plasma and brain samples from wild-type mice treated with a single dose of 200 mg/Kg via intraperitoneal. CA activity was measured by a colorimetric enzymatic assay. Methazolamide has a prolonged effect reducing CA activity in blood and brain of wild-type mice from 5 minutes after administration. (Plasma: n = 1 sample in triplicates shown as mean ± SD corresponding to a mouse number in brackets as (1) =mouse 1, (2) =mouse 2 and (3) =mouse 3; Brain: n = 3 mice/group measured in duplicates shown as mean ± SD; *p≤ 0.05, **p≤ 0.01, ***p≤ 0.001 vs. Control analysed by two-tailed unpaired t-test). Source data

First, we studied methazolamide pharmacokinetics in 3- to 4-month-old wild-type C57BL/6J mice after a single dose of 200 mg per kg (body weight) administered by intraperitoneal (i.p.) injection or oral gavage (OG; Extended Data Fig. 9a and Supplementary Table 2). Injection of methazolamide i.p. resulted in a higher maximum concentration (*C*_max_) in plasma and brain than an equal dose administered by OG (348 μg ml^–1^ versus 105 μg ml^–1^ in plasma and 72.9 μg g^–1^ versus 30 μg g^–1^ in the brain). However, the *C*_max_ was reached at the same time in both tissues independent of the route of administration (*t*_max_ of 15 min in plasma and 60 min in the brain; Extended Data Fig. 9a and Supplementary Table 2). The half-life (*t*_1/2_) was higher after OG administration (151 after OG administration versus 102 min after i.p. administration in plasma and 136 after OG administration versus 112 min after i.p. administration in the brain), but levels normalized to similar values after 8 h (Supplementary Table 2). Of note, no signs of toxicity were seen in any of the treated mice. These results suggest that (1) methazolamide is safe at doses as high as 200 mg per kg (body weight), (2) methazolamide crosses the mouse BBB, and (3) methazolamide accumulates in the brain for a minimum of 8 h.

Second, we evaluated the effects of methazolamide administered i.p. on CA activity in plasma and brain samples by enzymatic assay. Relative CA activity was calculated based on the basal CA activity observed in control mice administered vehicle only. Methazolamide treatment inhibited CA activity in mouse plasma 5 min after i.p. injection (in keeping with the detection of the drug in plasma; Extended Data Fig. 9a,b), and this activity remained decreased 8 h after drug administration (Extended Data Fig. 9a,b). Similar results were observed in mouse brains (Extended Data Fig. 9a,b). No apparent correlation was found between drug concentration and enzymatic inhibition. However, these results suggest that methazolamide can reduce CA activity very rapidly in mouse brains and that this effect can be maintained for at least 8 h despite the progressive decrease in drug concentration.

### Methazolamide reduces CA activity and tau levels in Tg4510 mice

**Fig. 5:**
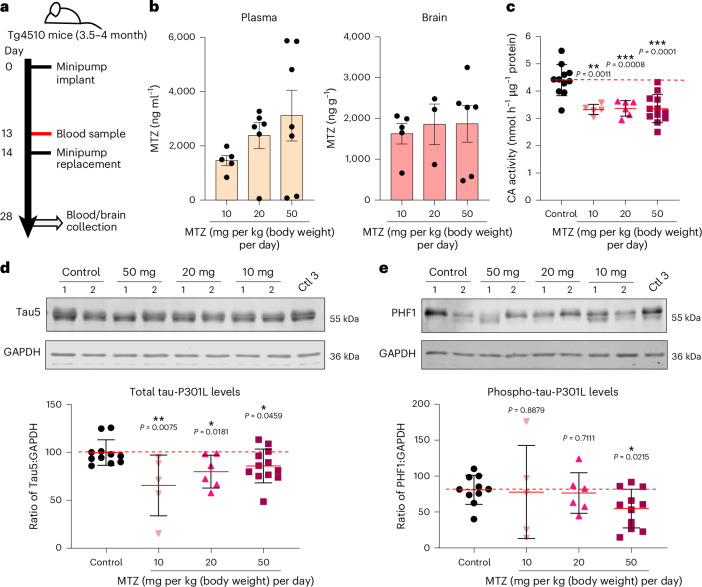
CA inhibition reduces total and phospho-tau levels in Tg4510 tau transgenic mice. **a**, Subcutaneous methazolamide-loaded osmotic minipumps (ALZET Model 2002) were implanted in 3.5- to 4-month-old Tg4510 mice (both sexes) for 28 days. Pumps were replaced once at day 14. To determine the human equivalent dose, three different doses of methazolamide (10, 20 and 50 mg per kg (body weight) per day) were used. At day 13, a blood sample was collected before implanting the second minipump. Terminal blood and brain samples were collected at day 28 (*n* = 5 for 10 mg, *n* = 6 for 20 mg, *n* = 12 for 50 mg and *n* = 11 for vehicle control mice). **b**, Graph representing the concentration of methazolamide in the plasma (ng ml^–1^) and brains (ng g^−1^) of Tg4510 transgenic mice treated with 10, 20 and 50 mg per kg (body weight) methazolamide for 28 days by subcutaneous osmotic minipumps (*n* = 5 mice per group (minimum); data are shown as mean ± s.e.m.). **c**, Effect of treatment with 10, 20 or 50 mg per kg (body weight) per day methazolamide on brain CA activity as determined by colorimetric enzymatic assay. Methazolamide reduced CA activity at all doses and had a prolonged effect, reducing CA activity in the brains of wild-type mice from 5 min after administration (*n* ≥ 5 mice per group measured in duplicates; data are shown as mean ± s.d.); **P ≤ 0.01 and ***P ≤ 0.001 versus control. Data were analyzed by two-tailed unpaired *t*-test. **d**,**e**, Representative images and quantification of western blots to evaluate the effects of treatment with 10, 20 or 50 mg per kg (body weight) per day methazolamide on the levels of total tau (Tau5; **d**) and phosphorylated tau (PHF1; **e**) in the brains of Tg4510 mice. GAPDH was used as a loading control. Methazolamide reduces total and hyperphosphorylated tau levels compared with control treatment (*n* ≥ 5 mice per group; data are shown as mean ± s.e.m.); *P ≤ 0.05 and **P ≤ 0.01 versus control. Data were analyzed by two-tailed unpaired *t*-test. Source data

Based on published data, a single oral dose of methazolamide from 1.5 to 100 mg administered to healthy humans can reach a plasma *C*_max_ between 2.5 and 11.1 μg ml^–1^ depending on the clinical study^30^. To achieve methazolamide plasma concentrations similar to those described in humans, we implanted osmotic minipumps subcutaneously in 3.5- to 4-month-old Tg4510 transgenic mice expressing mutant tau-P301L^1^, dosing the drug at 10, 20 or 50 mg per kg (body weight) per day for 28 days according to scheme in Fig. 5a.

We found a dose-dependent increase in methazolamide plasma concentration (Fig. 5b and Supplementary Table 3). Drug administration at 20 and 50 mg per kg (body weight) per day resulted in plasma concentrations close to the lowest *C*_max_ found in humans. Despite drug concentrations in the brain being ~40-fold lower than the brain *C*_max_ concentrations after a single i.p. dose of 200 mg per kg (body weight) in wild-type mice (Fig. 5b and Extended Data Fig. 9a), these low concentrations were equally efficient in decreasing CA activity (Fig. 5c).

Methazolamide treatment reduced total tau-P301L levels under all conditions compared with control treatment (osmotic minipumps releasing vehicle only; Fig. 5d). Moreover, analysis of PHF1 by western blotting showed that 50 mg per kg (body weight) per day methazolamide treatment also reduced levels of hyperphosphorylated tau in mouse brains (Fig. 5e). These results suggest that methazolamide administered at doses resulting in equivalent plasma concentrations to those found in humans inhibits CA activity and reduces total tau levels (at all concentrations) and hyperphosphorylated tau levels (at 50 mg per kg (body weight) per day) in Tg4510 mice.

### Methazolamide reduces tau and improves cognition in PS19 mice

**Fig. 6:**
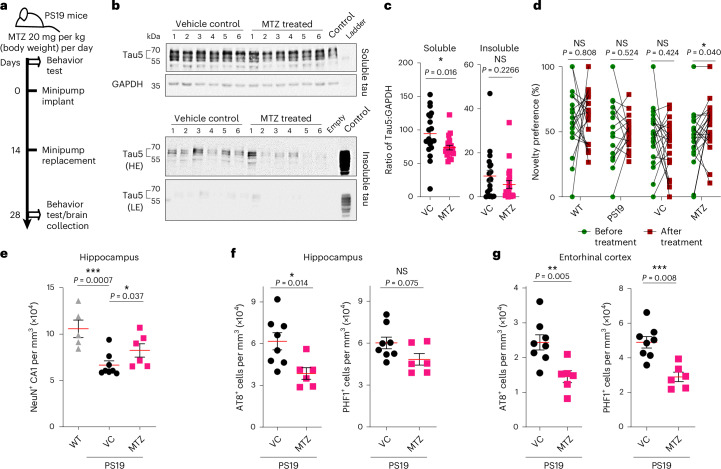
Methazolamide treatment reduces tau levels and neuron loss and improves object recognition in PS19 tau transgenic mice. **a**, Scheme for methazolamide dosing in PS19 mice. Tau PS19 mice at 8–9 months were implanted with subcutaneous methazolamide-loaded osmotic minipumps (20 mg per kg (body weight) per day; ALZET Model 2002) for 28 days in total. Pumps were replaced once at day 14. Behavioral testing was performed once before implanting the minipump and subsequently at days 26 (habituation to arena) and 27 (novel object recognition testing) after treatment. **b**,**c**, Representative images (**b**) and quantification (**c**) of western blots to evaluate the effects of 20 mg per kg (body weight) per day methazolamide treatment on levels of tau (Tau5) in sarkosyl-soluble and sarkosyl-insoluble fractions from the cerebral cortex of PS19 mice compared with mice treated with vehicle only. The same soluble fraction from a vehicle control-treated mouse was run in parallel to insoluble fractions on every gel (**b**); HE, high exposure; LE, low exposure. GAPDH was used as a loading control. Methazolamide treatment significantly reduced the levels of soluble tau (soluble Tau5/GAPDH) compared with vehicle control treatment and diminished insoluble tau (insoluble Tau5/GAPDH) to a lesser extent (**c**). Insoluble fractions were normalized to control (*n* = 21 methazolamide-treated mice and *n* = 19 vehicle control-treated mice). Values are shown as mean ± s.e.m.; *P ≤ 0.05 versus vehicle control. Data were analyzed by two-tailed unpaired *t*-test; VC, vehicle control. **d**, A novel object recognition task was performed using PS19 mice (untreated) and wild-type littermates (untreated) in parallel to PS19 mice treated with methazolamide or vehicle control. All mice were assessed at 34 weeks and were reassessed at day 27. Wild-type and PS19 littermates that were not implanted with minipumps were assessed in parallel (*n* = 20 wild-type mice; *n* = 15 PS19 mice; *n* = 22 PS19 mice treated with methazolamide; *n* = 21 vehicle control-treated PS19 mice). The plot shows changes in scores from 34 weeks to 34 weeks + 27 days (after treatment) as the percentage of novelty preference analyzed using a Wilcoxon matched-pairs signed-rank test (nonparametric method) to compare before and after treatment novel object recognition task scores; *P ≤ 0.05 versus vehicle control. **e**, Unbiased stereological estimates of numbers of NeuN^+^ CA1 neurons in the hippocampus of PS19 mice and vehicle-treated and methazolamide-treated PS19 mice. Wild-type littermate brains were used as a positive control. Data in the plots represent mean ± s.e.m. (*n* = 8 vehicle-treated mice and *n* = 6 methazolamide-treated mice per group). Data were analyzed by one-tailed unpaired *t*-test. **f**,**g**, Effects of 20 mg per kg (body weight) per day methazolamide treatment on levels of phosphorylated tau, as detected by antibodies to AT8 and PHF1 in the hippocampus (CA1) and entorhinal cortex regions of brains of PS19 mice. Methazolamide treatment reduces hyperphosphorylated tau levels compared with vehicle control treatment. Data in the plots represent mean ± s.e.m. (*n* = 8 vehicle-treated mice and *n* = 6 methazolamide-treated mice per group). Data were analyzed by two-tailed unpaired *t*-test. Source data

We next investigated the effect of CA inhibition on PS19 transgenic mice carrying the P301S tau mutation. These mice progressively accumulate tau in aggregated forms from 6 months of age^31,32^ onward and show more progressive neurodegenerative disease than Tg4510 mice. Subcutaneous osmotic minipumps were implanted in 8- to 9-month-old (for behavioral and biochemical analyses) and 9- to 10-month-old (for immunohistochemistry) PS19 mice, delivering a methazolamide dose of 20 mg per kg (body weight) per day, and these minipumps were replaced after 14 days (Fig. 6a). This protocol was selected as the lowest dose in mice able to reach equivalent plasma concentrations as those described in humans based on our studies using Tg4510 mice.

Sarkosyl-tau preparations from the brains of mice treated with methazolamide for 28 days showed reduced levels of sarkosyl-soluble tau and a nonsignificant trend toward lowered sarkosyl-insoluble tau compared with vehicle-treated mice (Fig. 6b,c).

**Extended Data Fig. 10:**
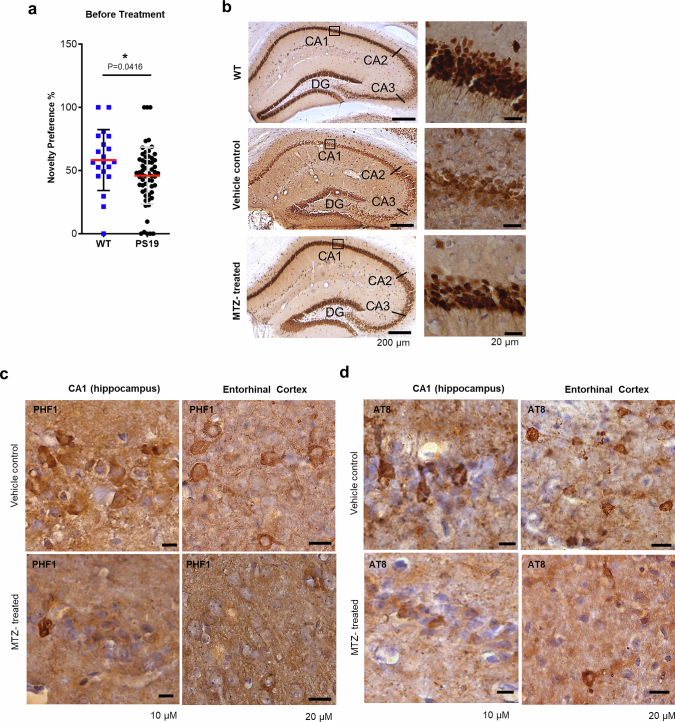
Methazolamide treatment reduces the tau levels in PS19 tau transgenic mice. **a**) PS19 mice (PS19) and their wild-type littermates (WT) were tested for novel object recognition (NORT) task at 34 weeks of age before treatment. PS19 showed reduced novelty preference (%). Two-tailed un-paired Student’s t-test, exact p values are shown. *p≤ 0.05 vs.WT mice (n= 20 WT mice and 59 PS19 mice). **b**) Representative images of brain (hippocampus) sections at low magnification (4X) and at higher magnification (63X), showing amelioration of hippocampal (CA1) neuronal loss after treatment with MTZ (20 mg/Kg/day) administered with osmotic minipump after NeuN immunostaining. Scale bar 200 μm and 20 μm for low and higher magnification images, respectively. **c** & **d**) Representative microscopic images used for the quantification in Fig. 6g & h to show the effects of 20 mg/Kg/day methazolamide (MTZ) administered via osmotic minipump on the levels of phosphorylated-tau, detected by PHF-1 (**c**) and AT8 (**d**) immunostaining in hippocampus (CA1) and entorhinal cortex (EC) regions of brains of PS19 mice. Methazolamide reduces hyperphosphorylated tau levels compared to vehicle control mice. Scale bar 10 µm for hippocampus (CA1) and 20 µm for entorhinal cortex (EC). Source data

At the age of 34 weeks (8 months), when PS19 mice showed a reduced novelty preference in the novel object recognition task (Extended Data Fig. 10a), methazolamide administered through subcutaneous minipumps for 28 days (from 8 to 9 months old) improved cognitive function, as assessed by the novel object recognition task, when we compared pre- and post-treatment values for mice in the methazolamide treatment group, which was not seen in the vehicle control treatment group (Fig. 6d).

Nine- to 10-month-old PS19 mice were chosen to study neuron loss in the hippocampus and entorhinal cortex^32^. Methazolamide treatment rescued CA1 neuron loss (Fig. 6e and Extended Data Fig. 10b) and reduced the levels of phosphorylated tau detected by immunohistochemistry for modified tau AT8 and PHF1 (Fig. 6f,g and Extended Data Fig. 10c,d).

## Discussion

Our data demonstrate that CA inhibition in zebrafish increases tau clearance in living neurons, and this effect is independent of the proteasomal and autophagic degradation pathways. Our cell studies in the SH-SY5Y neuronal line expressing mutant tau-P301L revealed that the CA inhibitors methocarbamol and methazolamide reduce intracellular tau by increasing its secretion to the extracellular medium. Tau has been shown to be secreted by exosomes, plasma membrane-derived vesicles, late endosomal secretory pathways, direct translocation across the plasma membrane and other unconventional secretion processes (which do not require signal sequences in the target protein)^33–36^. The concomitant increase of the lysosomal enzyme cathepsin D in the cell medium and the redistribution of lysosomes to the cell periphery after CA inhibition suggests that tau is secreted through lysosomal exocytosis, a pathway previously associated with cellular clearance^21,37,38^. The genetic abrogation of this process by silencing key molecular components of this mechanism, such as *VAMP7* and *ARL8B*^27,39,40^, impaired tau and cathepsin D secretion to the culture medium and reduced intracellular tau levels induced by CA inhibition. Moreover, changes in the lysosomal redistribution induced by CA inhibition in cell culture were accompanied by changes in the lysosomal pH, previously shown to promote lysosomal exocytosis^25,41^.

CAs, that have a wide distribution inside cells and at the plasma membrane, have been extensively studied as intracellular and extracellular pH regulators due to their enzymatic activity catalyzing the reversible conversion of CO_2_ and H_2_O into HCO_3_^−^ and H^+^ (ref. ^42^). In addition, CA protein and its activity were previously described within lysosomes in guinea pigs, human leukocytes and rat liver^43–45^, and the use of CA inhibitors, such as acetazolamide, inhibited the acidification of the lysosomal compartment in vivo, suggesting a potential physiological contribution of CA to the acidification of lysosomes^46^.

Often, compound screens following the repurposing strategy result in drugs showing potential benefits but at a concentration range far from the plasma concentration found after human dosing. However, here we have demonstrated that CA inhibition at concentrations in mice similar to those seen in patients taking prescribed doses classically used for treating musculoskeletal and neuropathic pain and glaucoma^47–50^, has a beneficial effect in the CNS, lowering levels of tau in neurons from mouse brains. The CA inhibitor methazolamide, administered via subcutaneous minipumps, reached similar plasma concentrations as those observed in humans after methazolamide dosing, caused a reduction in hyperphosphorylated and soluble tau in mouse brains and improved behavioral performance.

Tau secretion is believed to be an important component underlying tau spreading between neurons, a phenomenon that is believed by some to have an important contribution to tauopathies. This is thought to be transsynaptic^34,51^, and perhaps the neuronal sites of tau secretion matters. However, some have suggested that ‘distant’ spreading of tau between brain regions is unlikely to occur in Alzheimer’s disease (at least from Braak stage 3 onward where this could be assessed)^52^. Our cell-based data suggest that secreted tau species following CA inhibition are seeding-incompetent and nonphosphorylated, at least within the limitations of the tools and assays at our disposal. In addition, our in vivo results support that CA inhibition acts to stimulate unconventional secretion (at least via lysosomal exocytosis) to lower tau levels and reduce neurodegeneration in zebrafish and mouse tauopathy models. This concept is consistent with observations that the transcription factor TFEB positively regulates lysosomal exocytosis and that loss of this function appears to correlate with increased tau toxicity^19^. Interestingly, the tau species that are secreted via this TFEB-dependent route are not prone to spreading^19^.

Our study also highlights the potential of zebrafish as a useful model species to investigate compound screens targeting neurodegenerative disorders and tauopathies in particular. This small vertebrate recapitulates disease hallmarks found in humans, but the speed at which these pathologies manifest (such as formation of insoluble tau and neurodegeneration) offers the potential for faster, higher-throughput and sensitive screening of modifiers than traditional rodent models^53,54^. Collectively, our experimental plan allowed us to perform an in vivo screen of hundreds of drugs to sequentially identify, validate and characterize new disease modulators using zebrafish, neuronal cell lines and mouse tau models.

Together, our results suggest that CA inhibition ultimately regulates lysosomal acidification and cellular distribution, promoting lysosomal exocytosis and tau secretion. This mechanism lowers tau levels within neurons, which, in turn, have lower levels of hyperphosphorylated and aggregated toxic tau forms, accounting for an improvement in phenotypic, neuronal loss and behavioral defects in vivo in zebrafish and mouse models. This raises the possibility of rapid repurposing of CA inhibitors for tauopathies, as our studies were performed in mice at human-like plasma concentrations. Furthermore, our data suggest that stimulation of unconventional secretion may also be a potent therapeutic approach for other neurodegenerative diseases caused by toxic, aggregate-prone intracellular proteins.

## Methods

### Zebrafish experiments

#### Maintenance of transgenic zebrafish lines

All zebrafish experiments were performed in accordance with the UK Animals (Scientific Procedures) Act with appropriate Home Office Project and Personal animal licenses and with University of Cambridge Animal Welfare and Ethical Review Body approval. Studies were performed in accordance with PREPARE and ARRIVE guidelines. Zebrafish were maintained on a 14-h light/10-h dark cycle under standard conditions^55^.

All transgenic zebrafish lines used in this study are registered on the Zebrafish Information Network (ZFIN) database and are listed as follows. The retinal models used to assess photoreceptor degeneration were the transgenic line Tg(*rho*:EGFP-Hsa.MAPT)cu7, referred here to as *rho*:eGFP–tau-WT and expresses human wild-type tau in rod photoreceptors, and the *rho*:eGFP line Tg2(*rho*:EGFP)cu3, which expresses GFP in rod photoreceptors; both were first described by Moreau and colleagues^14^. The transgenic line expressing mutant tau-P301L is assigned Tg(*rho*:EGFP-Hsa.MAPT_P301L)cu12 on the ZFIN database and is referred to as *rho*:eGFP–tau-P301L here. This line was generated in-house as previously described^13^. The *Gal4* driver line Tg3(Xla.*Eef1a1*:GAL4-VP16)cu11 was generated in-house and is referred to as *EIf1a*:Gal4^14^, and the pan-neuronal Gal4VP16 driver line (referred to as *PanN*:Gal4) was a kind gift from H. Baier, Max Planck Institute for Biological Intelligence, Munich, Germany (identified as s1101tEt in the original publication^56^). Transgenic zebrafish lines expressing Dendra2 fused to either wild-type tau, Tg(UAS:Dendra2-Hsa.MAPT,*myl7*:EGFP)cu9 referred to as Dendra–tau-WT, or mutant tau-A152T, Tg(UAS:Dendra2-Hsa.MAPT_A152T,*myl7*:EGFP)cu10 referred to as Dendra–tau-A152T, were made in-house as previously described^17^. The transgenic line expressing Dendra-tagged tau-P301L was generated in-house as previously described for fish expressing Dendra-tagged tau-A152T^17^. The transgenic line is assigned Tg(UAS:Dendra2-Hsa.MAPT_P301L,*myl7*:EGFP)cu61 on the ZFIN database and is referred to as Dendra–tau-P301L.

The *atg7*^+/−^ (*atg7*^sa14768^) mutant fish line was obtained from the Zebrafish Mutation Project^57^ and was used for generating autophagy-null fish, as previously described^4^.

#### Experimental crosses

Embryos from natural spawnings were collected in embryo medium (5 mM NaCl, 0.17 mM KCl, 0.33 mM CaCl_2_, 0.33 mM Mg_2_SO_4_ and 5 mM HEPES, pH 7.2), staged according to established criteria^58^ and reared at 28.5 °C in the dark in an incubator. Petri dishes containing embryos were cleaned daily, and embryo medium was replenished when needed. Eggs collected from a single breeding tank are termed a clutch.

Crosses of the homozygous *rho*:eGFP–tau-WT line to Tupfel Longfin (TL) wild-type zebrafish or incrosses of the transgenic line were used to generate heterozygous or homozygous offspring for primary screening and validation experiments, respectively. Offspring from crosses of the *rho*:eGFP–tau-P301L line to wild-type TL zebrafish were used for further investigation of methocarbamol. The embryos from these *rho* promoter lines were treated with 0.003% phenylthiourea (PTU) in embryo medium from 1 d.p.f. to block pigment formation and screened for eGFP fluorescence in the eyes at 4 d.p.f. on a Leica M205 FA fluorescence microscope. Fish were then washed in embryo medium to remove PTU. In clutches from crosses of homozygous *rho*:eGFP–tau-WT fish, all offspring express the transgene, and, hence, no PTU was required.

Crosses of the Dendra–tau lines to *PanN*:Gal4 resulted in fish with pan-neuronal expression of Dendra–tau transgenes throughout the CNS. These were used to confirm drug activity and to evaluate the effects of pharmacological and genetic modulation of CAs on the phenotypic abnormalities and biochemical defects previously described in these lines^17^. Crosses of the Dendra–tau lines with the *Eif1a*:Gal4 driver line resulted in mosaic expression of Dendra–tau in all tissues and were used to measure tau clearance kinetics in individual neurons in the spinal cord^18^.

Crosses of *rho*:eGFP–tau-WT fish to *atg7*^+/−^ fish were used to generate *rho*:eGFP–tau-WT; *atg7*^+/−^ fish. Crosses of *rho*:eGFP–tau-WT; *atg7*^+/−^ fish to *atg7*^+/−^ fish resulted in a proportion of *rho*:eGFP–tau-WT; *atg7*^−/−^ offspring unable to perform autophagic degradation.

#### Primary screen

##### Treatments with compound library

A panel of 1,437 drugs from the JHCCL^15^ were tested in the zebrafish model *rho*:eGFP–tau-WT to identify new potential tau toxicity modulators.

The compound library was provided in a 96-well-plate format. Compounds were solubilized in either DMSO or water at a final concentration of 100 mM and stored at −80 °C. On the day before the start of drug treatment, a daughter plate was prepared at a concentration of 10 mM in the appropriate solvent. The daughter plates contained sufficient quantities to complete the 5-day treatment and were stored in the dark at room temperature in a desiccator chamber. Working dilutions were identified by codes without any correspondence to its name or clinical indication.

For the primary screen, offspring from crosses of the homozygous *rho*:eGFP–tau-WT line to wild-type TL zebrafish were collected, cleaned, counted and kept in the incubator until 6 d.p.f. Larvae from a single clutch were then arrayed in a 24-well plate with ten larvae per well, with each well containing 1 ml of embryo medium. One microliter of compound (10 mM) was added to each well, resulting in a final concentration of 10 μM in the well. For each clutch, one plate included vehicle control wells (water and 0.1% DMSO) and one well treated with 10 μM clonidine as a positive control. Larvae were treated from 6 to 10 d.p.f. with 0.5 ml of embryo medium and 0.5 µl of compound replenished daily. Fish health and motility were monitored throughout the treatment period, and signs of toxicity or swimming impairment were noted; larvae were culled immediately if toxicity was observed. At 10 d.p.f., fish were culled by an overdose of 1,3-amino-benzoic acid ethylester (MS222), and samples were collected, dried and stored at −80 °C for subsequent western blotting.

For validation of the results from the primary screen, new commercial stocks were purchased for the selected compounds and tested over a range of concentrations in homozygous larvae from incrosses of homozygous *rho*:eGFP–tau-WT fish because these presented with more severe retinal degeneration. Four replicates were included for these analyses.

##### Western blotting analysis of rod photoreceptor degeneration

Quantification of rod photoreceptors in fish samples from the primary screen and validation experiments was performed by measuring the amount of endogenous rod-specific rhodopsin protein (zpr-3, ZIRC; 1:250; 36 kDa) compared with the cone-specific protein arrestin-3 (zpr-1, ZIRC; 1:500; 40 kDa) by western blotting, as previously described^14^.

##### Imaging of rod photoreceptor degeneration

Quantification of rod degeneration in *rho*:eGFP–tau lines was performed on images from 10-μm cryosections across the central retina of larvae fixed at 10 d.p.f., as previously described^4^. A minimum of 24 eyes were quantified for these experiments, and data are represented as photoreceptor area in pixels.

##### eGFP–tau expression in *rho*:eGFP–tau-WT fish

eGFP–tau expression in drug-treated *rho*:eGFP–tau-WT fish was evaluated using RNA isolated from pools of ten larvae at 10 d.p.f. extracted using an RNeasy Plus Mini kit (Qiagen) following the manufacturer’s instructions. Fish treated with 0.1% DMSO or 3 μM methocarbamol were compared with untreated siblings. A total of 50 ng of RNA from each condition was used in One-Step qPCR combining cDNA synthesis and real-time PCR. We used customized TaqMan gene-specific primers for *EGFP* and *GAPDH* as loading controls and followed the manufacturer’s instructions (Invitrogen; *GAPDH* TaqMan made-to-order gene expression code 4351372 Dr03436845_g1 and *EGFP* TaqMan made-to-order code 6302625 from Applied Biosystems). Three independent clutches were evaluated in triplicate and analyzed on a StepOne Plus Real Time PCR System using StepOne Software V.2.1 (Applied Biosystems, Life Technologies). Relative gene expression was normalized to that of *GAPDH* and calculated using the $2^{-{\Delta}{\Delta}{C}_{t}}$ method.

##### Immunostaining to detect phosphorylated tau in the retinal model

Cryosections across the central retina of *rho*:eGFP–tau-WT and *rho*:eGFP–tau-P301L fish at 10 d.p.f. were used to investigate the effects of 1 µM methazolamide on phosphorylated tau. Antibody staining for the tau marker AT8 (Ser 202–Thr 205) was performed as previously described^14^. Images of eGFP signal (green) and AT8-specific signal (red) from a minimum of 26 eyes per group were acquired with a Zeiss Axio Zoom.V16 microscope with a QImaging Retiga 2000 R digital camera and QCapture Pro 7.0 software. Images were merged using Fiji software (ImageJ 1.54f) to show the proportion of rods with AT8^+^ staining. The number of green (GFP^+^) and red (anti-AT8^+^) rods was quantified manually in both images and is represented as the percentage of rods showing AT8 staining (see representative images in Extended Data Fig. 3e(ii)).

#### Phenotypic assessment in Dendra–tau models

Embryos with pan-neuronal expression of Dendra–tau-A152T develop abnormal morphological phenotypes, as previously described^17^. Expression of both tau-A152T and tau-P301L transgenes results in a proportion of fish with bent bodies in ‘S’ and ‘C’ shapes. Phenotypes were scored as being normal or having mild, moderate or severe morphological defects by using a dissecting microscope. The quantification of the number of fish in each category was performed at 3 d.p.f. This analysis has been used previously to assess the effect of pharmacological and genetic modulators of tau toxicity^4,13,17^.

Phenotypic assessment following drug treatment was performed by adding compounds at the indicated concentrations to the embryo medium at 1 d.p.f. Drug concentration was maintained in embryo medium by replenishing the drug and medium daily until 3 d.p.f., when phenotypes were scored blind to limit biased interpretation of the treatment. Drugs were tested at the following concentrations; methocarbamol at 3, 10 and 30 μM and methazolamide, acetazolamide and tioxolone at 0.1, 0.3, 1, 3, 10, 30 and 100 μM. Fish were monitored daily, and any adverse effects were noted.

#### Western blotting

Samples for western blotting were prepared and processed as previously described^17^. The primary antibodies used were Tau5 mouse anti-tau (1:1,000; 80579, Abcam), mouse anti-tubulin (1:5,000; T6199, Sigma-Aldrich), mouse anti-PHF1 (tau phosphorylated at Ser 396–Ser 404 used at 1:100; a kind gift from P. Davies, Albert Einstein College of Medicine of Yeshiva University), rabbit anti-LC3 (1:1,000; NB100-2220, Novus Biologicals), rabbit anti-CA IX (H-11) for Ca9 (1:500; sc-365900, Santa Cruz), rabbit anti-CA IV (G-11) for Ca4 (1:500; sc-74527, Santa Cruz) and anti-β-actin (1:1,000; A5316, Sigma-Aldrich). The secondary antibodies used were goat anti-mouse horseradish peroxidase (HRP; 1:5,000; P044701-2, Agilent) and goat anti-rabbit HRP (1:5,000; P044801-2, Agilent).

Immunoreactive bands were detected by the addition of enhanced chemiluminescence (ECL) substrate (GE Healthcare Bioscience) using Hyperfilm ECL (Amersham) and a Fujifilm FPM-100A developer, except for images of sarkosyl-soluble and sarkosyl-insoluble tau in Fig. 1f,g, which were acquired using an LI-COR Odyssey Fc and Image Studio software (version 5.2). Images were digitized, and densitometry of the bands was quantified using ImageJ (Fiji) software.

#### Fractionation of soluble and insoluble tau

Pools of 50 embryos with pan-neuronal expression of Dendra–tau-A152T were collected at 6 d.p.f. after being treated with 0.1% DMSO or 3 μM methocarbamol from 1 d.p.f. and frozen at −80 °C. The extraction of soluble and sarkosyl-insoluble tau fractions was performed using the standard protocol^1^ with some modifications for fish samples, as previously described^17^.

#### Tissue-specific CA gene expression

Eye-specific expression of different zebrafish CAs was evaluated by extracting RNA using a Norgen Single Cell RNA Purification kit (51800) according to the manufacturer’s protocols from ten whole bodies or pools of 40 eyes from *rho*:eGFP–tau-WT fish at 9 d.p.f. cDNA was synthesized from 1 μg of RNA using an AB Biosynthesis cDNA kit (4368814, Applied Biosystems) according to manufacturer’s instructions. cDNA was then used to perform PCR with a temperature gradient (ranking from 55 to 65 °C) for the annealing step to determine the optimal annealing conditions for each primer pair for each CA isoform. Primers were designed for the genes encoding the most abundant cytosolic isoforms Ca2a and Cahz; membrane-bound isoforms Ca4a, Ca4b, Ca4c, Ca9 and Ca14 and the mitochondrial ortholog Ca5 (see Supplementary Table 4). PCR products were then resolved on agarose gels to visualize bands at the specific expected sizes.

Expression of CAs in Dendra–tau^+^ neurons from fish with pan-neuronal expression was investigated by isolation of Dendra^+^ cells by FACS. Fish were culled at 3 d.p.f. by overdose of anesthesia and collected into tubes (50 fish per tube). After yolk sac removal by pipetting up and down several times in 500 μl of deyolking solution (55 mM NaCl, 1.8 mM KCl and 1.25 mM NaHCO_3_), tissue was disaggregated in 900 μl of liberase solution (0.075 mg ml^–1^ Roche Liberase Research Grade in DEPC-PBS) by mechanical disruption, pipetting up and down every 2–3 min for no longer than 20 min to avoid inducing cell death at room temperature. Then, 100 μl of fetal bovine serum (FBS; F7524, Sigma-Aldrich) was added, and tubes were placed on ice to stop the reaction. The dissociated cell suspension was then filtered through a 30-μm cell strainer (Filter Cell Trics 30 μm, Sysmex, 04-0042326) into a 15-ml tube on ice. The filter was then washed with 10 ml of cold DEPC-PBS, and the eluate was collected in the same tube. Cells were pelleted by centrifugation at 600*g* for 3 min at 4 °C and resuspended in 500 μl of cold DEPC-PBS by gently pipetting. Dendra^+^ neurons were then sorted using a FAC-SORTER MoFlo Astrios.

RNA was extracted from a minimum of 150,000 cells using a Norgen Single Cell RNA Purification kit (51800) and reverse transcribed to cDNA using an AB Biosynthesis cDNA kit according to manufacturer’s protocols. Analysis of CA expression by PCR was performed as described above for whole bodies and eyes.

#### CRISPR injections

A total of four CRISPR guide RNAs were custom designed for each eye and/or CNS-expressed CA isoform by Horizon Discovery (Dharmacon; listed in Supplementary Table 5). A mixture of all four CRISPR guide RNAs was used to maximize the silencing of each gene^59^. One hundred nanograms of each CRISPR guide RNA was mixed with 800 ng of transactivating CRISPR RNA (tracrRNA; Dharmacon Edit-R CRISPR–Cas9 synthetic tracrRNA; U002005, Dharmacon) in a total volume of 9.6 µl and incubated at room temperature for 10 min. After incubation, 2.64 µl of 2 M KCl was added, and the final volume was aliquoted into 1.5-µl aliquots and stored at −80 °C until use.

On the day of injection, an aliquot of CRISPR + tracrRNA was thawed on ice and mixed with 5 µg of Cas9 Nuclease Protein NLS (Horizon Discovery, CAS12206). After 5 min of incubation at 37 °C, 0.2 µl of phenol red was added to the solution.

Embryos from homozygous *rho*:eGFP–tau-WT fish crossed with TL wild-type fish or Dendra–tau-A152T fish crossed with the *PanN*:Gal4 line were collected immediately after spawning and injected into the yolk with 4.28 nl of CRISPR–Cas9 solution while at the one-cell stage. Developing embryos were thoroughly cleaned a few hours after injection and monitored daily.

##### Knockdown efficiency by CRISPR injection

The efficiency of CA-targeting CRISPR guide RNAs was evaluated by qPCR. CRISPR injections were performed in five independent clutches of wild-type embryos as described above. Uninjected fish from each clutch were collected for comparison. At 4 d.p.f., pools of ten fish from each group were collected, and RNA was extracted using an RNeasy Plus Mini kit (Qiagen) according to manufacturer’s instructions. A total of 1 μg of RNA from each condition was used to generate cDNA using an AB Biosynthesis cDNA kit (4368814, Applied Biosystems) following the manufacturer’s instructions. cDNA was then mixed with SYBR Green qPCR Master Mix (Thermo Fisher Scientific, 330501) and the relevant CA primer pairs targeted to the C-terminal exons (listed in Supplementary Table 6). qPCR was performed in a Roche LightCycler 480 machine using LightCycler 480 Software (V1.5.1.62). Gene expression values were normalized to *rbs11* as a loading control and calculated using the ${2}^{-{\Delta}{\Delta}{C}_{t}}$ method.

#### Analysis of Dendra–tau clearance kinetics

The clearance rate of tau following drug treatment was determined as previously described^17,18^. Offspring from Dendra–tau-WT, Dendra–tau-A152T or Dendra–tau-P301L fish crossed to the *EIF1a*:Gal4 line have mosaic expression of the transgenes and were used to measure Dendra-tagged tau in individual neurons in the spinal cord. To investigate changes in Dendra–tau clearance kinetics after CA inhibition, 3 μM methocarbamol or 1 μM methazolamide was added to embryo medium after photoconversion, whereas the control group was treated with 0.1% DMSO. To study the influence of lysosomal and proteasomal degradation in Dendra–tau clearance, fish were treated with 3 μM methocarbamol in the presence or absence of 10 mM NH_4_Cl or 100 mM MG132, respectively. Drugs were replenished every 12 h.

The effect of genetic inhibition of CAs on the Dendra–tau-A152T clearance rate was evaluated in fish injected with CRISPR guide RNAs targeting Ca4a (*ca4a*) at the one-cell stage in the presence or absence of 1 μM methazolamide added immediately after photoconversion and compared with their respective uninjected control siblings.

#### Proteasome activity assay

The chymotrypsin-like activity of the proteasome was evaluated in fish homogenates as previously described^17^ with the following modifications. Fish with pan-neuronal expression of Dendra–tau-WT were treated with 3 μM methocarbamol or 0.1% DMSO for 24 h from 1 to 2 d.p.f. Pools of ten fish from three independent clutches were processed as previously described, and 40 μg of protein from each sample was used for the chymotrypsin-like activity assay measuring fluorescence intensity (excitation 355 nm; emission 460 nm) every 5 min over 3 h at 28 °C in a FLUORstar Omega fluorometer (BMG Labtech) with Omega software (V6.20). All samples were measured in triplicate.

‘In vitro’ analysis of the effect of methocarbamol on proteasomal activity was performed on homogenates from fish with pan-neuronal expression of Dendra–tau-A152T and nonexpressing siblings at 2 d.p.f. following the same protocol described above. Once 40 μg of protein was plated and before the addition of 100 mM Suc-LLVY-AMC substrate, methocarbamol was added to the well at final concentrations of 1, 3 or 30 μM. Changes in fluorescence intensity were monitored as described above.

### Cell-based experiments

Cell cultures were maintained under normoxic conditions (5% CO_2_) at 37 °C with regular PCR evaluation to ensure that all cell lines were free from mycoplasma contamination.

#### Analysis of tau clearance in cell culture

Inducible SH-Tau cells (eGFP–tau-P301L)^60^ were maintained in DMEM/F12 medium (D6421, Sigma-Aldrich) supplemented with 10% FBS (F7524, Sigma-Aldrich), 2× nonessential amino acids (M7145, Sigma-Aldrich), 100 U ml^–1^ penicillin–streptomycin (P0781, Sigma-Aldrich) and 2 mM l-glutamine (G7513, Sigma-Aldrich). Cells were incubated in a humidified incubator at 37 °C and 5% CO_2_. For clearance assays, with or without transfection of siRNAs targeting *VAMP7* and *ARL8B*, cells were cultured on poly-d-lysine-coated (50 mg ml^–1^; P6407, Sigma-Aldrich) 96-well, black-walled, flat-bottom μClear plates (655986, Greiner Bio-One) up to a confluency of 70–80%.

##### siRNA transfection

For clearance assays with siRNA transfection, cells were first transfected with *VAMP7* and *ARL8B* siRNA, followed by drug treatment. Cells were reverse transfected with specific individual siRNAs targeting *VAMP7* and *ARL8B* (sequence details are provided in ‘Cell transfection with siRNA’) in 96-well plates (as described above) with siRNA (30 nM final concentration per well) in growth medium without antibiotics. After 4–6 h, the transfection medium was replaced with normal growth medium. After 24 h, cells were split 1:3 using trypsin (T3924, Sigma-Aldrich). The following day, cells were forward transfected with 30 nM siRNA as described before. After 24 h, cells were split 1:2 in 96-well plates and incubated at 37 °C for another 24 h.

##### Drug treatment (with and without siRNA transfection)

After 24 h, cells were washed once with serum-free medium (DMEM/F12 without supplements). After washing, cells were loaded with 1.2 µM Cell Tracker Red CMTPX (C34552, Molecular Probes, Invitrogen) in serum-free medium (DMEM/F12 without supplements) for 30 min at 37 °C. Cell Tracker Red was used as an internal control for the number of cells in the well. After incubation, the cells were washed once with serum-free medium (DMEM/F12 without supplements). Cells were reverse treated (that is, tau expression was switched on and drug treatment was applied at the same time) by the addition of doxycycline (0.2 μg ml^–1^) in combination with methocarbamol at concentrations of 1, 3, 10 or 30 μM or methazolamide at concentrations of 0.1, 1, 3, 10 or 30 μM. Rapamycin (0.4 μM), torin (1 µM) and trehalose (100 mM) were used as positive controls, and cells treated with or without DMSO were vehicle and negative controls, respectively. After 6 h, the plates were scanned by a Tecan Spark Trading microplate reader (SPARK multimode microplate reader with software SPARKCONTROL Method Editor version 3.0) for *t* = 0 for red excitation/emission spectra (577 nm/602 nm) and green excitation/emission spectra (484 nm/535 nm). Plates were subsequently scanned daily for 4 days. Treatments were refreshed after 48 h, and tau expression was switched off by removing doxycycline from the treatment at 48 h. Three independent experiments and four independent experiments were performed for methocarbamol and methazolamide, respectively. Data for tau levels were analyzed as green fluorescence intensity (for eGFP–tau-P301L) corrected for red fluorescence (for Cell Tracker CMTPX, an internal control for the total number of cells) for each plate (replicate). Corrected values were normalized to the mean of three control (DMSO-treated) plates (three replicates) of each independent experiment at each time point. Data were pooled as means of each independent experiment at each time point. Statistical analysis was performed using two-way ANOVAs followed by Dunnett’s multiple comparisons. All analyses were performed in Prism.

#### Analysis of tau secretion

##### Immunoprecipitation of GFP–tau-P301L

Tau secretion was analyzed using the inducible SH-Tau cell line expressing GFP–tau-P301L. Cells from the monoclonal SH-SY5Y cell line expressing GFP–tau-P301L (from Y.-F. Liao’s group Institute of Cellular and Organismic Biology, Taipei, Taiwan) were cultured in DMEM/F12 (D6421, Sigma-Aldrich) supplemented with 10% FBS (F7524, Sigma-Aldrich), 2 mM l-glutamine (G7513, Sigma-Aldrich) and 100 U ml^–1^ penicillin–streptomycin (P0781, Sigma-Aldrich) for 3 days on six-well plates to 50% confluency. Cells were then treated with 0.2 µg ml^–1^ doxycycline for 24 h to induce GFP–tau-P301L expression. After doxycycline induction, cells were washed three times in PBS and incubated in complete medium containing doxycycline (0.2 µg ml^–1^) in the presence of 0.1% DMSO, 100 nm bafilomycin A1, 30 μM methazolamide or 30 μM methocarbamol for 3, 6, 9 or 12 h. At each indicated time (one individual well per time point), 500 μl of culture medium was collected, and cells were lysed in Laemmli sample buffer and boiled for 10 min at 100 °C.

Tau levels in the cell medium were analyzed by immunoprecipitation of GFP–tau-P301L using GFP-Trap magnetic beads (gtma-20, ChromoTek). Culture medium (500 μl) was incubated with 25 μl of prewashed GFP-Trap beads for 1 h at 4 °C on a rotating surface. GFP beads were washed three times with PBS, resuspended with Laemmli sample buffer and boiled for 10 min at 100 °C. Levels of GFP–tau-P301L in the cell lysates were analyzed by western blotting, a LI-COR Odyssey CLX and Image Studio software for western blot imaging. Cell lysates and GFP-Trap immunoprecipitated tau from cell medium were resolved by SDS–PAGE and transferred onto PVDF membranes. PVDF membranes were blocked with 4% skim milk in phosphate-buffered saline with 0.1% Tween 20 (PBST) for 1 h and incubated overnight at 4 °C with the following primary antibodies diluted in 4% skim milk in PBST: rabbit polyclonal anti-GFP (1:1,000; ab6556, Abcam) and rabbit anti-β-actin (1:1,000; A2066, Sigma-Aldrich). After several washes in PBST, membranes were incubated for 90 min at room temperature with an anti-rabbit HRP-linked antibody (1:5,000; 7074, Cell Signaling Technology) diluted in 4% skim milk in PBST. GFP–tau-P301L and β-actin bands were detected using the ECL enhanced chemiluminescence detection kit from GE Healthcare (RPN2106). Experiments were performed in triplicate.

##### Split luciferase complementation assay

Tau secretion was analyzed by a split luciferase complementation assay in cells expressing HiBit-tagged tau. To generate an SH-SY5Y cell line stably expressing HiBit-tagged tau, we transfected SH-SY5Y cells with pcDNA3.1-GFP-2A-HiBit-Tau (810390DE, Thermo Fisher Scientific) using *Trans*IT-2020 transfection reagent (5405, Mirus), following the manufacturer’s recommendations. After 2 days, transfected cells were selected with 600 μg ml^–1^ G-418 (4727878001, Sigma-Aldrich) for 1 week, after which GFP^+^ cells were isolated by FACS, and single cells were collected in 96-well plates. After expansion into six-well format, GFP and HiBit tau expression were assessed from clonal lines by flow cytometry and immunofluorescence microscopy analysis.

Cells from the selected monoclonal stable SH-SY5Y cell line expressing HiBit-tagged tau were incubated in complete medium in the presence of 0.1% DMSO, 100 nm bafilomycin A1, 30 μM methazolamide or 30 μM methocarbamol for 4, 8 or 12 h. The split luciferase complementation assay was performed from 100 μl of cell culture medium using the Nano-Glo HiBit Extracellular Detection System kit, according to the manufacturer’s instructions (N2421, Promega). This protocol is based on the established concept of bimolecular fluorescence complementation and detects extracellular tau luciferase activity only after complementation in the presence of extracellular LgBiT. Values were normalized to control samples treated with DMSO at 4 h. This experiment was performed in triplicate.

#### Cell death analysis

LDH release was monitored from cell culture medium fractions collected at the indicated times to evaluate the levels of plasma membrane damage and cell death using a commercial LDH assay kit (ab65393, Abcam), according to manufacturer’s recommendations. Each experiment was performed in triplicate.

#### ELISA analysis of cathepsin D in cell medium

Levels of cathepsin D released into the cell medium were measured by ELISA. SH-SY5Y cells were incubated in complete medium in the presence of 0.1% DMSO, 100 nM bafilomycin A1, 30 μM methazolamide or 30 μM methocarbamol for the indicated durations. Cathepsin D secretion levels in the cell medium were monitored using a commercial Cathepsin D ELISA kit (ab119586, Abcam) according to manufacturer’s recommendations. Values were normalized to control samples treated with DMSO at 4 h. This experiment was performed in triplicate.

#### Analysis of vesicular pH

SH-SY5Y cells were incubated in complete medium in the presence of 0.1% DMSO, 100 nM bafilomycin A1, 30 μM methazolamide or 30 μM methocarbamol for 6 h, and change in lysosomal pH was analyzed using LysoSensor Yellow/Blue DND-160 (L7545, Thermo Fisher Scientific). After drug treatment, cells were incubated with 2 µM LysoSensor DND-160 for 5 min in the presence of drugs and washed with probe-free medium, and overall cell fluorescence was measured using a SPARK multimode microplate reader with SPARKCONTROL Method Editor software (V 3.0, TECAN Trading). The ratio of blue and yellow intensities was quantified and is represented as fold change compared with control samples treated with DMSO. This experiment was performed in triplicate.

#### Analysis of lysosomal distribution

SH-SY5Y cells (ECACC, 94030304) were cultured on coverslips and incubated in complete medium in the presence of 0.1% DMSO, 100 nM bafilomycin A1, 30 μM methazolamide or 30 μM methocarbamol for 6 h. After treatment, cells were washed with PBS three times and fixed and permeabilized with ice-cold methanol for 4 min. After washing three times with PBS, cells were blocked with 3% bovine serum albumin (BP1605-100, Thermo Fisher Scientific) for 1 h. Cells were incubated with rabbit anti-LAMP1 (1:500; 9091, Cell Signaling Technology) in blocking buffer overnight at 4 °C. After washing three times with PBS, cells were incubated with secondary goat anti-rabbit Alexa Fluor 594 (1:500; A11012, Thermo Fisher Scientific) for 1 h at room temperature. Cells on coverslips were washed with PBS and mounted in ProLong Diamond Antifade Reagent with DAPI (P36962, Thermo Fisher Scientific). Imaging was conducted with an LSM880 Carl Zeiss confocal microscope with a ×63 oil-immersion lens using Leica Application Suite X microscope imaging software. Quantification of the distribution of LAMP1^+^ puncta was performed using CellProfiler Analyst (Broad Institute^61^). Briefly, for all images analyzed, the area of each nucleus and each cell was extracted using the module ‘MeasureObjectSizeShape’. An area of 5 μm width was then defined around the nucleus, allowing for the establishment of perinuclear and peripheral zones in each cell. The LAMP1^+^ signal in each area was assessed to determine whether lysosomes were mainly perinuclear or peripheral. A minimum of 40 cells per condition for each independent experiment were analyzed.

#### Cell transfection with siRNA

Cells of the monoclonal stable SH-SY5Y cell line expressing HiBit-tagged tau and inducible SH-Tau cells (eGFP–tau-P301L) were transfected with specific individual siRNAs targeting *VAMP7* and *ARL8B* using Lipofectamine 2000 transfection reagent (12566014, Thermo Fisher Scientific) following the manufacturer’s recommendations. Individual siRNAs were purchased from Dharmacon. The following siRNA sequences were used: scramble 5′-UGGUUUACAUGUCGACUAA-3′, *ARL8B* 5′-GAUGAGAAACAGCUAAUUG-3′ and *VAMP7* 5′-GUACUCACAUGGCAAUUAU-3′. After 3 days, cells were incubated in complete medium in the presence of 0.1% DMSO, 100 nM bafilomycin A1, 30 μM methazolamide or 30 μM methocarbamol for an additional 12 h.

Knockdown efficiency of targeted genes was monitored by western blotting. Briefly, 3 days after siRNA transfection, cell lysates were resolved by SDS–PAGE and transferred onto PVDF membranes. PVDF membranes were blocked with 4% skim milk in PBST for 1 h and incubated overnight at 4 °C with the following primary antibodies diluted in 4% skim milk PBST: rabbit polyclonal anti-ARL8B (1:1,000; ab207697, Abcam), rabbit polyclonal anti-VAMP7 (1:1,000; SAB2105695, Sigma-Aldrich) and rabbit anti-β-actin (1:1,000; A2066, Sigma-Aldrich). After several washes in PBST, membranes were incubated for 90 min at room temperature with HRP-linked anti-rabbit (7074, Cell Signaling Technology; 1:5,000) in 4% skim milk and 0.1% Tween-PBS. ARL8B, VAMP7 and β-actin bands were detected using the ECL enhanced chemiluminescence detection kit from GE Healthcare (RPN2106).

#### Tau seeding assay

Inducible SH-Tau cells (eGFP–tau-P301L) were cultured in X2 140-mm dishes as described in ‘Analysis of tau clearance in cell culture’. Cells were reverse treated (that is, tau expression was switched on and drug treatment was applied at the same time) by the addition of doxycycline (0.2 μg ml^–1^) in combination with methocarbamol or methazolamide at concentrations of 30 μM and DMSO as a control. Low FBS (1%) and low penicillin–streptomycin (10 U ml^–1^) DMEM/F12 medium was used for reverse treatment. The cells were incubated at 37 °C and 5% CO_2_ for 12 h. After 12 h, the medium was collected and concentrated using Amicon Ultra-15 Centrifugal Filters 10K (UFC901024, Millipore) to a volume of 300 µl; OptiMEM (Thermo Fisher Scientific) was added to increase the volume to 550 µl. Tau levels were determined using a Tau ELISA kit (KHB0042, Invitrogen) according to manufacturer’s instructions.

Seeding assays were performed largely as described previously^29^. HEK293 cells expressing tau-P301S–Venus were plated at 15,000 cells per well in poly-d-lysine-coated black 96-well plates (Corning, 3603) in 50 μl of OptiMEM (Thermo Fisher Scientific). Samples of medium containing released tau–GFP or recombinant tau-P301S assemblies at comparable concentrations were incubated with Lipofectamine 2000 (Invitrogen, 11668019) at a final concentration of 0.5 μl 50 μl^–1^ for 10 min at room temperature. Fifty microliters of the tau-containing medium sample per well was added for 1 h at 37 °C before the addition of 100 μl of complete DMEM per well to stop the transfection process. Cells were incubated at 37 °C for 72 h after DMEM addition. Tau–Venus aggregates were imaged and quantified using a Nikon Ti2 ECLIPSE and high-content imaging analysis software (NIS-Elements AR V. 5.41.02 Nikon) and calculated using the following equation:

$${\mathrm{Percent}}\,{\rm{seeding}}=({\rm{green}}\; {\rm{cells}}\; {\rm{with}}\; {\rm{puncta}}\;/\;{\rm{total}}\; {\rm{green}}\; {\rm{cells}})\times 100.$$

### Mouse experiments

Mouse studies were performed in accordance with the UK Animals (Scientific Procedures) Act with appropriate Home Office Project and Personal animal licenses and with University of Cambridge Animal Welfare and Ethical Review Body approval. Mice were maintained and used in experiments following PREPARE and ARRIVE guidelines.

#### Mice

Mice were housed in individually ventilated cages with free access to standard animal food chow and water in a climate-controlled room (45–65% humidity and 20–24 °C) on a 12-h light/12-h dark cycle. We used two different neurodegenerative disease mouse models. The frontotemporal dementia (Tg4510) mouse model, overexpressing the human tau 0N4R isoform carrying the P301L mutation, as described previously^1,62^, was generated by crossing two transgenic lines. Both congenic parental mouse strains, tetracycline-controlled transactivator (tTA) Tg(*Camk2a*-tTA)1Mmay (007004) and hTau responder FVB-Tg(*tetO*-MAPT*P301L)#Kha/JlwsJ (015815), were purchased from The Jackson Laboratory. Tg4510 mice were bred from these two different parental lines to activate the expression of transgene under the control of a tetracycline conditional gene expression system (tet off, tTA). Transgene expression is largely restricted to the forebrain by the *Camk2a* promoter. Although the mice can be fed with a doxycycline-supplemented diet (R105 with 200 ppm doxycycline, SAFE Diet) to switch off transgene expression, in the current study, we did not repress transgene expression (that is, it remained switched on). The second disease model was the tau (PS19) mouse model, B6N.Cg-Tg(*Prnp*-MAPT*P301S)PS19Vle/J (024841, The Jackson Laboratory). This mouse model expresses the human tau 1N4R isoform with the P301S mutation driven by the mouse prion protein promoter^32^.

#### Methazolamide administration

##### Injection (i.p.) and OG

A stock solution of methazolamide (sc235615, Santa Cruz Biotechnology) was prepared at 200 mg ml^–1^ in DMSO. This was diluted to a final formulation to administer to 3- to 4-month-old wild-type C57BL/6J mice according to body weight with 2% Tween 80 and 30% PEG300 in saline (freshly prepared) on the day. As a control, a separate group of mice was administered vehicle only with 5% DMSO instead of drug with 2% Tween 80 and 30% PEG300 in saline.

##### Subcutaneous administration by osmotic minipump

A solution of methazolamide in DMSO with 30% PEG300 and 2% Tween 80 was given subcutaneously as a continuous infusion with a daily dose of 10, 20 or 50 mg per kg (body weight) by implanting osmotic minipumps (0.5 μl h^–1^ flow rate; Alzet Model 2002) in 3.5- to 4-month-old Tg4510 and 8- to 9-month-old PS19 mice. The concentration of methazolamide in the minipump was adjusted according to each animal’s body weight to obtain the required delivery of 10, 20 or 50 mg per kg (body weight) per day, and the pumps were primed by soaking in saline at 37 °C overnight. Minipumps were replaced after 14 days with freshly loaded minipumps. As a control, a separate group of mice was administered vehicle only (that is, DMSO, 30% PEG300 and 2% Tween 80) via minipump.

#### Pharmacokinetic analysis

Wild-type (C57BL/6J) male mice (3–4 months old) were administered methazolamide (200 mg per kg (body weight)) either by i.p. injection or OG. Mice were anesthetized using isoflurane at eight different time points (that is, 5, 10, 15, 30, 60, 120, 240 and 480 min), with three mice dosed for each time point. Once a mouse was anesthetized, a terminal blood sample was taken by cardiac puncture. Blood (0.3–0.8 ml) was collected in EDTA tubes (Microvette 100K3E, Sarstedt). The plasma fraction was immediately separated by centrifugation (1,000*g*, 5 min, 4 °C) and stored on dry ice and then −80 °C until liquid chromatography–mass spectrometry analysis to determine drug concentration. After confirming death by cervical dislocation, the brains were collected, frozen on dry ice and stored at −80 °C until processing.

Repeat blood sampling (via the saphenous vein) was also performed during the course of continuous dosing of methazolamide via osmotic minipump according to the experiment (at day 13 for Alzet Model 2002), followed by collection of a terminal sample at day 28. At each occasion, blood samples of 100–120 μl were collected in EDTA tubes (Microvette 300K2E, Sarstedt). The plasma fraction was separated and stored as described above.

Drug concentration and pharmacokinetic analyses were performed on plasma and brain tissues using liquid chromatography–mass spectrometry and Noncompartmental Pharmacokinetic Data Analysis software (Windows 2.0.6 Excel 2002 Edition) for pharmacokinetic analyses by Q3 Analytical.

#### CA activity

CA activity was measured by a colorimetric-based assay using a BioVision Carbonic Anhydrase Activity Assay kit (K472-100) according to manufacturer’s instructions with modifications. Twenty to 40 mg of tissue was excised from the cortical region of mouse frozen brains with a scalpel, transferred into a tube and thawed on ice in 1 ml of PBS to clean the tissue. PBS was removed and replaced with 10 μl per mg of tissue of ice-cold 1 mM Tris (pH 8.0) and 200 mM NaCl, and eight glass beads were added to facilitate tissue disruption. After homogenization by sonication (three cycles, 20 s each), samples were centrifuged at 16,900*g* for 15 min at 4 °C. The supernatant was collected and diluted 1:10 in 1 mM Tris (pH 8.0) and 200 mM NaCl for measuring protein concentration by the fluorometric Qubit assay (Qubit, Thermo Fisher Scientific). For plasma samples, plasma was thawed on ice and centrifuged at 3,000*g* for 1 min at 4 °C to remove any cells. Clear plasma was then diluted 1:10 in 1 mM Tris (pH 8.0) and 200 mM NaCl for protein concentration assays. Due to the technical difficulties in collecting enough blood sample for this assay, the enzymatic assay from plasma could not be performed on all three mice for each time point used in the pharmacokinetic study.

To measure CA activity in brain homogenates and plasma, 10 μg of protein in 10 μl was added to each well, with samples analyzed in duplicate. Eighty-five microliters of CA buffer was added to each well (provided in the kit), and the plate was incubated at room temperature for 15 min. A standard curve with increasing concentrations of nitrophenol from 0 to 40 nmol per well (provided in the kit) was run in parallel in duplicate. After incubation, 5 μl of substrate (provided in the kit) was added to each sample well, and absorbance (405 nm) was measured immediately after the addition and every 5 min subsequently for 1 h at room temperature in a Elx800 plate reader (Appleton Woods). CA values were normalized to the activity in untreated/placebo mice.

#### Tau sarkosyl extraction from mouse brain

Brains from PS19 mice underwent soluble and insoluble fractionation using a sarkosyl extraction protocol^1^. Cerebral cortex from mouse brain tissue was homogenized by using a Precellys CK14 Lysing kit (10144-554, Avantor) in TBS buffer (20 mM Tris-HCl (pH 7.4), 150 mM NaCl, 1 mM EGTA and protease inhibitor cocktail). Protein in mouse brain lysates in TBS buffer was quantified by bicinchoninic acid assay. After the addition of Laemmli buffer, samples were boiled for 5–7 min at 100 °C. Western blots for both mouse sarkosyl-soluble and sarkosyl-insoluble fractions and brain lysates were performed on 10% acrylamide SDS–PAGE gels. The same vehicle-treated control mouse sample (for the insoluble fraction) was loaded on each gel for normalization and to allow the comparison between the bands detected on the two different membranes. Blots were blocked in 5% nonfat milk in PBST and incubated with the following primary antibodies overnight: mouse anti-Tau5 (1:1,000; ab80579, Abcam), mouse anti-PHF1 (1:1,000; a kind gift from P. Davies, Albert Einstein College of Medicine, New York, USA) and rabbit anti-GAPDH (1:5,000; NB100-56875, Novus Biologicals). The membranes were labeled with fluorescent secondary antibodies and were analyzed with a LI-COR-Odyssey CLX apparatus. Densitometry analysis on the immunoblots was performed using IMAGE STUDIO Lite software.

#### Novel object recognition test

A novel object recognition test, commonly used to assess changes in short-term memory tasks, was performed as previously described^63^. Behavioral video recording in mice was performed using a Logitech C310 HD Webcam camera connected to Panlab Smart Video Record It Software (Smart 3.0) and analyzed manually using Behavioral Observation Research Interactive Software (BORIS version 8.25.4)^64^.

Statistical analysis on the percentage of time spent on a novel object was performed using a Wilcoxon matched-pairs signed-rank test. All analyses were performed using Prism (GraphPad).

#### Immunohistochemistry of mouse brains

Mouse brain immunostaining and immunohistochemical analysis was performed as described in ref. ^63^.

Stereologic analysis to quantitate neuron loss in the CA1 region in a statistically ‘unbiased’ fashion was performed using previously described techniques^1,63^ and a Zeiss Axio Imager Z2 microscope with Zen Blue software (version 3.3) for imaging immunostained brains. Numbers of neuronal nuclei-positive (NeuN^+^; neuron-specific DNA-binding protein), AT8^+^ and PHF1^+^ cells in the CA1 and entorhinal cortex were estimated and calculated using Stereo Investigator Software. Mean values were calculated for vehicle- and methazolamide-treated groups. Wild-type mouse brains were also quantified as controls for NeuN staining. Data were analyzed by either one- or two-tailed unpaired *t*-tests.

### Statistics

Data are shown as mean ± s.e.m. or s.d. and were calculated with Microsoft Excel. Statistical analysis was performed using two-tailed unpaired Student’s *t*-tests, one-tailed test, one-sample *t*-test, Wilcoxon matched-pairs signed-rank test (nonparametric method), one-way ANOVA followed by Tukey’s multiple comparisons test, Student–Newman–Keuls or Dunnett’s one-way ANOVA or Sidak’s, Dunnett’s or Tukey’s multiple test comparison two-way ANOVA in GraphPad InStat 3. See the figure legends for the specific tests used for each data set. A *P* value of ≤0.05 was considered significant. One-tailed tests were only used when the direction of change could be predicted by previous experiments.

### Reporting summary

Further information on research design is available in the Nature Portfolio Reporting Summary linked to this article.

## Online content

Any methods, additional references, Nature Portfolio reporting summaries, source data, extended data, supplementary information, acknowledgements, peer review information; details of author contributions and competing interests; and statements of data and code availability are available at 10.1038/s41589-024-01762-7.

## Supplementary information

Reporting Summary

Supplementary Table Supplementary Tables 1–6.

## Source data

Source Data Fig. 1 Uncropped scans of blots and gels of western blot data.

Source Data Fig. 1 Raw data for all graphs (separate sheet for each panel).

Source Data Fig. 2 Raw data for all graphs (separate sheet for each panel).

Source Data Fig. 3 Raw data for all graphs (separate sheet for each panel).

Source Data Fig. 4 Uncropped scans of blots and gels of western blot data.

Source Data Fig. 4 Raw data for all graphs (separate sheet for each panel).

Source Data Fig. 5 Uncropped scans of blots and gels of western blot data.

Source Data Fig. 5 Raw data for all graphs (separate sheet for each panel).

Source Data Fig. 6 Uncropped scans of blots and gels of western blot data.

Source Data Fig. 6 Raw data for all graphs (separate sheet for each panel).

Source Data Extended Data Fig. 1 Raw data for all graphs (separate sheet for each panel).

Source Data Extended Data Fig. 2 Uncropped scans of blots and gels of western blot data.

Source Data Extended Data Fig. 2 Raw data for all graphs (separate sheet for each panel).

Source Data Extended Data Fig. 3 Uncropped scans of blots and gels of western blot data.

Source Data Extended Data Fig. 3 Raw data for all graphs (separate sheet for each panel).

Source Data Extended Data Fig. 4 Uncropped scans of blots and gels of western blot data.

Source Data Extended Data Fig. 4 Raw data for all graphs (separate sheet for each panel).

Source Data Extended Data Fig. 5 Uncropped scans of blots and gels of western blot data.

Source Data Extended Data Fig. 5 Raw data for all graphs (separate sheet for each panel).

Source Data Extended Data Fig. 6 Uncropped scans of blots and gels of western blot data.

Source Data Extended Data Fig. 6 Raw data for all graphs (separate sheet for each panel).

Source Data Extended Data Fig. 7 Uncropped scans of blots and gels of western blot data.

Source Data Extended Data Fig. 7 Raw data for all graphs (separate sheet for each panel).

Source Data Extended Data Fig. 8 Uncropped scans of blots and gels of western blot data.

Source Data Extended Data Fig. 8 Raw data for all graphs (separate sheet for each panel).

Source Data Extended Data Fig. 9 Raw data for all graphs (separate sheet for each panel).

Source Data Extended Data Fig. 10 Raw data for all graphs (separate sheet for each panel).

## Data Availability

Data supporting the findings of this study are available within the article, Extended Data figures, Source Data and Supplementary Tables. All data supporting the findings of this study are also available from the corresponding authors upon reasonable request. Materials generated in this study are available upon request. Zebrafish lines generated in this study have been registered on the ZFIN database and are available from ZFIN with a completed materials transfer agreement. Source data are provided with this paper.
