# Supplementary material for: Carbonic anhydrase inhibition ameliorates tau toxicity via enhanced tau secretion
Source: Nat Chem Biol. 2024 Oct 31;21(4):577–87. doi: 10.1038/s41589-024-01762-7 (PMC11949835; doi:10.1038/s41589-024-01762-7)
Supplement: Supplementary file 1 — Reporting Summary [file 41589_2024_1762_MOESM1_ESM.pdf]

nature portfolio

Corresponding author(s): David C. Rubinsztein and Angeleen Fleming

Last updated by author(s): Aug 15, 2024

Reporting Summary

Nature Portfolio wishes to improve the reproducibility of the work that we publish. This form provides structure for consistency and transparency in reporting. For further information on Nature Portfolio policies, see our Editorial Policies and the Editorial Policy Checklist.

Statistics

For all statistical analyses, confirm that the following items are present in the figure legend, table legend, main text, or Methods section.

n/a	Confirmed	
☐	☒	The exact sample size (*n*) for each experimental group/condition, given as a discrete number and unit of measurement
☐	☒	A statement on whether measurements were taken from distinct samples or whether the same sample was measured repeatedly
☐	☒	The statistical test(s) used AND whether they are one- or two-sided<br>*Only common tests should be described solely by name; describe more complex techniques in the Methods section.*
☒	☐	A description of all covariates tested
☒	☐	A description of any assumptions or corrections, such as tests of normality and adjustment for multiple comparisons
☐	☒	A full description of the statistical parameters including central tendency (e.g. means) or other basic estimates (e.g. regression coefficient) AND variation (e.g. standard deviation) or associated estimates of uncertainty (e.g. confidence intervals)
☐	☒	For null hypothesis testing, the test statistic (e.g. *F*, *t*, *r*) with confidence intervals, effect sizes, degrees of freedom and *P* value noted<br>*Give P values as exact values whenever suitable.*
☒	☐	For Bayesian analysis, information on the choice of priors and Markov chain Monte Carlo settings
☒	☐	For hierarchical and complex designs, identification of the appropriate level for tests and full reporting of outcomes
☒	☐	Estimates of effect sizes (e.g. Cohen's *d*, Pearson's *r*), indicating how they were calculated

Our web collection on statistics for biologists contains articles on many of the points above.

Software and code

Policy information about availability of computer code

Data collection

For zebrafish experiments:
- Leica Application Suite X (LAS-X) microscope imaging software (version 3.5.5.19976) was used during data acquisition on confocal for zebrafish.
- StepOne Plus Real Time PCR System and StepOneTM Software V.2.1 (Applied Biosystems, Life Technologies) was used to evaluate gene expression.
- Zeiss Axio Zoom.V16 microscope with a QImaging Retiga 2000 R digital camera and QCapture Pro 7.0 software were used for imaging of cryosections.
- LI-COR ODYSSEY Fc® and Image Studio software (version 5.2) was used to image sarkosyl soluble and insoluble tau fractions by western blot.
- Roche LightCycler® 480 machine using LightCycler® 480 Software (V1.5.1.62) for qPCR.
- FLUORstar Omega fluorometer (BMG Labtech) with Omega software (V6.20) for proteasome activity assay.

For cell experiments:
- SPARK multimode microplate reader with software SPARKCONTROL Method Editor version 3.0 for tau clearance assays, Lysosensor Yellow/Blue DND-160 detection also for HiBit/LDH/ELISA.
- LI-COR ODYSSEY CLX and Image Studio software (version 5.2) for western blot imaging.
- LSM880 Carl Zeiss confocal microscope, with ZEN Black software version 2.6 for imaging of lysosomal distribution.
- Nikon imaging analysis (Model: Nikon Ti2 ECLIPSE) with software NIS-Elements AR V. 5.41.02 for Tau-Venus aggregates.

For mice experiments:
- Q3 Analytical Ltd liquid chromatography–mass spectrometry (LC-MS) with Non Compartmental Pharmacokinetic Data Analysis software

(Windows 2.0.6 Excel 2002 Edition) for pharmacokinetic analyses by Q3 Analytical Ltd.
- Elx800 plate reader (Appleton Woods).
- Zeiss AxioImager Z2 microscope with Zen Blue version 3.3 Software for imaging immunostained brains.
- Behavioural video recording in mice was performed using a Logitech C310 HD Webcam camera connected with Panlab Smart Video Record it Software (Smart 3.0).

Data analysis

This study did not generate new softwares or code. Analytic softwares used as follows:
- Cell Profiler Analyst Data exploration software version 4.2.6 (Broad Institute) for LAMP1-positive puncta distribution.
- Fiji (Image J v. 1.54f) (National Institute of Health, USA) was used to analyse images from western blots, confocal images for clearance assays in fish, images for fish retinal degeneration.
- Western blots from cells and mice samples were analysed by IMAGE STUDIO Lite LI-COR version 5.2, Inc and Image J (National Institute of Health, USA).
- Genetic expression was analysed using LightCycler® 480 Software (V1.5.1.62) for SYBR Green experiments or StepOneTM Software V.2.1 (Applied Biosystems, Life Technologies) for Taqman assays.
- Non Compartmental Pharmacokinetic Data Analysis software (Windows 2.0.6 Excel 2002 Edition) for pharmacokinetic analyses by LC-MS Q3 Analytical Ltd.
- - Microscope imaging system (Olympus BX50 microscope) with software Stereo Investigator, version 11 (MicroBrightField (MBF) Bioscience) to quantify immunostained brains
- Videos from behavioral NORT were analysed manually using Behavioral Observation Research Interactive Software (BORIS version 8.25.4).
- Statistical analysis was performed using Prism v8-10 (Graphpad).
- Stereo Investigator software, version 11 (MicroBrightField (MBF) Bioscience), for the analyses of immunostained brains.
- Excel (Excel 2016 Microsoft Office)

For manuscripts utilizing custom algorithms or software that are central to the research but not yet described in published literature, software must be made available to editors and reviewers. We strongly encourage code deposition in a community repository (e.g. GitHub). See the Nature Portfolio guidelines for submitting code & software for further information.

Data

Policy information about availability of data

All manuscripts must include a data availability statement. This statement should provide the following information, where applicable:
- Accession codes, unique identifiers, or web links for publicly available datasets
- A description of any restrictions on data availability
- For clinical datasets or third party data, please ensure that the statement adheres to our policy

All data supporting the findings of this study are available from the corresponding author upon reasonable request. Source data are provided with this paper.

Human research participants

Policy information about studies involving human research participants and Sex and Gender in Research.

Reporting on sex and gender	This study did not include any human research participants
Population characteristics	N/A
Recruitment	N/A
Ethics oversight	N/A

Note that full information on the approval of the study protocol must also be provided in the manuscript.

Field-specific reporting

Please select the one below that is the best fit for your research. If you are not sure, read the appropriate sections before making your selection.

☒ Life sciences ☐ Behavioural & social sciences ☐ Ecological, evolutionary & environmental sciences

For a reference copy of the document with all sections, see nature.com/documents/nr-reporting-summary-flat.pdf

Life sciences study design

All studies must disclose on these points even when the disclosure is negative.

Sample size

No statistical methods were used to predetermine sample sizes but our sample sizes include a minimum of 3 samples per condition and N samples used in this study were similar to those reported in similar experiments from our previous publications. (e.g. Moreau K et al. Nature Communications 2014 ; Lopez A et al. Brain 2017 and Siddiqi FH Nature Communications 2019).

Data exclusions	No data was discarded from zebrafish/ cells or mice analyses.
Replication	Experiments with n = 3 were validated with a minimum of 3 replicates unless stated. Findings with high sample size were run in a single experiment (i.e. fish retinal degeneration of in vivo clearance kinetics). Multiple replicates are reported for each experiment when possible. Each replicate was performed at different times. Each replicate showed a similar effect/result.<br><br>For fish:<br>- One single replicate was used during the non-hypothesis-based primary screen (each sample containing 10 fish).<br>- To check levels of PHF1 phosphorylated tau, 7 independent clutches with 20 fish each were used.<br>- To measure Sarkosyl-soluble and insoluble tau, 3 clutches with 50 fish each were run together in same gel.<br>- To analyse the images of fish retinal degeneration a minimum of 32 eyes per group were quantified.<br>- To assess phenotypic abnormalities of fish, a minimum 6 clutches with 20 fish in each group was analysed.<br>- To measure in vivo tau clearance kinetics assays, a minimum of 49 neurons from the fish spinal cord per group.<br><br>For cell experiments:<br>- To measure tau secretion all experiments had n=3 in triplicates.<br>- A minimum of 40 cells/condition were used to analyse lysosomal distribution in cell culture.<br>For mice experiments:<br>- 5 mice were used for the analysis of methazolamide effects on Tg4510 mice.<br>- 19 mice were used for the analysis soluble/insoluble tau after drug treatment in PS19 mice.<br>- 15 mice were used for the behavioral assay in PS19.<br>- 5 mice were used for the immuno-histochemistry analysis in PS19.
Randomization	Zebrafish experiments were performed by dividing the clutches into the different experimental groups to have equal number of siblings per condition. Fish were used at a stage where fish cannot be differentiated by sex so a mix population of larvae was used.<br><br>Mice were assigned treatment group in a factorial randomised design, for instance mice receiving the methazolamide treatment were in the same cages with mice receiving the vehicle treatment and WT littermates. Both genders were included for both mouse models, i.e., rTg4510 and PS19.<br><br>For Cell lines: Most experiments were done in single cell lines. Samples were allocated randomly into experimental groups.
Blinding	Blinding was only relevant for biased experiments for which analyses depended on the investigator's observations.<br>Blinding was relevant for the analysis of morphological defects in fish expressing Dendra-tau in which the investigator evaluated the severity of the defects based on visual observation. The investigator was blind to group allocation by another member of the lab, and treatments were not revealed until the experiment was finished.<br>The experimenter was also blind for the treatment groups of mice for the behavioral testing and analysis of PS19 mice.<br>Investigators were not blinded during the other experiments.

Reporting for specific materials, systems and methods

We require information from authors about some types of materials, experimental systems and methods used in many studies. Here, indicate whether each material, system or method listed is relevant to your study. If you are not sure if a list item applies to your research, read the appropriate section before selecting a response.

Materials & experimental systems

n/a	Involved in the study	
☐	☒	Antibodies
☐	☒	Eukaryotic cell lines
☒	☐	Palaeontology and archaeology
☐	☒	Animals and other organisms
☒	☐	Clinical data
☒	☐	Dual use research of concern

Methods

n/a	Involved in the study	
☒	☐	ChIP-seq
☒	☐	Flow cytometry
☒	☐	MRI-based neuroimaging

Antibodies

Antibodies used	anti- CA IV (G-11) (Santa Cruz; sc-74527, 1:500)<br>anti-ARL8B (Abcam;ab207697, 1:1000)<br>anti-arrestin-3 (ZIRC Zpr-1, 1:500)<br>anti-CA IX (H-11) (Santa Cruz sc-365900, 1:500)<br>anti-GAPDH (Novus Biologicals NB100-56875, 1:1000)<br>anti-GFP (Abcam ab6556, 1:1000)<br>anti-LAMP1 (Cell Signaling Technology #9091, 1:500)

anti-LC3 (Novus Biologicals NB100-2220, 1:1000)
anti-PHF1 (gift from Dr. Peter Davies, 1:100)
anti-phospho tau AT8 (Pierce, Thermo Scientific #MN1020, 1:50)
anti-NeuN (Novus, #NBP1-92693APC, 1:5000)
anti-rhodopsin (ZIRC Zpr-3, 1:250)
anti-rabbit HRP-linked antibody (Cell Signaling Technology # 7074, 1:5000)
anti-Tau5 (Abcam ab80579, 1:1000)
anti-Tubulin (Sigma-Aldrich #T6199, 1:5000)
anti-VAMP7 (Sigma-Aldrich SAB2105695, 1:1000)
anti-β-actin (Sigma-Aldrich A2066, 1:1000)
anti-β-actin (Sigma-Aldrich A5316, 1:1000)
Goat anti-mouse HRP ()Agilent P044701-2, 1:5000)
Goat anti-rabbit HRP (Agilent P044801-2, 1:5000)
Goat-anti-rabbit Alexa Fluor 594 (ThermoFisher Scientific A11012, 1:500)
Goat-anti-mouse Alexa Fluor 568 (ThermoFisher Scientific A11019, 1:1000)

Validation

All antibodies used in this study were purchased from commercial vendors who had validated specificity in human cells/ mouse and zebrafish tissues for the specific assays (Western blot, immunoprecipitation and/or immunofluorescence). It is described on data sheets and online.

Eukaryotic cell lines

Policy information about cell lines and Sex and Gender in Research

Cell line source(s)

SH-SY5Y (ECACC # 94030304)
Tetracycline-Inducible SH-TauP301L from Yung-Feng Liao's group in Taiwan
HEK293 (parental cells, ECACC; #85120602)and HEK293 cells expressing P301S tau-venus cells from McEwan Lab in Cambridge.

Authentication

SH-SY5Y authenticated by ATCC (DNA barcoding method; FTA Barcode: STRA1440)
HEK293 authenticated by LGC (STR profiling, FTA Barcode: STRA1472)
Tetracycline-Inducible SH-TauP301L and HEK293 cells expressing P301S tau-venus cells are stable cell lines that are routinely validated in experiments where we confirm expression of the relevant transgenic protein products.

Mycoplasma contamination

The cells were regularly tested using EZ-PCR Mycoplasma Test Kit (Biological Industries; cat#20-700-20) and Mycrostrip100 (InvivoGen- rep-mys-100). Cells used in this study were mycoplasma negative.

Commonly misidentified lines
(See ICLAC register)

None

Animals and other research organisms

Policy information about studies involving animals; ARRIVE guidelines recommended for reporting animal research, and Sex and Gender in Research

Laboratory animals

Zebrafish lines (all from Zebrafish Information Network (ZFIN)):
atg7sa14768 , RRID: ZFIN_ZDB-ALT-130411-3442), adults 6-18 month-old used for outcross
PanN:Gal4 (s1101tEt), RRID: ZFIN_ZDB-ALT-070716-2, adults 6-18 month-old used for outcross
Tg(rho:EGFP-Hsa.MAPT)cu7, RRID: ZFIN_ZDB-ALT-121206-4, adults 6-18 month-old used for crosses and zebrafish larvae from 0 - 10 days post-fertilisation (10 d.p.f.) for experiments.
Tg(rho:EGFP-Hsa.MAPT_P301L)cu12, RRID: ZFIN_ZDB-ALT-190304-2, adults 6-18 month-old used for crosses and zebrafish larvae from 0 - 10 days post-fertilisation (10 d.p.f.) for experiments
Tg(UAS:Dendra2-Hsa.MAPT,myl7:EGFP)cu9, RRID: ZFIN_ZDB-ALT-170616-3, adults 6-18 month-old used for outcross
Tg(UAS:Dendra2-Hsa.MAPT_A152T,myl7:EGFP)cu10, RRID: ZFIN_ZDB-ALT-170616-4, adults 6-18 month-old used for outcross
Tg(UAS:Dendra2-Hsa.MAPT_P301L,myl7:EGFP)cu61, RRID: ZFIN_ZDB-ALT-220906-6, adults 6-18 month-old used for outcross
Tg2(rho:EGFP)cu3, RRID: ZFIN_ZDB-ALT-101103-1, adults 6-18 month-old used for crosses and zebrafish larvae from 0 - 10 days post-fertilisation (10 d.p.f.)
Tg3(Xla.Eef1a1:GAL4-VP16)cu11, RRID: ZFIN_ZDB-ALT-170616-5 adults 6-18 month-old used for outcross

Adult fish between 6 months and 18 months old were bred to generate embryos and larvae for the experiments described below:
Zebrafish larvae from 0 - 10 days post-fertilisation (10 d.p.f.) were used for Fig 1 a&b, Fig 2b-e, Fig 3e, Ext Fig 2, Ext Fig 3e.
Zebrafish larvae from 0 -3 d.p.f. were used for Fig 1c&e, Fig 2a&f, Fig 3a-d, Ext Fig 3a,b&d,Ext Fig 4b, Ext Fig 5d&e
Zebrafish larvae from 0 - 6 d.p.f. were used for Fig 1f&g, Ext Fig 3c.
Zebrafish larvae at 0 - 9 d.p.f. were used for Ext Fig 4a.
Zebrafish larvae at 0 - 4 d.p.f. were used for Ext Fig 4c.
Zebrafish larvae at 0 - 2 d.p.f. were used for Ext Fig 5a-c

Mouse lines:
B6N.Cg-Tg(Prnp-MAPT*P301S)PS19Vle/J (PS19) (The Jackson Laboratory, RRID:IMSR_JAX:024841): 6 weeks old mice were crossed with 6 weeks old wild-type C57 BL/6J mice (The Jackson Laboratory, RRID:IMSR_JAX:000664), to maintain the line. Heterozygous

PS19 mice (both gender) were used in experiments at 34-38 week of age (Fig 6b-d, Ext Fig 9a) and 38-42 weeks old (Fig 6e-h, Ext Fig 9b-c).

C57/Bl6J (The Jackson Laboratory, RRID:IMSR_JAX:000664): adult males 3-4 months old Ext Fig 8a-b.

FVB-Tg(tetO-MAPT*P301L)#Kha/JlwsJ (The Jackson Laboratory, RRID:IMSR_JAX:015815): adults 6-7 weeks old crossed with 6-7 weeks old Tg(Camk2a-tTA)1Mmay mice (The Jackson Laboratory, RRID:IMSR_JAX:007004) to generate rTg4510 line. Both genders sTg4510 mice were used 3.5 to 4 months old are used in Fig 5a-e.

Wild animals	This study did not involved the use of wild animals
Reporting on sex	For zebrafish sex cannot be differented at the stages experiments were done. No discrimination of sex was used for mice studies.
Field-collected samples	No field-collected samples were used in the study.
Ethics oversight	All zebrafish experiments were performed in accordance with the UK Animals (Scientific Procedures) Act with appropriate Home Office Project and Personal animal licenses and with University of Cambridge Animal Welfare and Ethical Review Body (AWERB) approval. Studies were performed in accordance with PREPARE and ARRIVE guidelines.<br>Mouse studies were performed in accordance with the UK Animals (Scientific Procedures) Act with appropriate Home Office Project and Personal animal licenses and with University of Cambridge Animal Welfare and Ethical Review Body (AWERB) approval. Mice were maintained and used in experiments following PREPARE and ARRIVE guidelines

Note that full information on the approval of the study protocol must also be provided in the manuscript.
