# Supplementary figures and images for: Carbonic anhydrase inhibition ameliorates tau toxicity via enhanced tau secretion
Source: Nat Chem Biol. 2024 Oct 31;21(4):577–87. doi: 10.1038/s41589-024-01762-7 (PMC11949835; doi:10.1038/s41589-024-01762-7)

Fig.1d

d

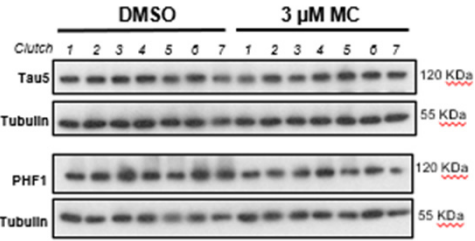

Tau5  
Tubulin

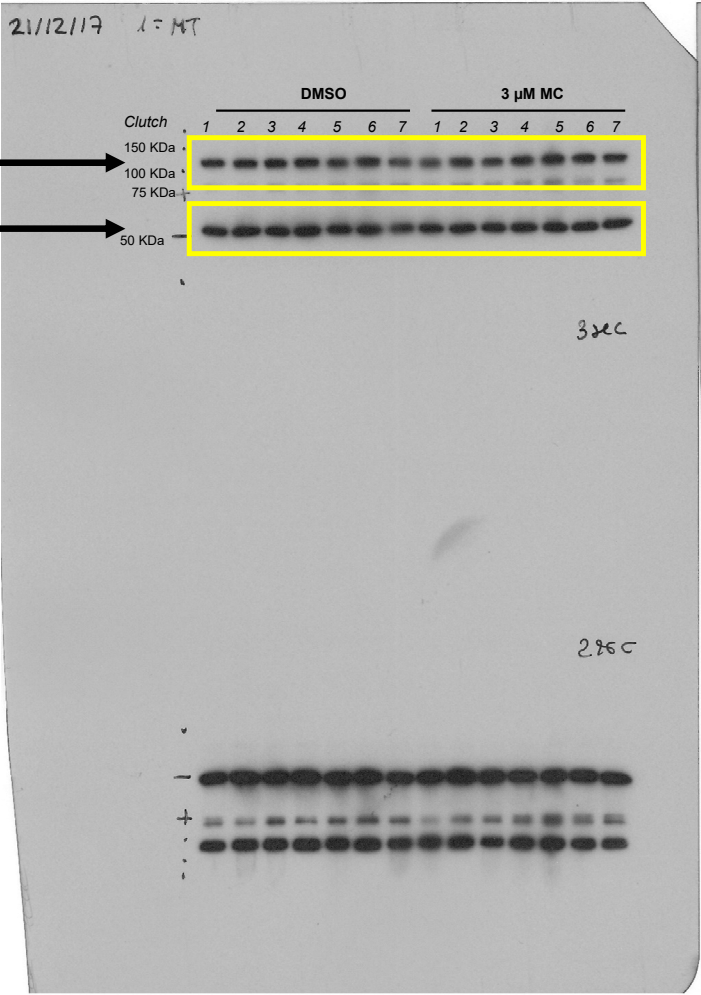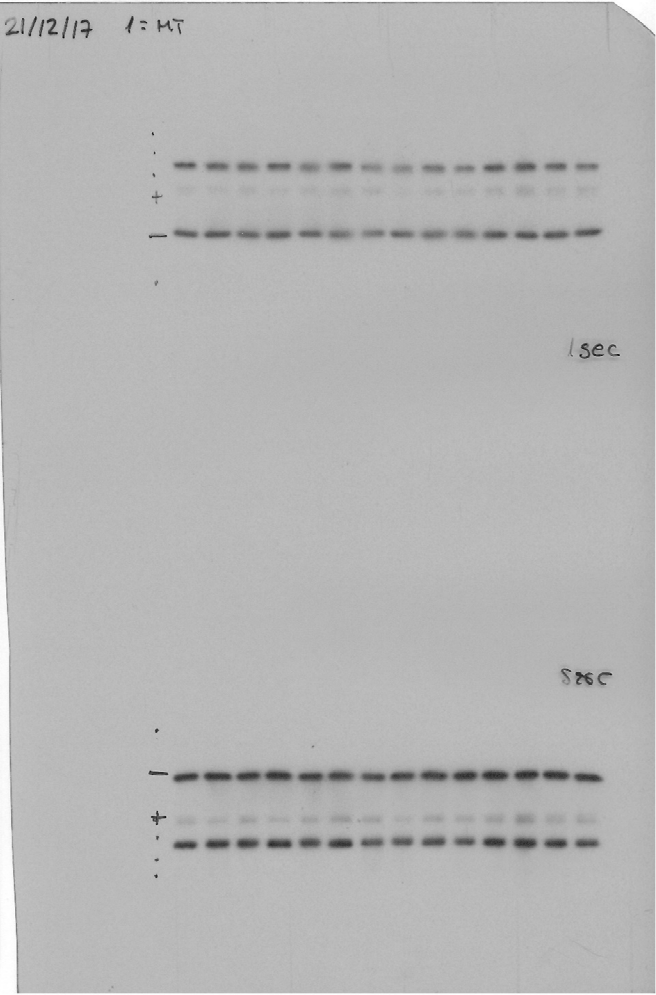

Fig.1d

d

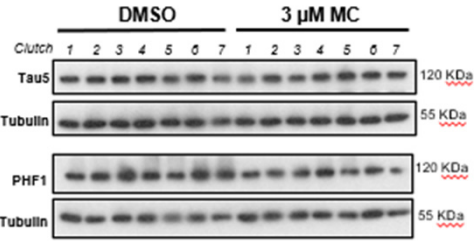

PHF1

Tubulin

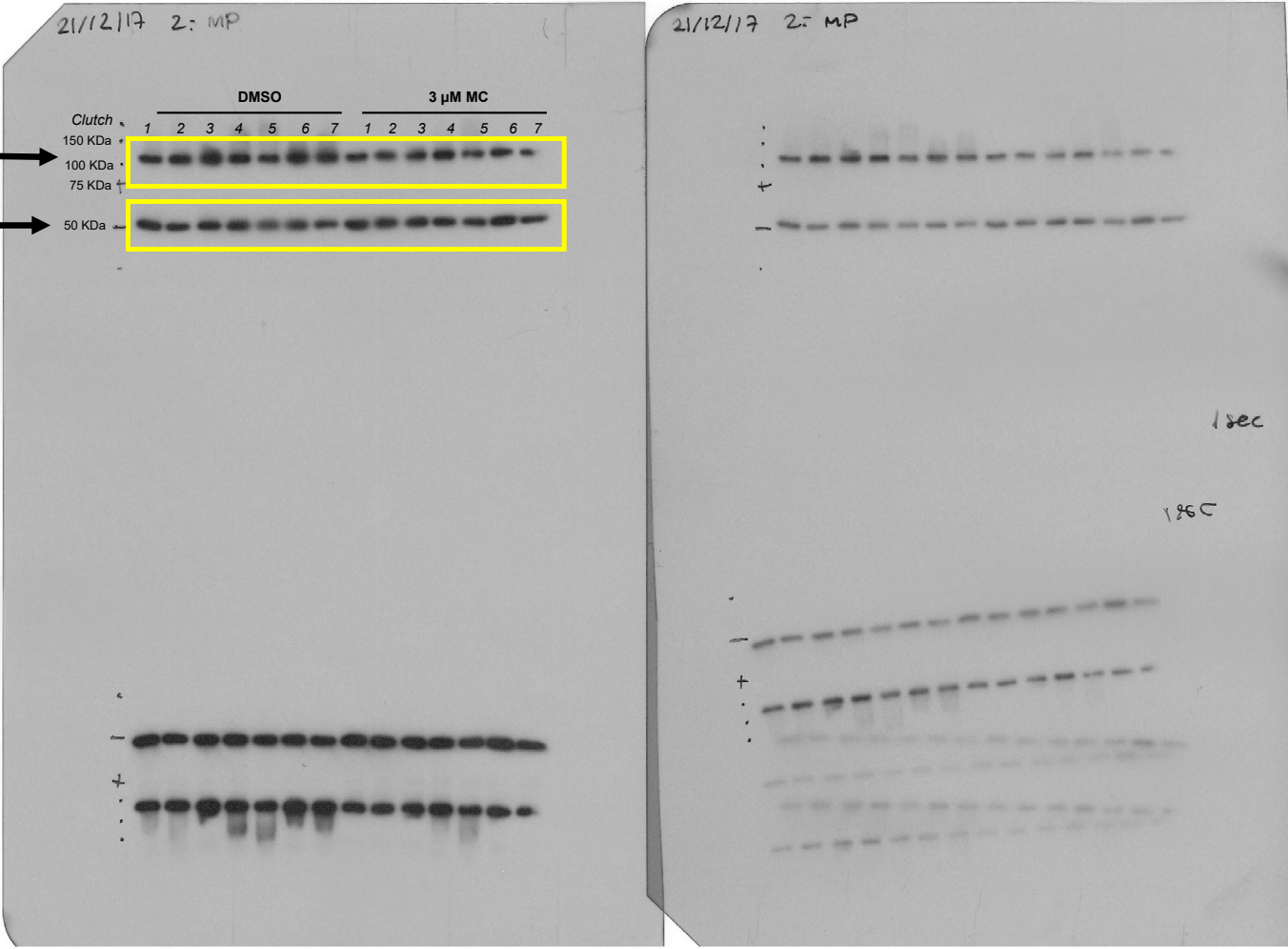

Fig.1f

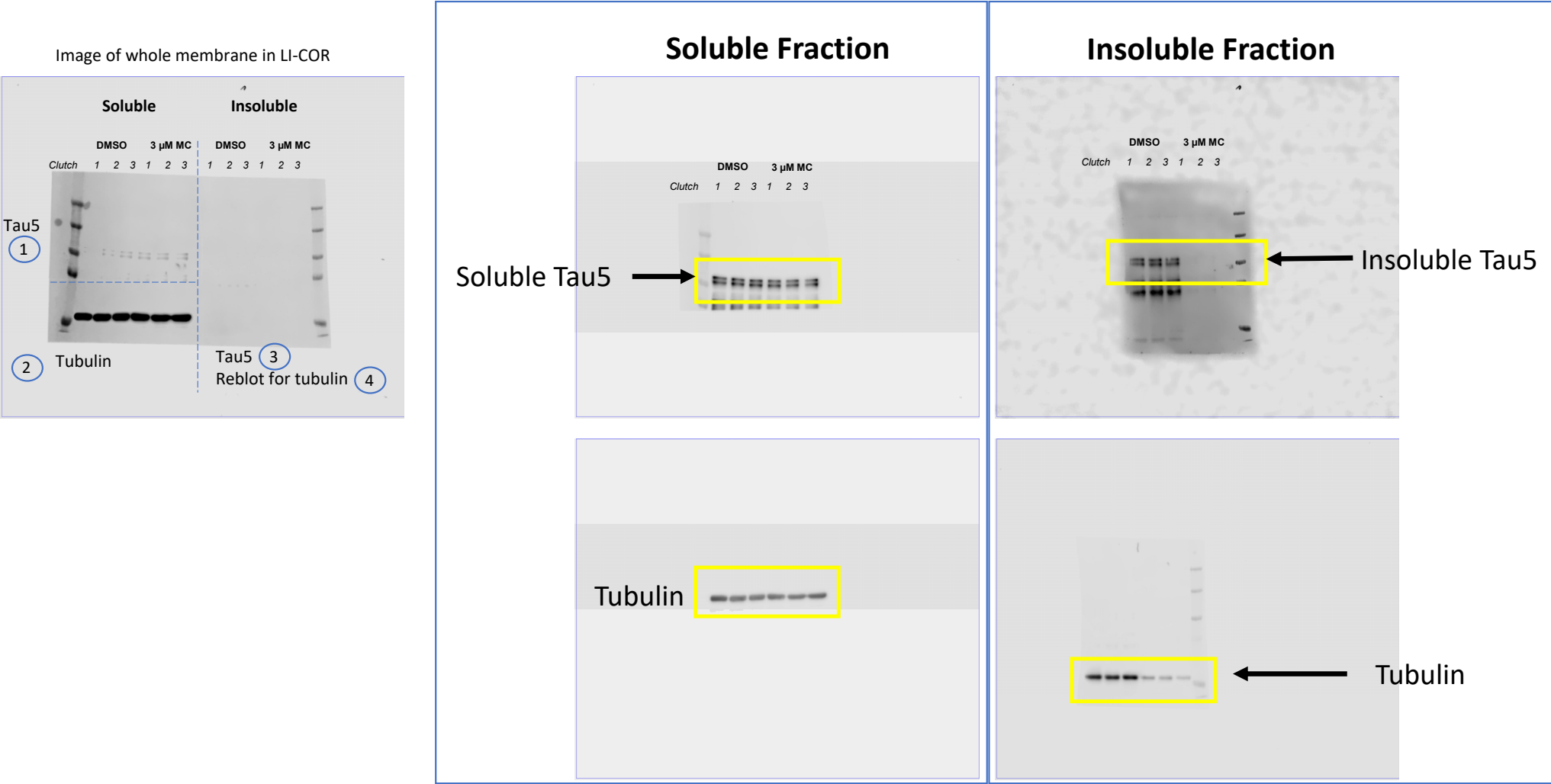

Supplement: Supplementary file 3 — Uncropped scans of blots and gels of western blot data. [file 41589_2024_1762_MOESM3_ESM.pdf]

Fig 4a.i.

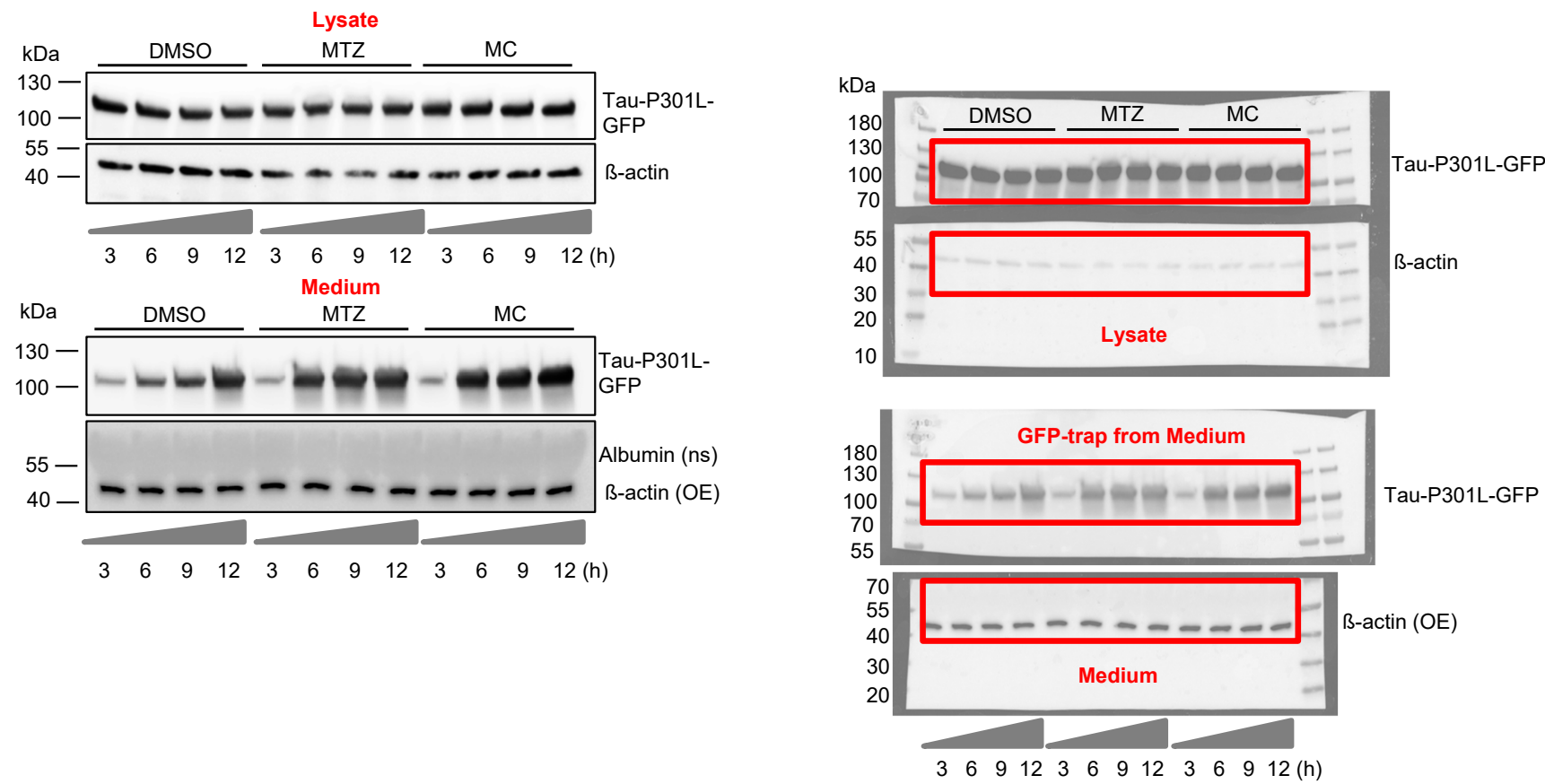

Supplement: Supplementary file 7 — Uncropped scans of blots and gels of western blot data. [file 41589_2024_1762_MOESM7_ESM.pdf]

Fig6b

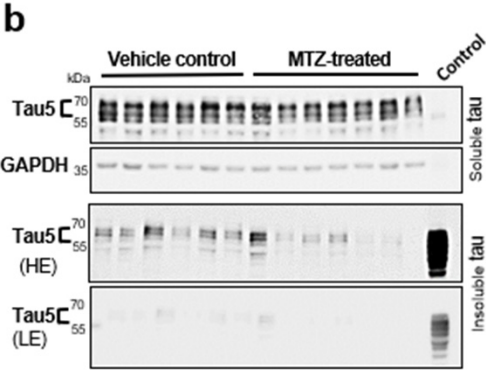

Tau 5 soluble Fraction

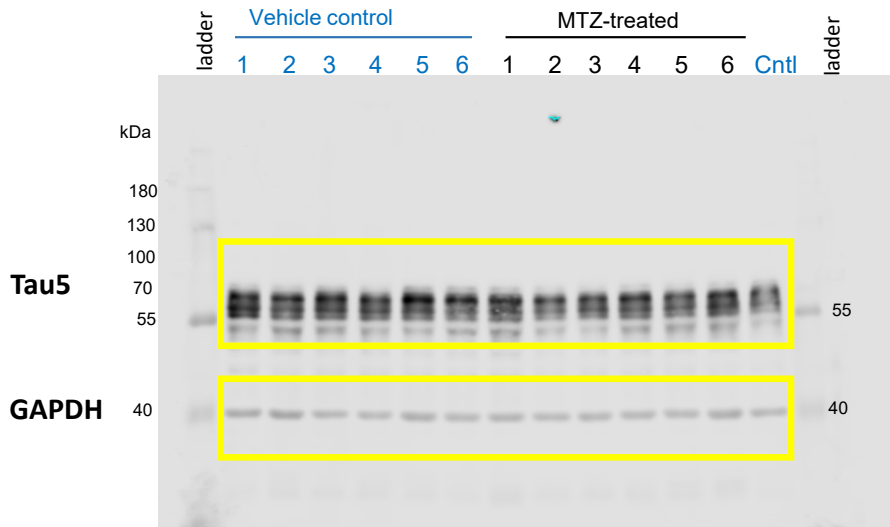

Tau 5 insoluble Fraction

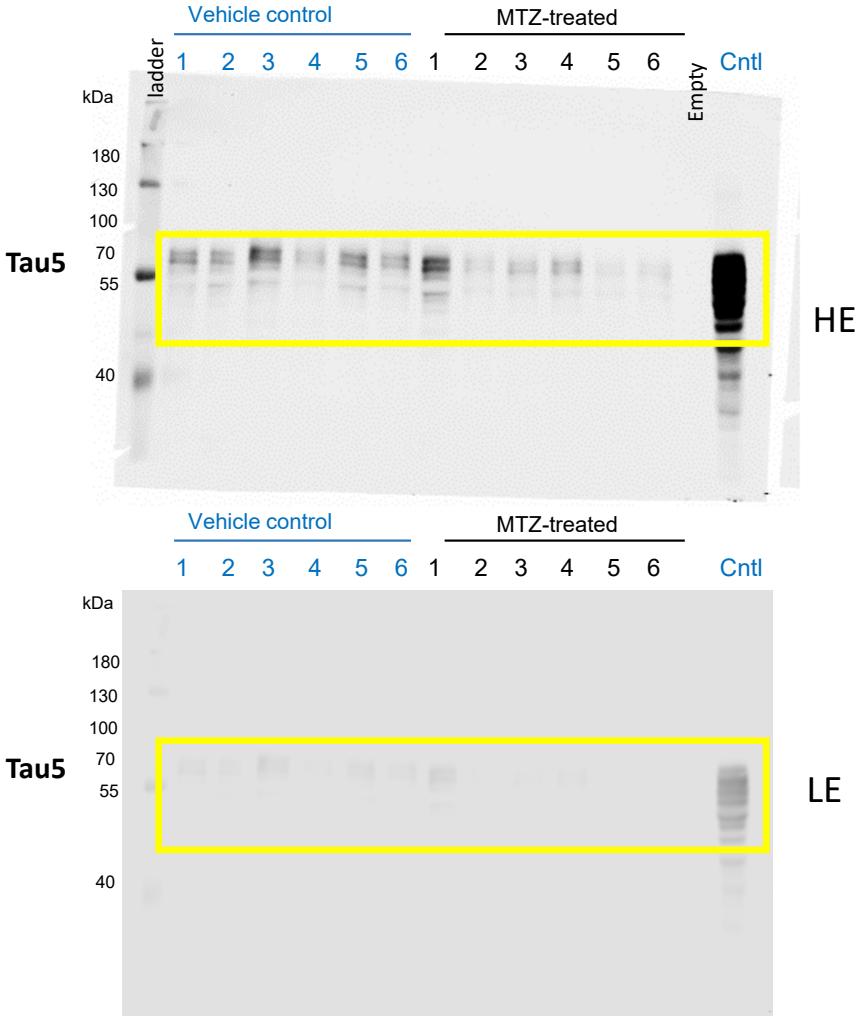

Supplement: Supplementary file 11 — Uncropped scans of blots and gels of western blot data. [file 41589_2024_1762_MOESM11_ESM.pdf]

Ext. Data Fig 2a

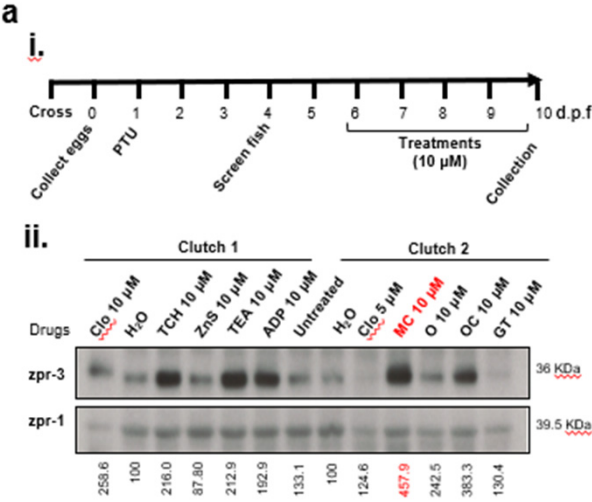

Zpr1 (top)

Zpr3 (bottom)

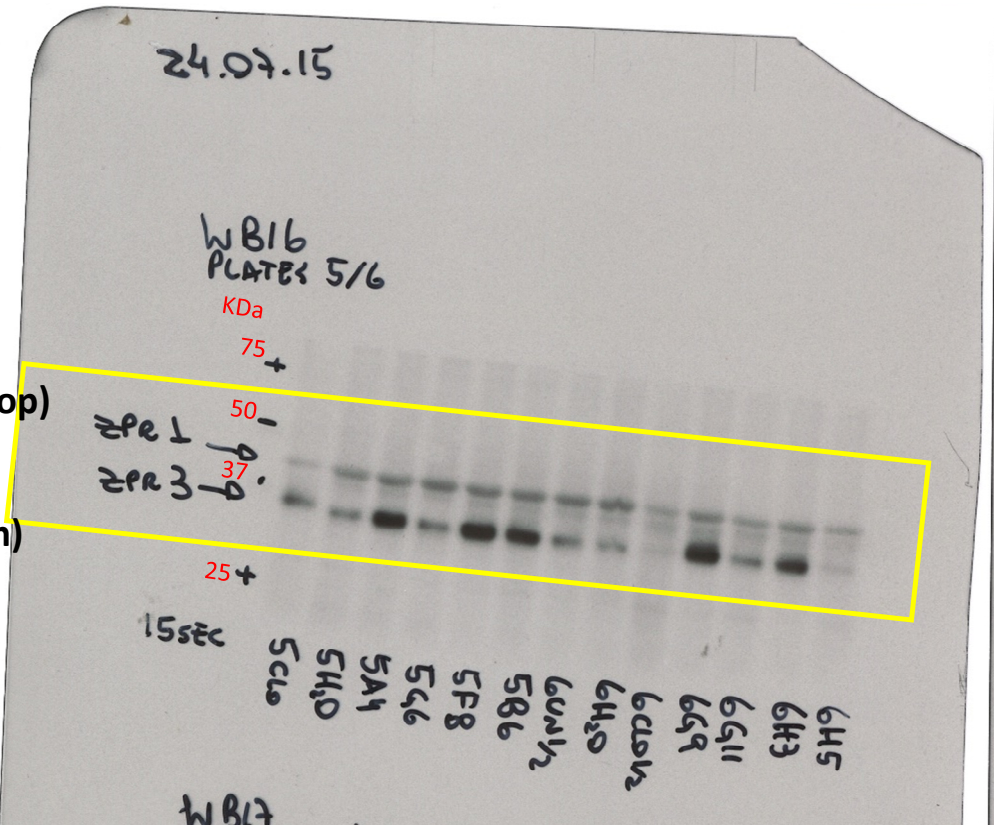

Ext. Fig 2b

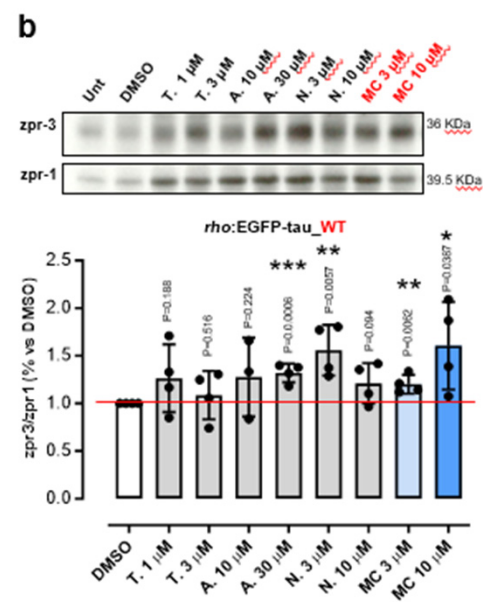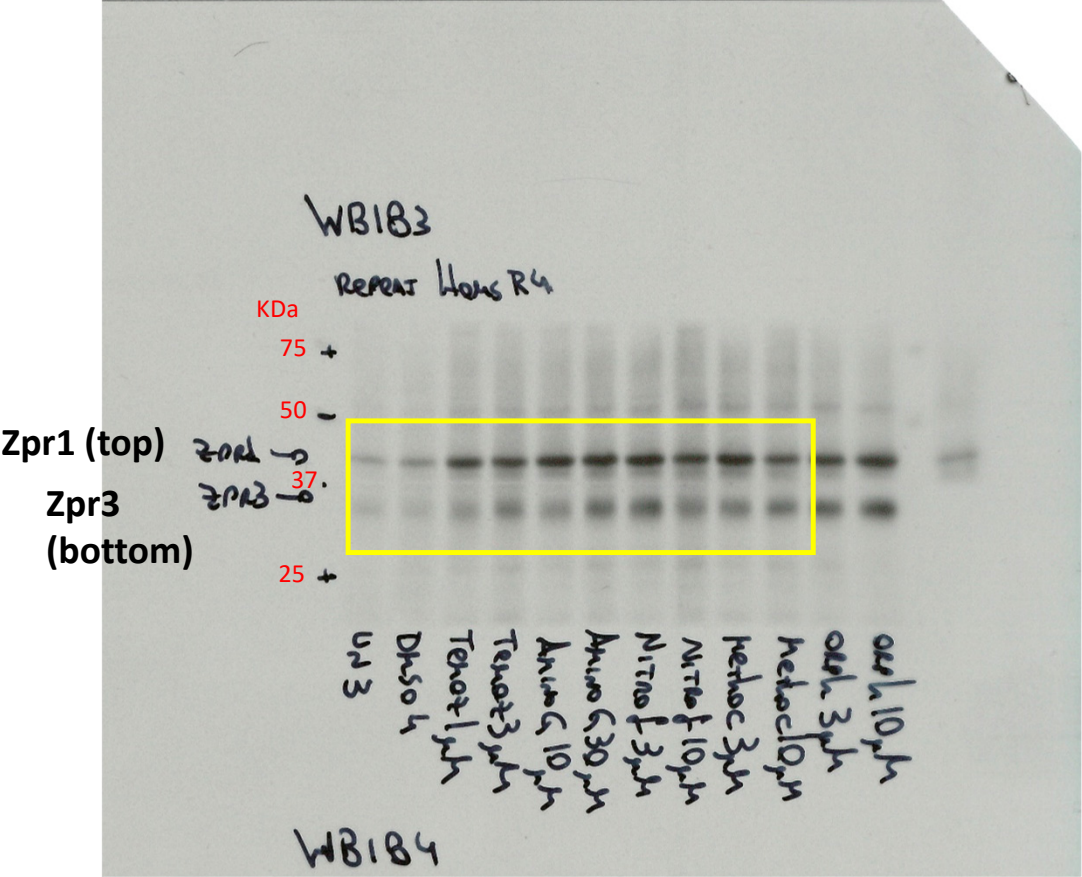

Ext. Fig 2c

c

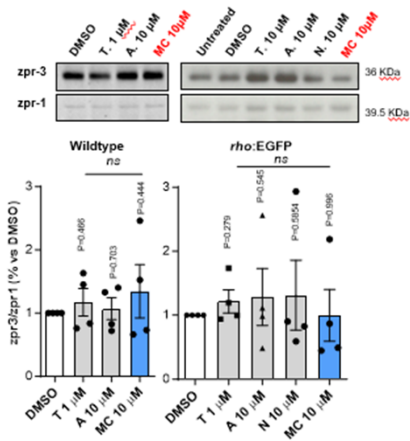

Wildtype

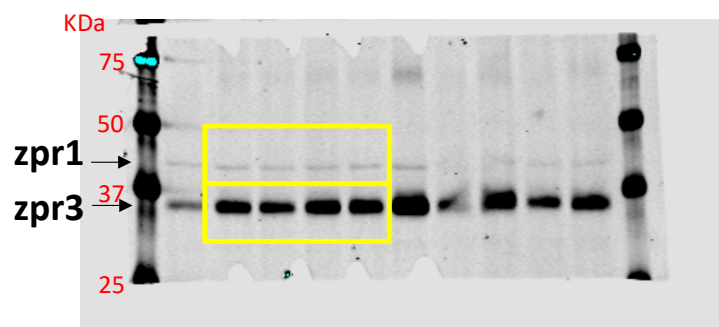

Rho:EGFP

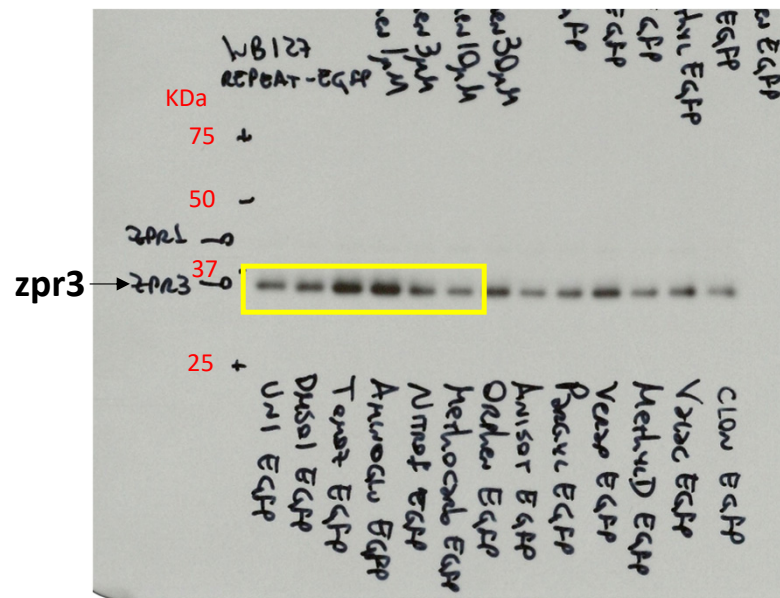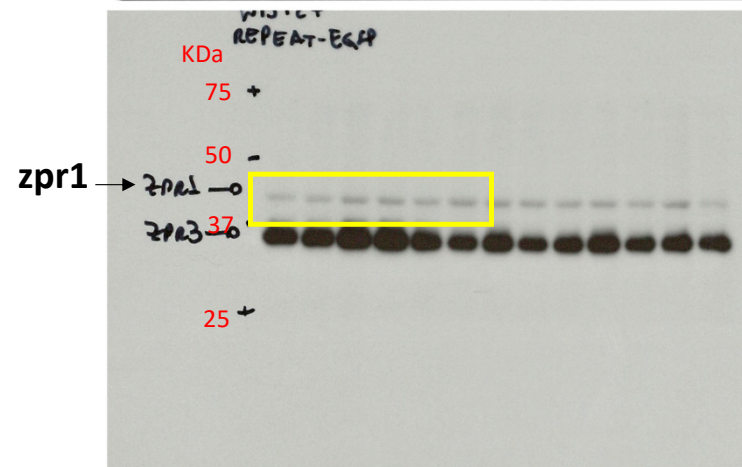

Supplement: Supplementary file 14 — Uncropped scans of blots and gels of western blot data. [file 41589_2024_1762_MOESM14_ESM.pdf]

Ext. Data Fig 6Ci.

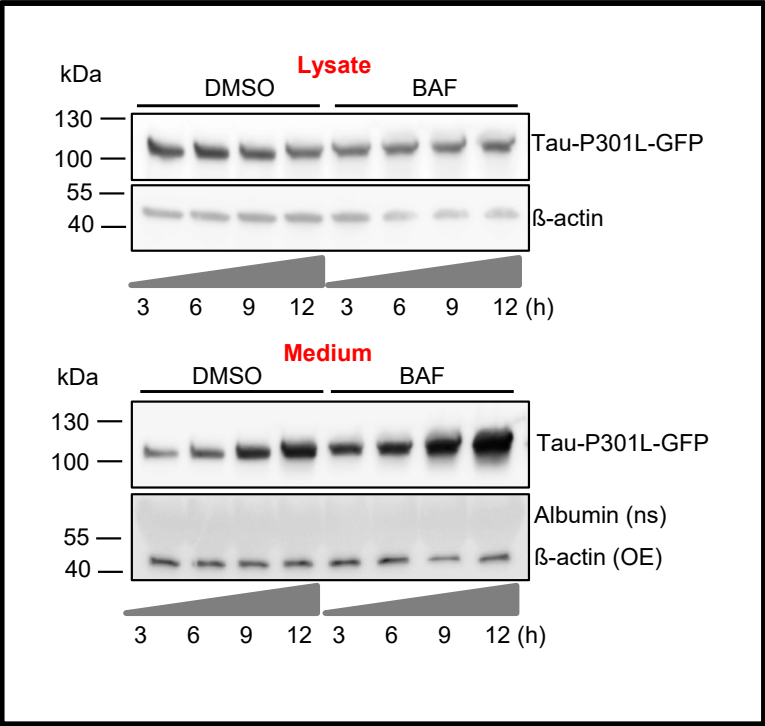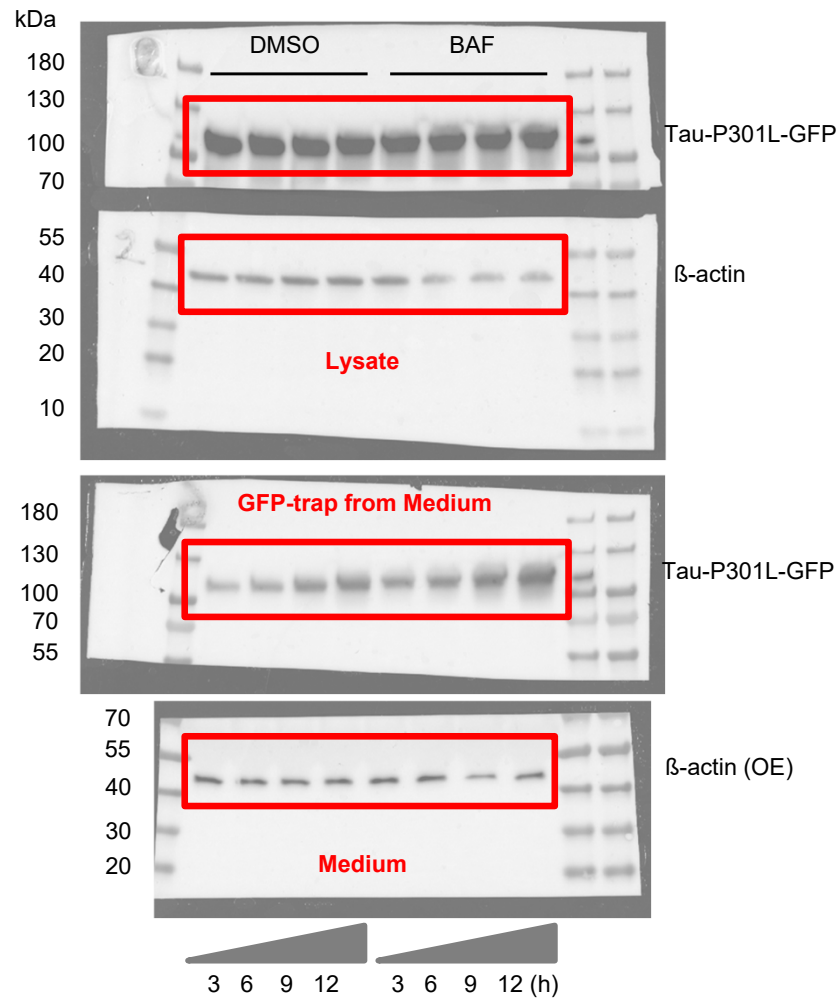

Supplement: Supplementary file 22 — Uncropped scans of blots and gels of western blot data. [file 41589_2024_1762_MOESM22_ESM.pdf]

Ext. Data Fig 7bi.

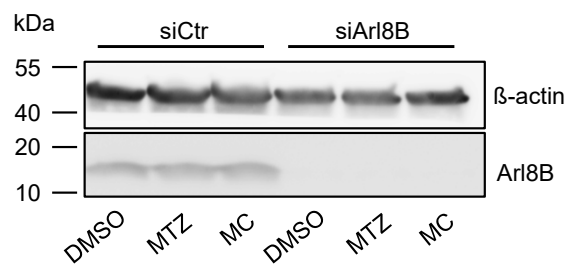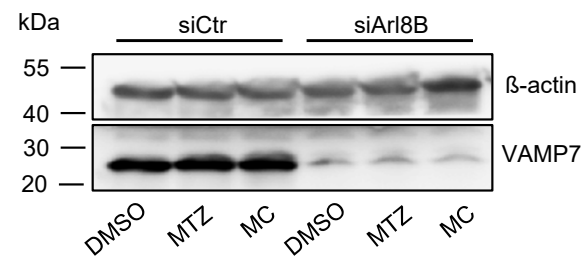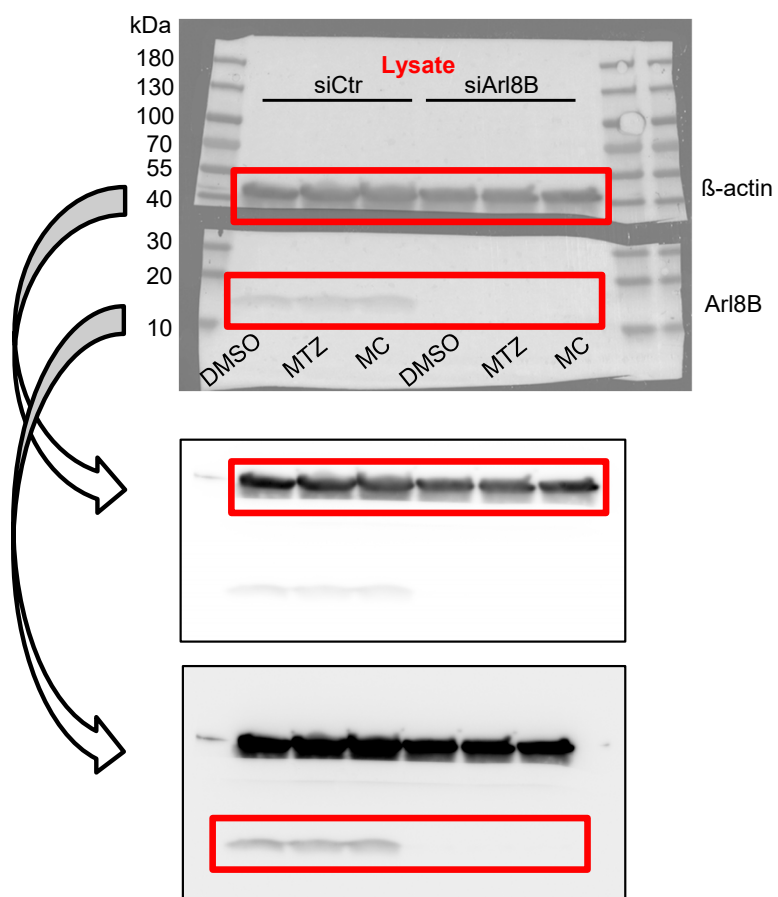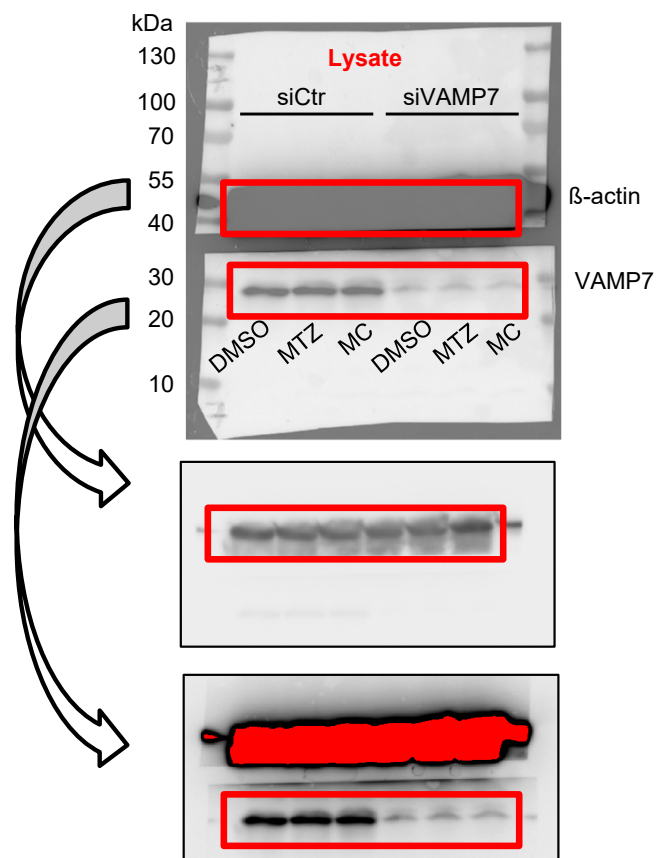

Supplement: Supplementary file 24 — Uncropped scans of blots and gels of western blot data. [file 41589_2024_1762_MOESM24_ESM.pdf]
