# Supplementary material for: Carbonic anhydrase inhibition ameliorates tau toxicity via enhanced tau secretion
Source: Nat Chem Biol. 2024 Oct 31;21(4):577–87. doi: 10.1038/s41589-024-01762-7 (PMC11949835; doi:10.1038/s41589-024-01762-7)
Supplement: Supplementary file 9 — Uncropped scans of blots and gels of western blot data. [file 41589_2024_1762_MOESM9_ESM.pdf]

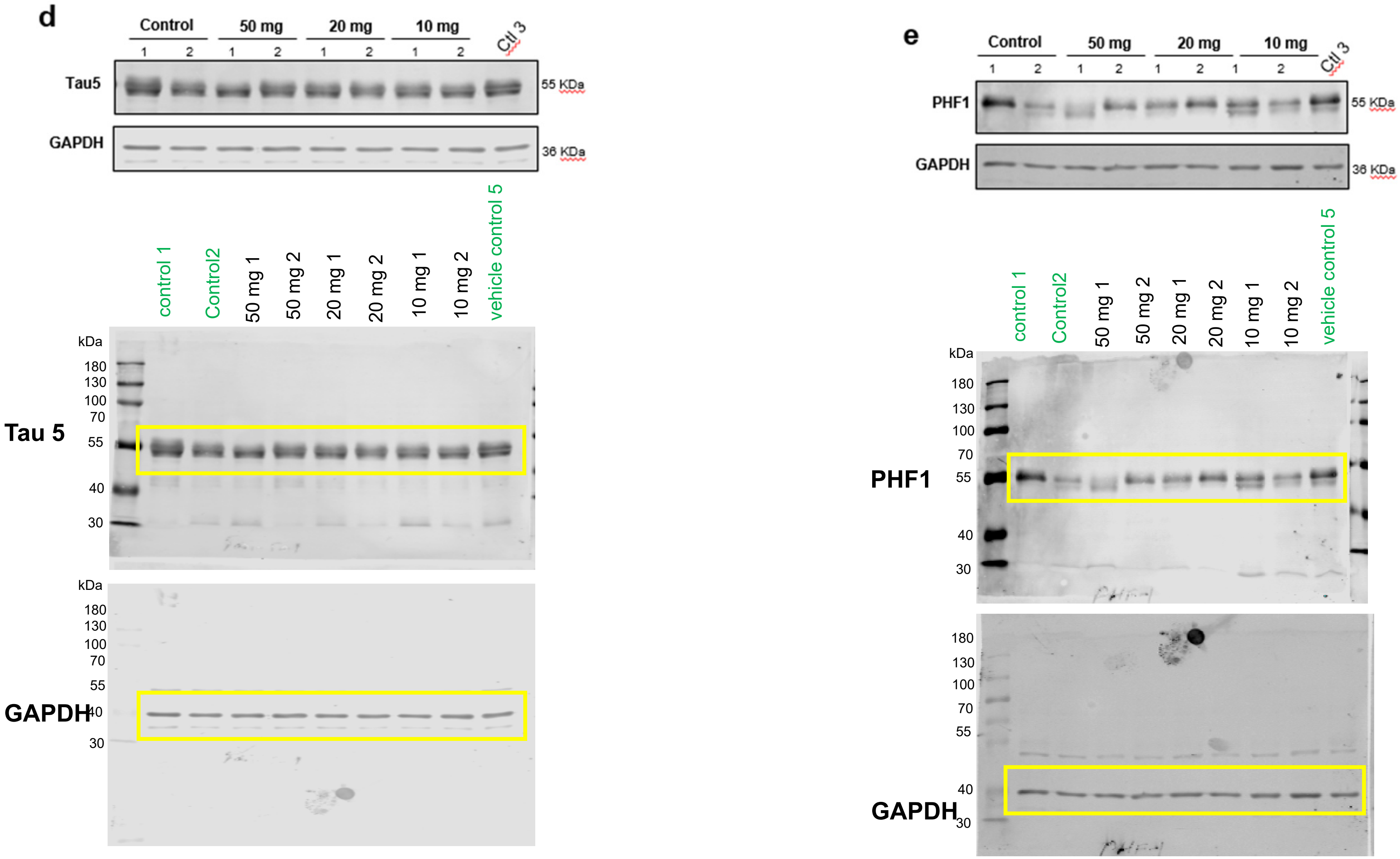
d
Control
50 mg
20 mg
10 mg
1 2 1 2 1 2 1 2
Ctl 3
Tau5
55 KDa
GAPDH
36 KDa
control 1
Control2
50 mg 1
50 mg 2
20 mg 1
20 mg 2
10 mg 1
10 mg 2
vehicle control 5
kDa
180
130
100
70
55
40
30
Tau 5
GAPDH
e
PHF1
55 KDa
36 KDa
