# Supplementary material for: Carbonic anhydrase inhibition ameliorates tau toxicity via enhanced tau secretion
Source: Nat Chem Biol. 2024 Oct 31;21(4):577–87. doi: 10.1038/s41589-024-01762-7 (PMC11949835; doi:10.1038/s41589-024-01762-7)
Supplement: Supplementary file 16 — Uncropped scans of blots and gels of western blot data. [file 41589_2024_1762_MOESM16_ESM.pdf]

Ext Data Fig 3c

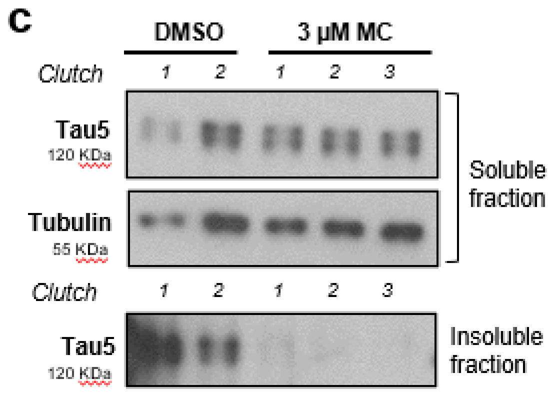

Image used for Soluble fractions (Low exposure)

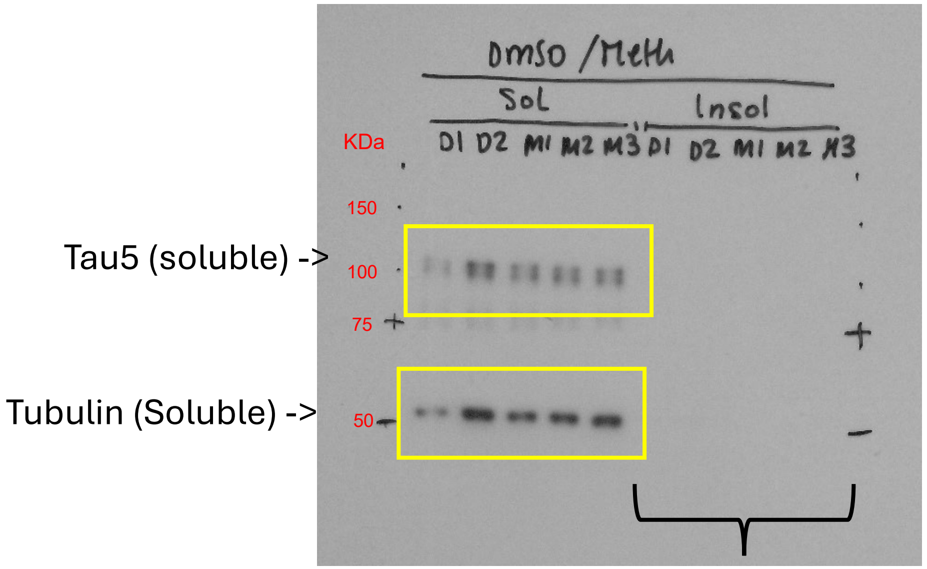

Insoluble fraction not visible with short exposures

Image used for Insoluble fractions (High exposure)

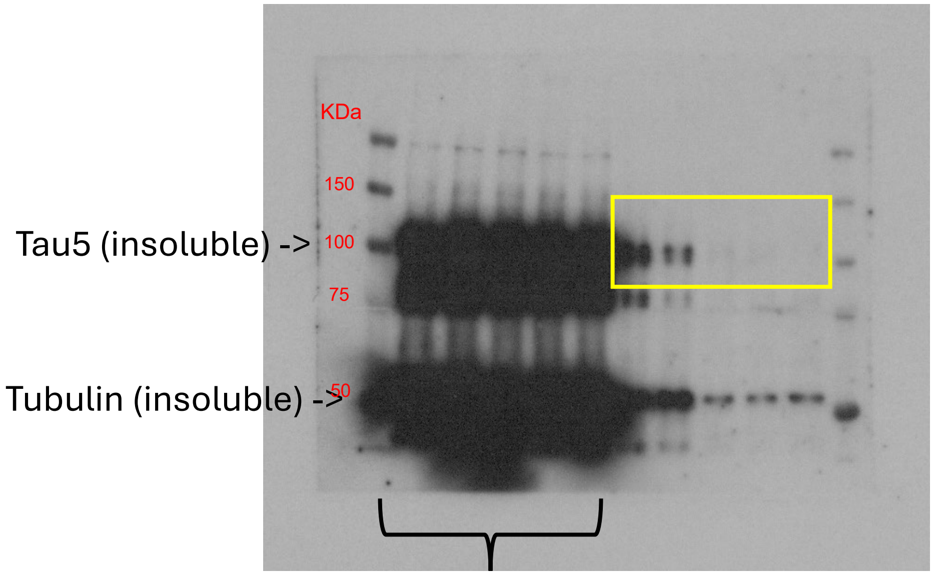

Soluble fraction over-exposed
