# Supplementary material for: Carbonic anhydrase inhibition ameliorates tau toxicity via enhanced tau secretion
Source: Nat Chem Biol. 2024 Oct 31;21(4):577–87. doi: 10.1038/s41589-024-01762-7 (PMC11949835; doi:10.1038/s41589-024-01762-7)

Extended Data Fig 4a. Carbonic anhydrases expressed in the eye/body at 9dpf

ca2, cahz, ca4a, ca5, ca9 and ca14

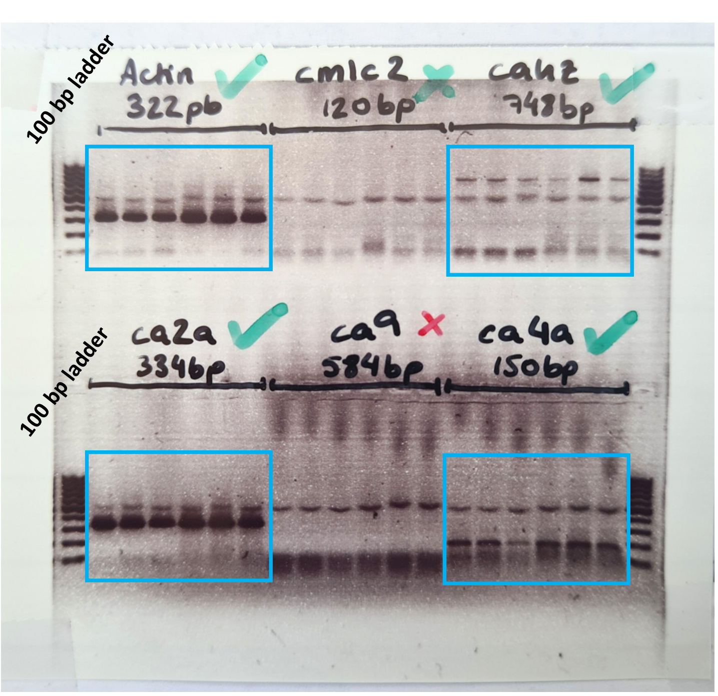

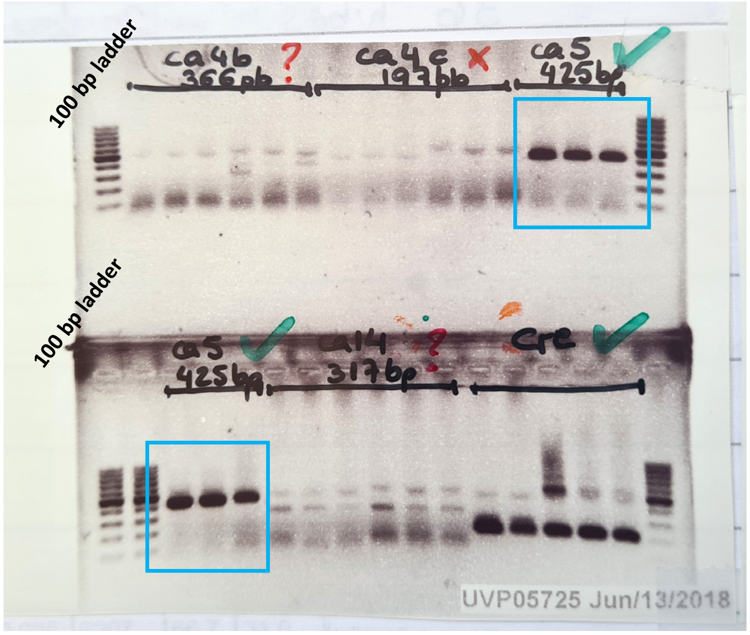

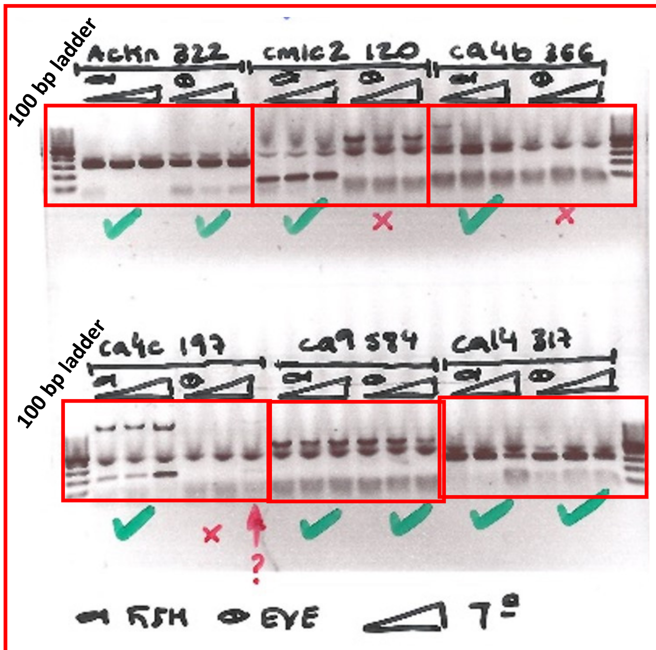

Images of whole agarose gels represented in the second and third rows of the figure panel (cropped images seen in figure are highlighted by red rectangles)

Images of whole agarose gels represented in the top row of the figure panel (cropped images seen in figure are highlighted by blue rectangles)

Extended Data Fig 4b. Carbonic anhydrases expressed in FAC-sorted neurons

ca2, cahz, ca4a, ca5, ca9 and ca14

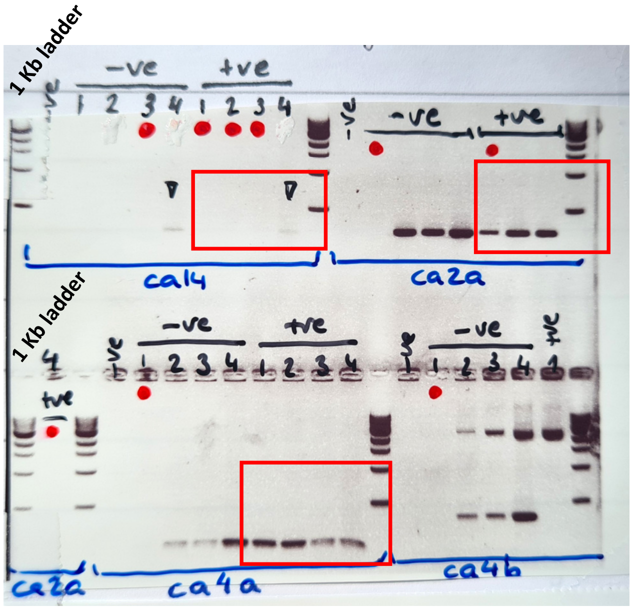

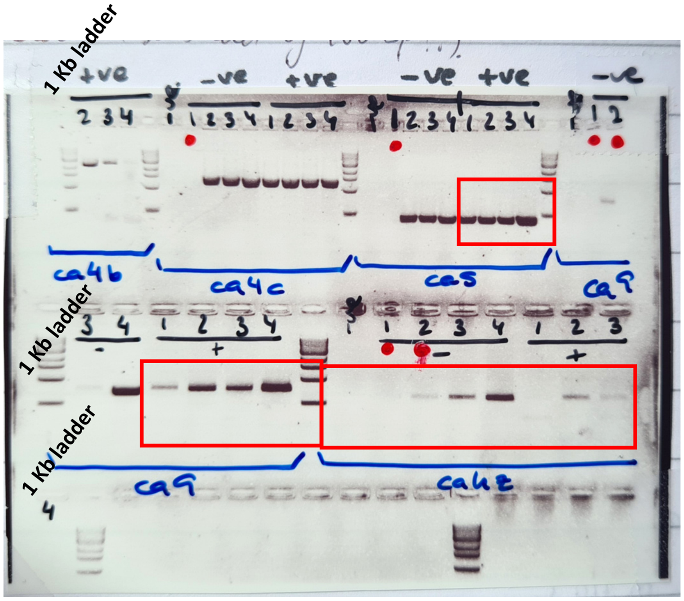

Images of whole agarose gels represented the figure panel (cropped images seen in figure are highlighted by red rectangles)

Extended Data Fig. 4D. Carbonic anhydrases protein levels in zebrafish

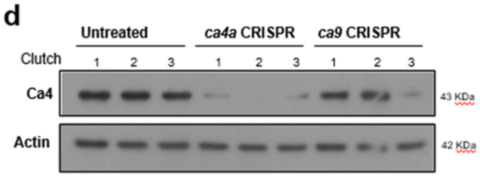

Rabbit anti- CA IV (G-11) for carbonic anhydrase 4 (1:500, #sc-74527 Santa Cruz).

ca4

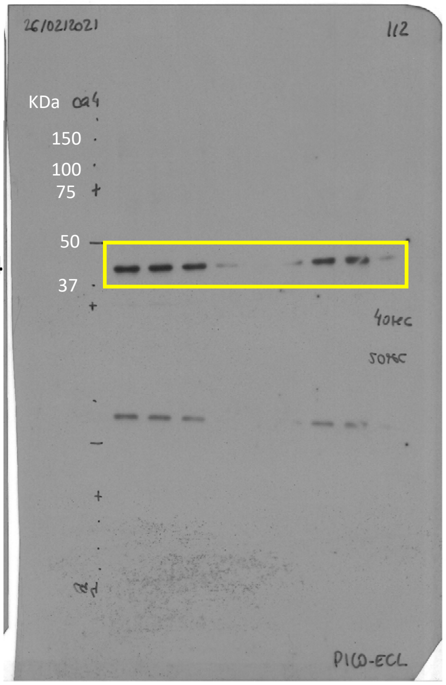

Mouse anti-β-actin (1:1000; #A5316 Sigma-Aldrich).

Actin

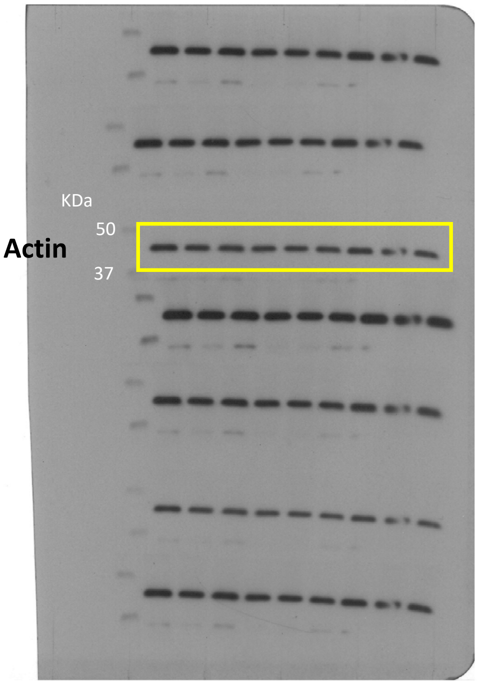

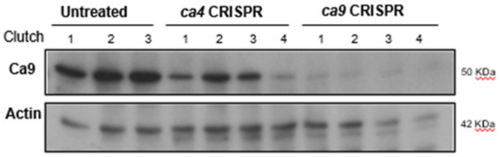

Rabbit anti-CA IX (H-11) for carbonic anhydrase 9 (1:500, #sc-365900 Santa Cruz).

ca-9

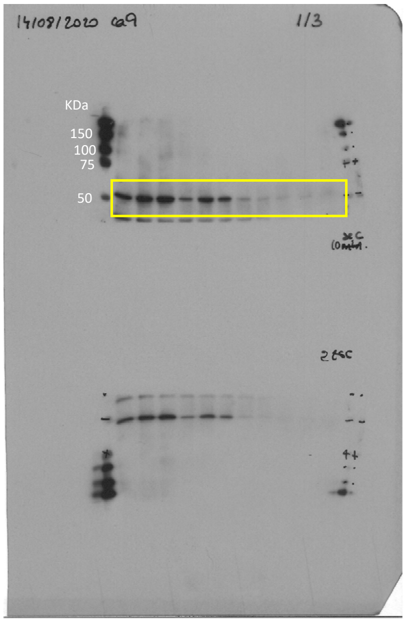

Mouse anti-β-actin (1:1000; #A5316 Sigma-Aldrich).

Actin

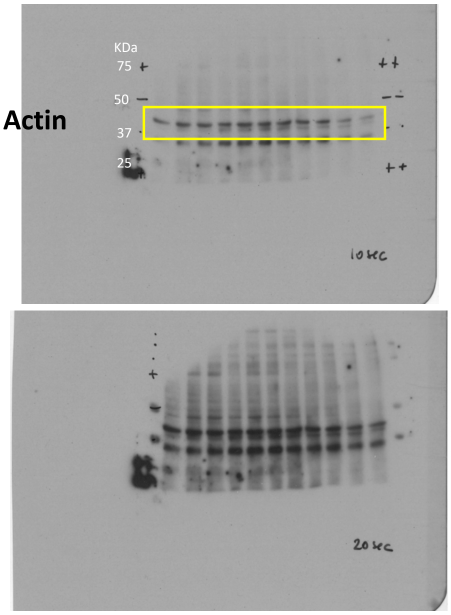

Supplement: Supplementary file 18 — Uncropped scans of blots and gels of western blot data. [file 41589_2024_1762_MOESM18_ESM.pdf]
