# Supplementary material for: Carbonic anhydrase inhibition ameliorates tau toxicity via enhanced tau secretion
Source: Nat Chem Biol. 2024 Oct 31;21(4):577–87. doi: 10.1038/s41589-024-01762-7 (PMC11949835; doi:10.1038/s41589-024-01762-7)
Supplement: Supplementary file 20 — Uncropped scans of blots and gels of western blot data. [file 41589_2024_1762_MOESM20_ESM.pdf]

Ext. Data Fig.5d *used in figure*

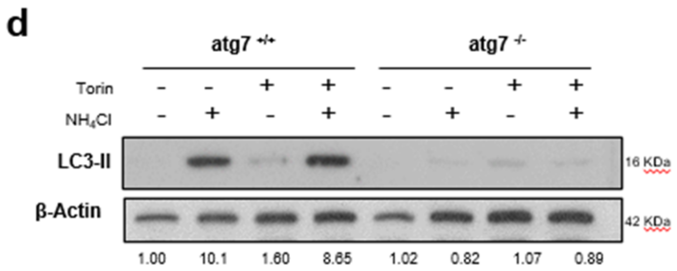

Blots for **TORIN**

Rabbit anti-LC3 antibody (1:1000; #NB100-2220 Novus Biologicals).

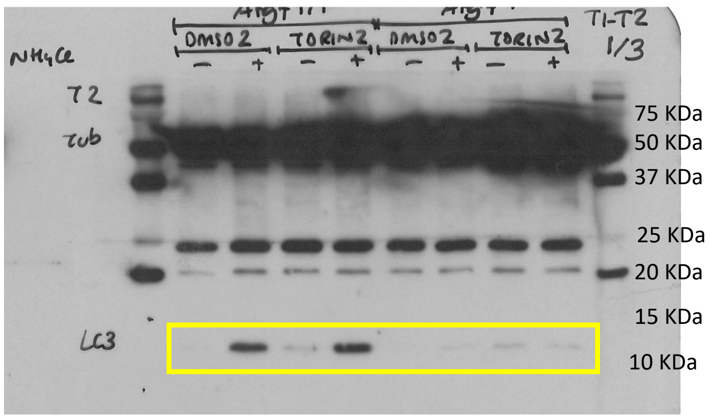

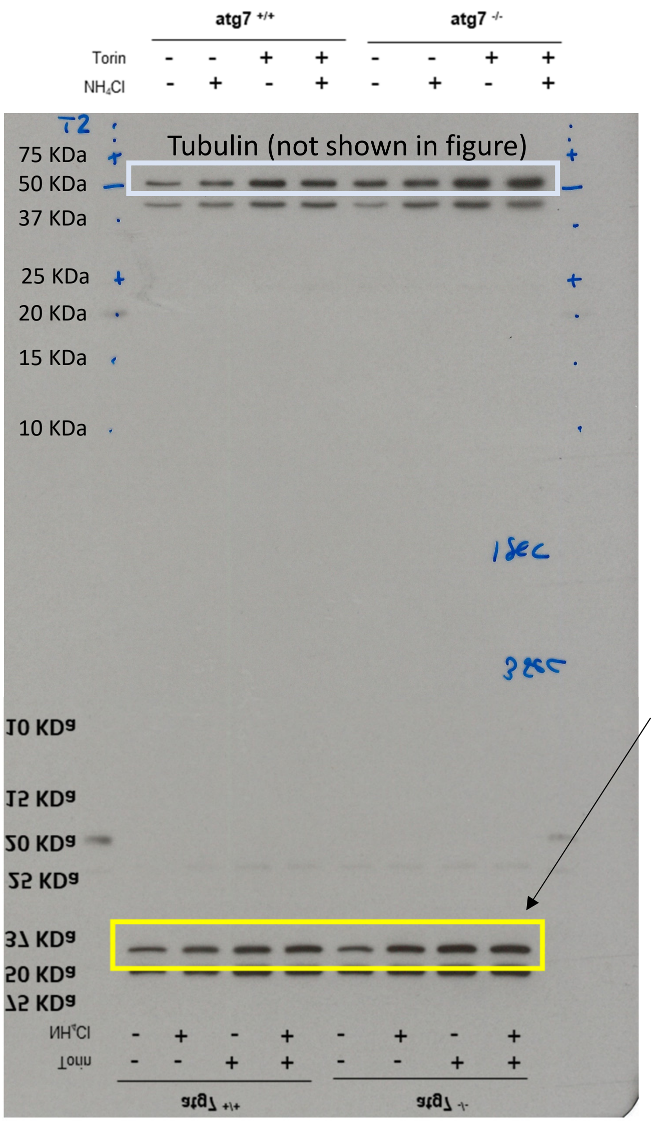

Mouse anti-β-actin (1:1000; #A5316 Sigma-Aldrich).

Exposures for 1 and 3 seconds on the same photography film, flipping between exposures.

Ext. Data Fig.5d

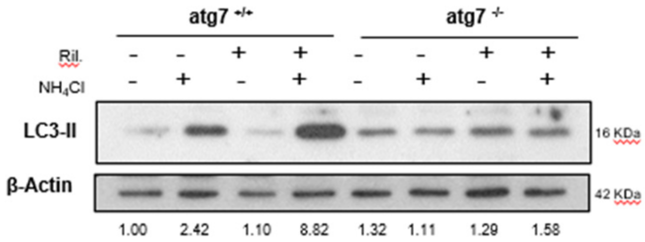

Blots for **Rilmenidine**

Rabbit anti-LC3 antibody (1:1000; #NB100-2220 Novus Biologicals).

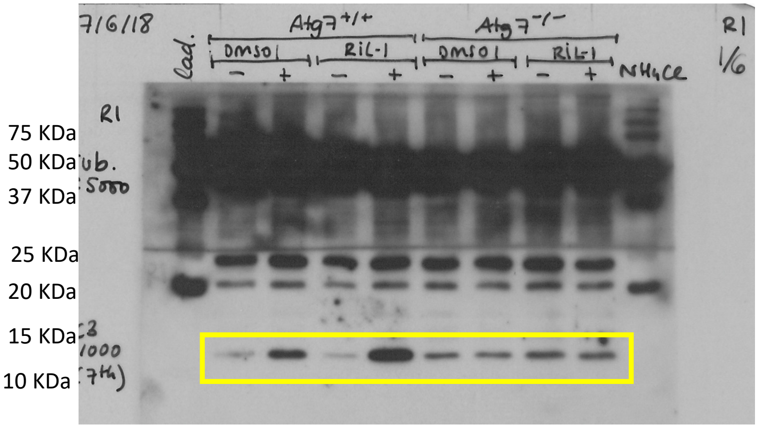

Mouse anti-β-actin (1:1000; #A5316 Sigma-Aldrich).

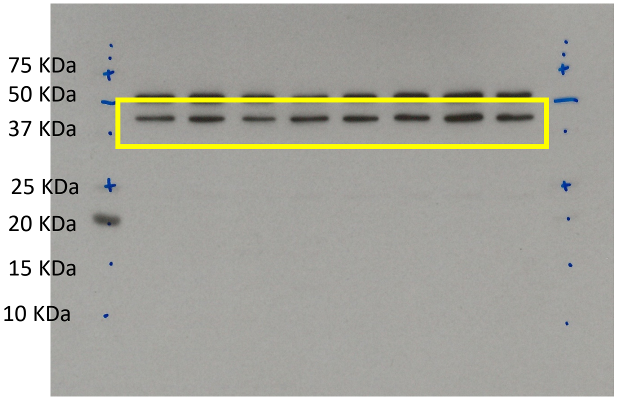

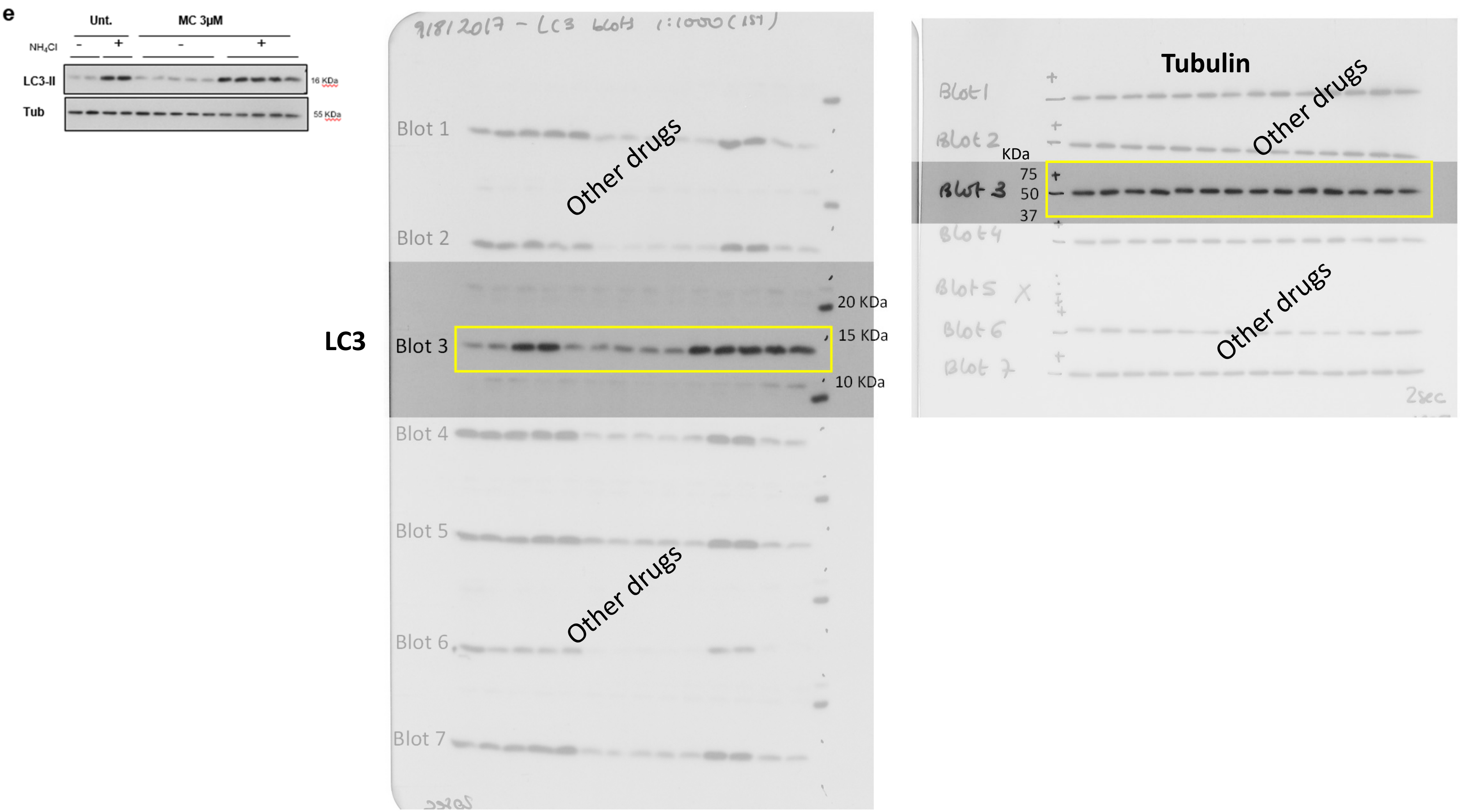

e
Unt.
MC 3μM
NH4Cl
LC3-II
Tub
16 KDa
55 KDa
LC3
Blot 1
Blot 2
Blot 3
Blot 4
Blot 5
Blot 6
Blot 7
Other drugs
Other drugs
20 KDa
15 KDa
10 KDa
Tubulin
Other drugs
Other drugs
KDa
75
50
37
