# Supplementary material for: Carbonic anhydrase inhibition ameliorates tau toxicity via enhanced tau secretion
Source: Nat Chem Biol. 2024 Oct 31;21(4):577–87. doi: 10.1038/s41589-024-01762-7 (PMC11949835; doi:10.1038/s41589-024-01762-7)

Ext Data Fig.8c

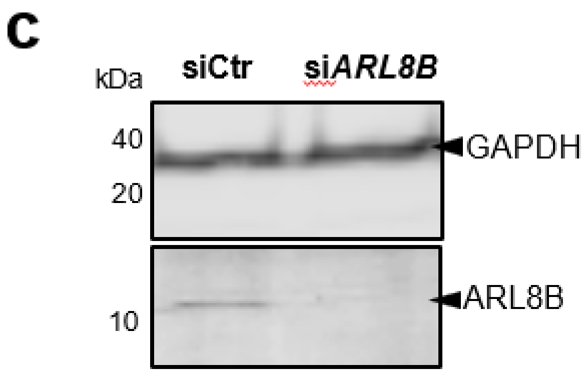

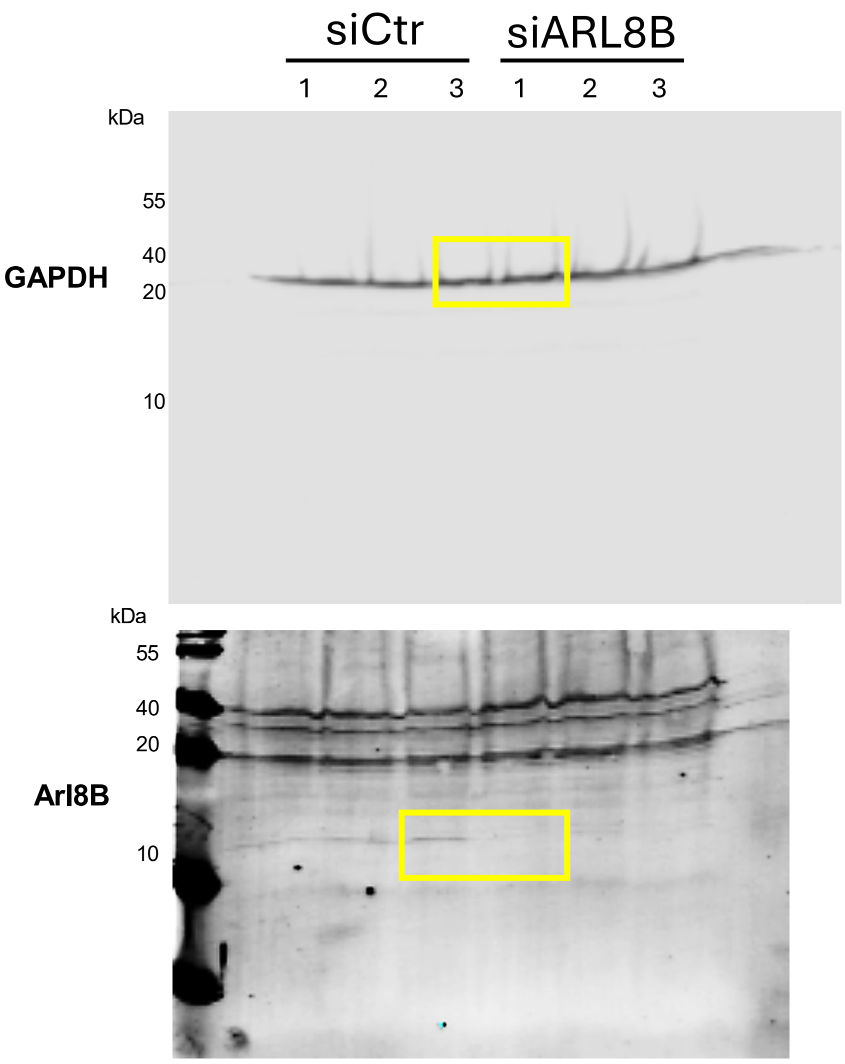

Ext Data Fig.8d

d

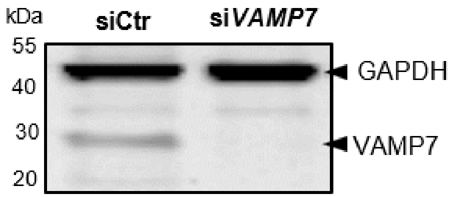

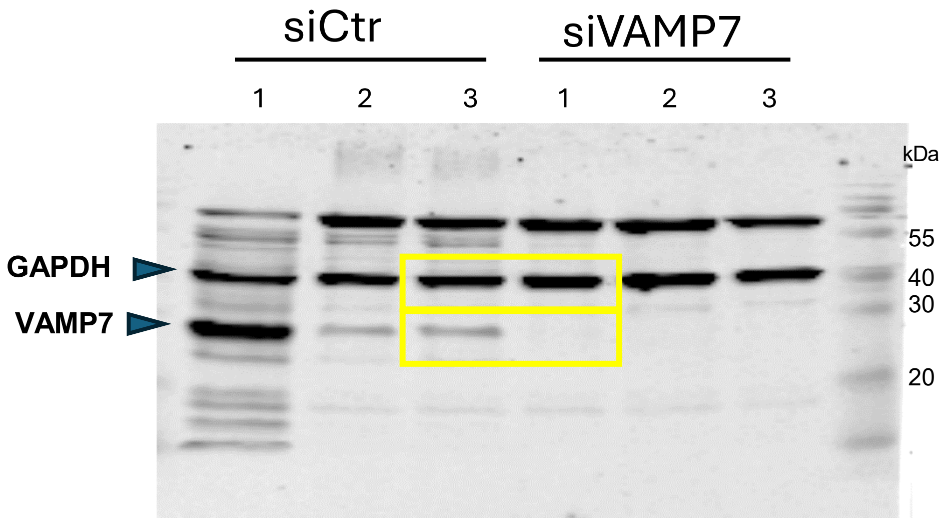

Ext Data Fig.8g

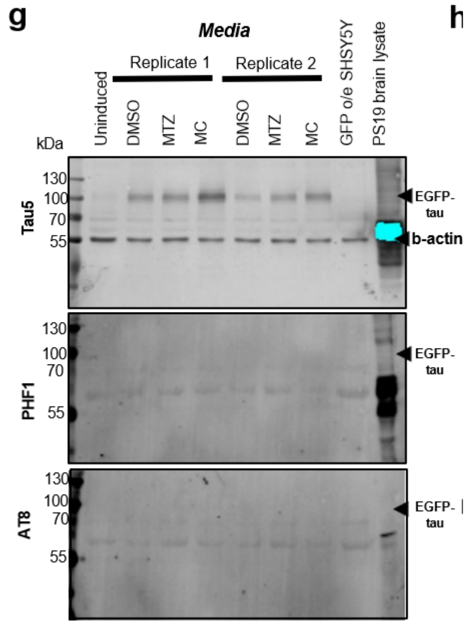

Extended Data Fig 8g top (tau5 and b-actin)

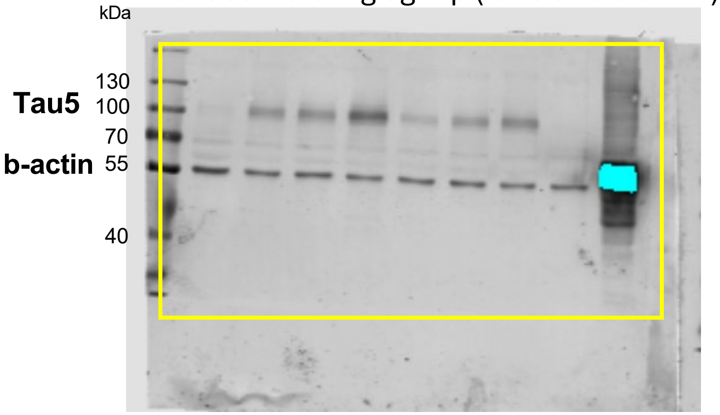

Extended Data Fig 8g middle (PHF1)

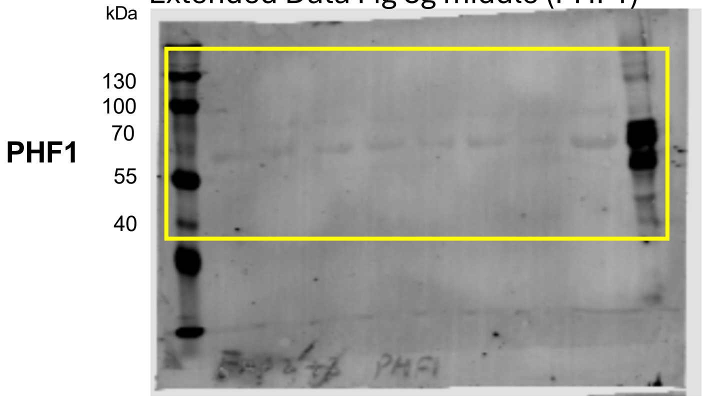

Extended Data Fig 8g bottom (AT8)

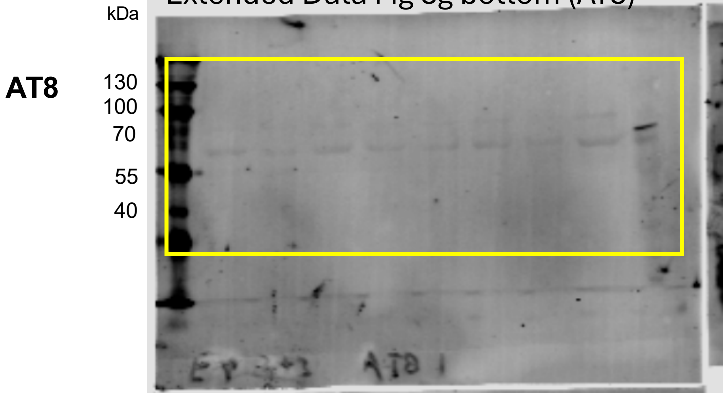

Ext Data Fig.8h

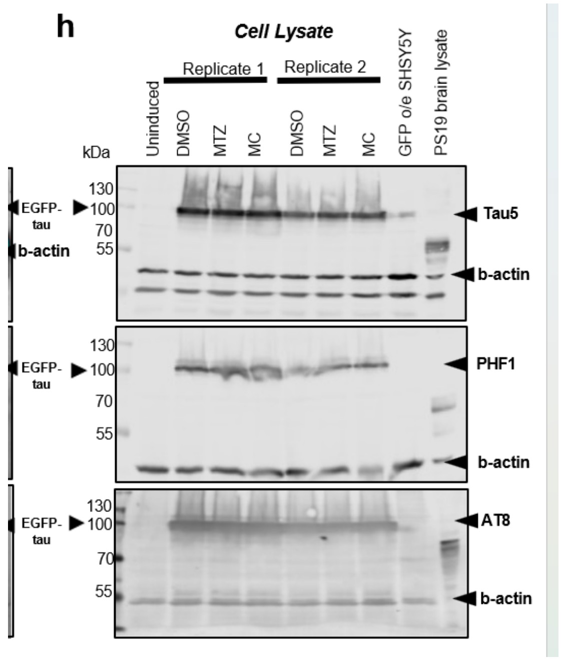

Extended Data Fig 8h top (tau5 and b-actin)

Extended Data Fig 8h middle (PHF1 and b-actin)

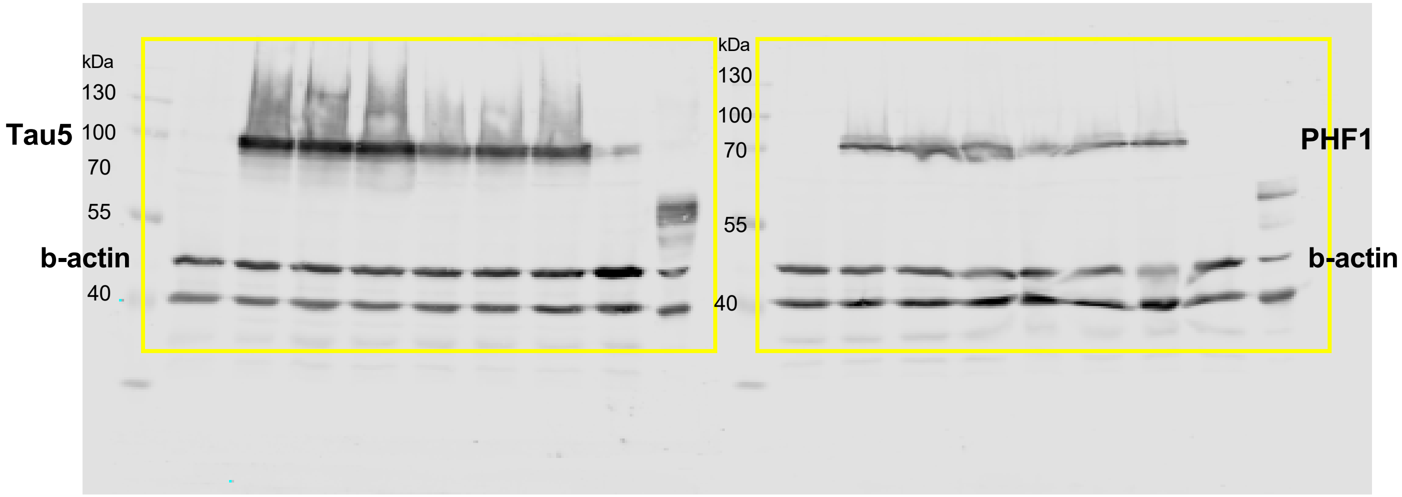

Extended Data Fig 8h bottom (AT8)

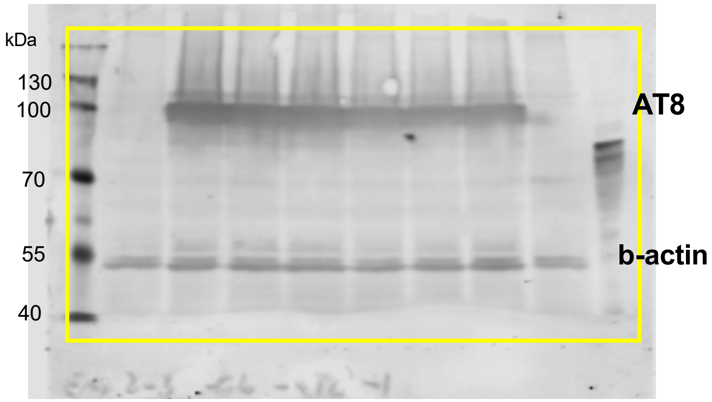

Supplement: Supplementary file 26 — Uncropped scans of blots and gels of western blot data. [file 41589_2024_1762_MOESM26_ESM.pdf]
